# Supplementary material for: Patient perceptions of vulnerability to recurrent respiratory tract infections and prevention strategies: a qualitative study
Source: BMJ Open. 2022 Apr 19;12(4):e055565. doi: 10.1136/bmjopen-2021-055565 (PMC9021765; doi:10.1136/bmjopen-2021-055565)
Supplement: Supplementary data [file bmjopen-2021-055565supp001.pdf]

COREQ checklist

		Item	Guide questions/description	Manuscript section where information can be found (original manuscript page numbers)
**Domain 1: Research team and reflexivity**				
Personal Characteristics	1	Interviewer/facilitator	Which author/s conducted the interview or focus group?	Method- data collection (p7)
	2	Credentials	What was their occupation at the time of the study?	Method- data collection (p7)
	3	Occupation	What was their occupation at the time of the study?	Method- data collection (p7)
	4	Gender	Was the researcher male or female?	Method- data collection (p7)
	5	Experience and training	What experience or training did the researcher have?	Method- data collection (p7)
Relationship with participants	6	Relationship established	Was a relationship established prior to study commencement?	Method- ethics approval and recruitment (p6)
	7	Participant knowledge of the interviewer	What did the participants know about the researcher? e*.g. personal goals, reasons for doing the research*	Method- ethics approval and recruitment (p6)
	8	Interviewer characteristics	What characteristics were reported about the interviewer/facilitator? e.g. *Bias, assumptions, reasons and interests in the research topic*	Method- data collection (p7)<br>Funding statement and competing interests statement (p25)
**Domain 2: study design**				
Theoretical framework	9	Methodological orientation and Theory	What methodological orientation was stated to underpin the study? *e.g. grounded theory, discourse analysis, ethnography, phenomenology, content analysis*	Method- data analysis (p8)
Participant selection	10	Sampling	How were participants selected? *e.g. purposive, convenience, consecutive, snowball*	Method- recruitment (page 6)
	11	Method of approach	How were participants approached? e*.g. face-to-face, telephone, mail, email*	Method- recruitment (page 6)
	12	Sample size	How many participants were in the study?	Method- recruitment (page 6)<br>Table 1 (page 9)
	13	Non-participation	How many people refused to participate or dropped out? Reasons?	n/a

				Not reported in main manuscript for conciseness.<br><br>Our recruitment method does not allow us to know why participants did not respond to our invitation to participate.
	14.	Setting of data collection	Where was the data collected? e.*g. home, clinic, workplace*	Method- data collection (p7)
	15.	Presence of non-participants	Was anyone else present besides the participants and researchers?	Not reported in main manuscript for conciseness.<br><br>Participants were asked to be in a quiet room with no interruptions but we do not know for sure if it was always possible as most interviews were via telephone. Field notes and interview recordings from one face-to-face interview suggest a spouse was present and commenting occasionally.
	16.	Description of sample	What are the important characteristics of the sample? *e.g. demographic data, date*	Method- recruitment (p6) ; Findings- participants (p9), Table 1 (p9)
Data collection	17.	Interview guide	Were questions, prompts, guides provided by the authors? Was it pilot tested?	Method- PPI section (p6), data collection (p7); Online Supplementary Materials 1
	18.	Repeat interviews	Were repeat interviews carried out? If yes, how many?	Method- data collection (p7);
	19.	Audio/visual recording	Did the research use audio or visual recording to collect the data?	Method- data collection (p7)
	20	Field notes	Were field notes made during and/or after the interview or focus group?	Methods- data collection (p7)
	21.	Duration	What was the duration of the interviews or focus group?	Methods- data collection (p7)
	22.	Data saturation	Was data saturation discussed?	The term ‘saturation’ is not reported in main manuscript for conciseness.<br><br>The authors are very cautious about claims of data saturation in thematic analysis (see, for example, arguments in Braun & Clarke, 2021; “To Saturate or not to saturate” Qual Res in Sport, Exercise Health). Data saturation for the current analysis was not aimed for but may have been achieved or approached.<br><br>Recruitment ceased when both RTI experiences were

				well analysed and understood and iterative intervention development was concluded i.e. the research team were satisfied that the interventions were as engaging as possible. Stopping point also required that a range of different viewpoints from patients with different clinical and demographic characteristics had been heard and used. Method- Recruitment - (p6)
	23.	Transcripts returned	Were transcripts returned to participants for comment and/or correction?	n/a<br><br>(member checks with participants were not conducted, professional transcribers transcribed the interviews and researchers checked for accuracy)
**Domain 3: analysis and findings**				
Data analysis	24.	Number of data coders	How many data coders coded the data?	Method- data analysis (p8)
	25.	Description of the coding tree	Did authors provide a description of the coding tree?	Method- data analysis (p8). A coding tree was not used. We present a description of our process of inductive thematic analysis, including the steps we took in coding the data.
	26.	Derivation of themes	Were themes identified in advance or derived from the data?	Method- data analysis (p8).
	27.	Software	What software, if applicable, was used to manage the data?	Method- data analysis (p8).
	28.	Participant checking	Did participants provide feedback on the findings?	n/a<br><br>(member checks were not conducted)
Reporting	29.	Quotations presented	Were participant quotations presented to illustrate the themes / findings? Was each quotation identified? e.*g. participant number*	Findings (p10-22)
	30.	Data and findings consistent	Was there consistency between the data presented and the findings?	Findings (p10-22)
	31.	Clarity of major themes	Were major themes clearly presented in the findings?	Findings(p10-22); Table 2 (p10)
	32.	Clarity of minor themes	Is there a description of diverse cases or discussion of minor themes?	Findings(p10-22); Table 2 (p10)
