# Supplementary material for: Patient perceptions of vulnerability to recurrent respiratory tract infections and prevention strategies: a qualitative study
Source: BMJ Open. 2022 Apr 19;12(4):e055565. doi: 10.1136/bmjopen-2021-055565 (PMC9021765; doi:10.1136/bmjopen-2021-055565)
Supplement: Supplementary data [file bmjopen-2021-055565supp002.pdf]

University of Southampton

Online supplementary Material: Interview Schedules

Section 1: In depth interview about personal experiences of RTIs

1. Can you tell me all about your experience of these sorts of infections [repeat list of RTIs if necessary: colds, flu, coughs, chest infections, bronchitis, ear infections, sinusitis, sore throats, throat infections and tonsillitis].
    - Prompts:
        - Can you tell me about the types of illnesses you tend to get?
        - Can you tell me about when you tend to get these illnesses?
        - What's it like for you when you have them?
        - How often do you get them?
        - How long do they last?

2. Can you tell me about why you think you get these sorts of illnesses?
    - Prompts:
        - Can you tell me about what you think the causes of these illness?
        - Any other reasons why you think you get them?

3. Can you tell me about things you do to try and stop getting these illnesses?
    - Prompts:
        - What made you decide to use these things? Why is it important for you to x/y/z? (e.g. eat healthy, exercise, get the flu jab)
        - How helpful do you find these things?
        - Why do you think they work?

4. When you have these sorts of illnesses is there anything you do to try and make it go away quicker?
    - Prompts
        - Any things you take, or things you do, or avoid doing?
        - What made you decide to use these things? Why is it important for you to x/y/z?
        - How helpful do you find these things?
        - Why do you think they work?

Section 2a:
Intervention Optimisation: Think-aloud interview

Prompts about key pages of intervention content [e.g. menus/ first page etc.]

- What are your first impressions of this page?
- What are you thinking now?
- What made you choose that option?
- What do you think about [this activity, this information, this strategy/tool/idea]?
- Can you tell me a bit more about why you think that?
- [in response to an expression of like/dislike] What is it you like/don't like about that?
- That's really interesting…..
- [picking up on facial expressions/body language/tone of voice etc] I noticed that you frowned/smiled/laughed/sighed etc. at……… Can you tell me what you thought about that?

After working through the key pages of intervention content:

- Overall, what do you think about the web pages?
- Can you tell me about anything you thought was particularly good about the web pages?
- Can you tell me anything about the web pages that you were less keen on?
- Which parts did you find most relevant to you? Which parts were the least relevant to you?
- Having looked at the web pages, can you tell me how you feel about trying to: [tailor to intervention viewed]
    - become more active to try to reduce these sorts of infections
    - reduce stress to try to reduce these sorts of infections
    - use a nasal spray to try to reduce these sorts of infections

- How much of what you've seen today do you think is relevant to coronavirus?
- How at risk do you feel about getting these infections at the moment?
    - [sprays only]. What do you feel about the recommendation to use the spray when at high risk and how this applies to coronavirus?

- We have been thinking about whether to give people optional brief support from their practice nurse to help them follow the advice in the website. This support would likely be 2 x 10-minute phone calls or brief email support
    - What do you think of this idea?
    - What do you like about this idea?
    - What do you dislike about this idea?
    - Do you think you would need any help?
    - [If optional support would be helpful]
    - What would you like to talk to the health professional about?

- In what sort of ways might they be able to help you? Why would this be helpful?
- You could contact the health professional over the phone, and by email. Which one of these options would you prefer? Why?

- What device did you use to look at the website today?
    - If you were using the website over a longer period of time, how would you access the website?
    - Would you use mobile phone at all?

Section 2b
Intervention Optimisation: post-intervention use interview

Version for nasal sprays arm:

- Can you start by telling me overall how you got on with trying the spray?

Questions if they have tried the spray:

- Can you tell me all about how you found using the nasal spray?
- Can you tell me about anything you liked or found easy?
- Can you tell me about anything you disliked or found difficult?
    - Can you let me know if there was anything you found helped with that?
    - Can you tell me about anything that worried you about using the spray?
- Can you tell me about when you used the nasal spray?
    - When did you think to use it?
    - Can you tell me about whether any situations came up where you could have used the spray (e.g. first symptoms, feeling a risk of catching an infection)? Can you tell about how you decided whether to use the spray?
- Can you tell me about what you thought were the advantages of using the nasal spray?
- Can you tell me about what you thought were the disadvantages of using the nasal spray?
- Can you tell me what it's been like for you trying these activities/changes whilst in (partial) lockdown because of coronavirus/COVID-19?
    - Explore the context – have they been self-isolating? Shielding?
    - What have your infections been like during this time? (More/less?)
    - What aspects of lockdown have made it easier to try these activities/manage your infections?
    - What aspects of lockdown have made it harder to try these activities/manage your infections?
    - Can you tell me about any information or advice that was difficult for you to follow during lockdown?

Questions if they have not managed to try the spray:

- Can you tell about what you thought about the idea of using a spray to try to prevent infections?
- Can you tell me about anything about the spray that you liked or found easy?
- Can you tell about anything about the spray that seemed off-putting or difficult for you?
    - Can you tell me about anything that worried you about using the spray?

- Can you tell me anything you feel would help you in the future with trying the spray?
- Can you tell me about whether any situations came up where you could have used the spray (e.g. first symptoms, feeling a risk of catching an infection)?
- Can you tell about how you decided whether to use the spray?
- Can you tell me about what you thought were the advantages of using the nasal spray?
- Can you tell me about what you thought were the disadvantages of using the nasal spray?
- Can you tell me what it's been like for you trying these activities/changes whilst in (partial) lockdown because of coronavirus/COVID-19?
    - Explore the context – have they been self-isolating? Shielding?
    - What have your infections been like during this time? (More/less?)
    - What aspects of lockdown have made it easier to try these activities/manage your infections?
    - What aspects of lockdown have made it harder to try these activities/manage your infections?
    - Can you tell me about any information or advice that was difficult for you to follow during lockdown?

Website questions

- What did you think of website that gave you information and advice about using the nasal spray?
- Can you tell me about anything that you liked about the website?
- Can you tell me about anything that you disliked?
- Can you tell me about anything that you would change in the website?
- Can you tell me about anything that you thought was particularly relevant to you?
- Can you tell me about anything that you thought was not particularly relevant to you?
- Can you tell me about any information or advice that didn't make sense?
- How do you think that could be changed?
- Could you tell me about anything that you thought didn't work properly?
- Can you tell me about whether you went onto the website more than once? (explore why they returned/whether they found what they needed).
- Since looking at the website, how do you feel about infections now?

Instructions

- On the website, it mentions 3 situations where you should use the spray. What did you think about these instructions?
- On the website, it mentions how often to use the spray in each of these 3 situations. What did you think about these instructions? prompting around the instructions.
- On the website, there is a video about how to use the spray. What did you think about this?

- Can you tell me what you thought about the paper booklet about the spray? (repeat questions above as necessary- liked, disliked etc).

Version for lifestyle (physical activity and stress) arm:

Programme/website questions

- Can you start by telling me how you've got on with the programme?
- Can you tell me about the parts of the website that you had a look at?
- Can you talk me through anything you tried out yourself?
- What did you think of:
    - the first few pages of the website, which explained a bit about infections and how getting active/reducing stress might help?
    - the website called 'getting active?
    - the website called 'healthy paths'?
- Can you tell me about anything that you liked?
- Can you tell me about anything that you disliked?
- Can you tell me about anything that you would change?
- Can you tell me about anything that you thought was particularly relevant to you?
- Can you tell me about anything that you thought was not particularly relevant to you?
- Can you tell me about any information or advice that didn't make sense?
    - How do you think that could be changed?
- Could you tell me about anything that you thought didn't work properly?
- Since looking at the website, how do you feel about infections now?

If they didn't look at "Getting Active" or "Healthy Paths":

- What made you decide to focus on only "Getting Active"/only "Healthy Paths"?

Questions regarding activities and tools to help incorporate these into routine:

"Getting Active"

- How did you feel about trying to get more active?
- How did you get on with trying to get more active?
- Can you tell me about anything you found easy about trying to get more active?
- Can you tell about anything you found difficult about trying to get more active?
    - Can you tell me about anything you feel would help you with trying these activities?

- What did you think about the range of activities suggested?
- What do you think the advantages might be for you in trying these activities/making these changes?
- Can you tell me about any disadvantages there might be for you in trying these activities/making these changes?
- Can you tell me what it's been like for you trying these activities/changes whilst in (partial) lockdown because of coronavirus/COVID-19?
    - Explore the context – have they been self-isolating? Shielding?
    - What have your infections been like during this time? (More/less?)
    - What aspects of lockdown have made it easier to try these activities/manage your infections?
    - What aspects of lockdown have made it harder to try these activities/manage your infections?
    - Can you tell me about any information or advice that was difficult for you to follow during lockdown?

"Healthy Paths"

- How did you feel about trying to reduce your stress?
- How did you get on with trying to reduce your stress?
- Can you tell me about anything you found easy about trying to reduce your stress?
- Can you tell me about anything you found difficult about trying to reduce your stress?
- Can you tell me about anything you feel would help you with trying to reduce your stress?
- What did you think about the range of stress reduction techniques suggested?
- What do you think the advantages might be for you in trying these activities/making these changes?
- Can you tell me about any disadvantages there might be for you in trying these activities/making these changes?
- Can you tell me what it's been like for you trying these activities/changes whilst in (partial) lockdown because of coronavirus/COVID-19?
    - Explore the context – have they been self-isolating? Shielding?
    - What have your infections been like during this time? (More/less?)
    - What aspects of lockdown have made it easier to try these activities/manage your infections?
    - What aspects of lockdown have made it harder to try these activities/manage your infections?
    - Can you tell me about any information or advice that was difficult for you to follow during lockdown?
