# Supplementary material for: Patient perceptions of vulnerability to recurrent respiratory tract infections and prevention strategies: a qualitative study
Source: BMJ Open. 2022 Apr 19;12(4):e055565. doi: 10.1136/bmjopen-2021-055565 (PMC9021765; doi:10.1136/bmjopen-2021-055565)
Supplement: Author's manuscript [file bmjopen-2021-055565.draft_revisions.pdf]

BMJ Open is committed to open peer review. As part of this commitment we make the peer review history of every article we publish publicly available.

When an article is published we post the peer reviewers' comments and the authors' responses online. We also post the versions of the paper that were used during peer review. These are the versions that the peer review comments apply to.

The versions of the paper that follow are the versions that were submitted during the peer review process. They are not the versions of record or the final published versions. They should not be cited or distributed as the published version of this manuscript.

BMJ Open is an open access journal and the full, final, typeset and author-corrected version of record of the manuscript is available on our site with no access controls, subscription charges or pay-per-view fees (http://bmjopen.bmj.com).

If you have any questions on BMJ Open's open peer review process please email info.bmjopen@bmj.com

BMJ Open

Patient perceptions of vulnerability to recurrent respiratory tract infections and prevention strategies; a qualitative study

Journal:	*BMJ Open*
Manuscript ID	bmjopen-2021-055565
Article Type:	Original research
Date Submitted by the Author:	11-Aug-2021

Complete List of Authors:	Dennison, Laura; University of Southampton, Centre for Clinical and Community Applications of Health Psychology, Department of Psychology<br>Williamson, Sian; University of Southampton, Centre for Clinical and Community Applications of Health Psychology, Department of Psychology<br>Greenwell, Kate; University of Southampton, Centre for Clinical and Community Applications of Health Psychology, Department of Psychology<br>Handcock, Molly; University of Southampton, School of Primary Care, Population Sciences and Medical Education, Faculty of Medicine,<br>Bradbury, Katherine; University of Southampton, Centre for Clinical and Community Applications of Health Psychology, Department of Psychology; NIHR Applied Research Collaboration (ARC) Wessex<br>Vennik, Jane; University of Southampton , School of Primary Care, Population Sciences and Medical Education, Faculty of Medicine<br>Yardley, Lucy; University of Southampton, Centre for Clinical and Community Applications of Health Psychology, Department of Psychology; University of Bristol<br>Little, Paul; University of Southampton, School of Primary Care, Population Sciences and Medical Education, Faculty of Medicine<br>Geraghty, Adam; University of Southampton, School of Primary Care, Population Sciences and Medical Education, Faculty of Medicine

Keywords:	Respiratory infections < THORACIC MEDICINE, QUALITATIVE RESEARCH, PREVENTIVE MEDICINE, Public health < INFECTIOUS DISEASES, COVID-19, PRIMARY CARE

SCHOLARONE™
Manuscripts

I, the Submitting Author has the right to grant and does grant on behalf of all authors of the Work (as defined in the below author licence), an exclusive licence and/or a non-exclusive licence for contributions from authors who are: i) UK Crown employees; ii) where BMJ has agreed a CC-BY licence shall apply, and/or iii) in accordance with the terms applicable for US Federal Government officers or employees acting as part of their official duties; on a worldwide, perpetual, irrevocable, royalty-free basis to BMJ Publishing Group Ltd ("BMJ") its licensees and where the relevant Journal is co-owned by BMJ to the co-owners of the Journal, to publish the Work in this journal and any other BMJ products and to exploit all rights, as set out in our licence.

The Submitting Author accepts and understands that any supply made under these terms is made by BMJ to the Submitting Author unless you are acting as an employee on behalf of your employer or a postgraduate student of an affiliated institution which is paying any applicable article publishing charge ("APC") for Open Access articles. Where the Submitting Author wishes to make the Work available on an Open Access basis (and intends to pay the relevant APC), the terms of reuse of such Open Access shall be governed by a Creative Commons licence – details of these licences and which Creative Commons licence will apply to this Work are set out in our licence referred to above.

Other than as permitted in any relevant BMJ Author's Self Archiving Policies, I confirm this Work has not been accepted for publication elsewhere, is not being considered for publication elsewhere and does not duplicate material already published. I confirm all authors consent to publication of this Work and authorise the granting of this licence.

For peer review only

Patient perceptions of vulnerability to recurrent respiratory tract infections and prevention strategies; a qualitative study

Laura Dennison[1], Sian Williamson[1], Kate Greenwell[1], Molly Handcock[2], Katherine Bradbury[1,4], Jane Vennik[2], Lucy Yardley[1,3], Paul Little[2] and Adam W A Geraghty[2]

[1]Centre for Clinical and Community Applications of Health Psychology, Faculty of Environmental and Life Sciences, University of Southampton, Southampton, UK; [2]School of Primary Care, Population Sciences and Medical Education, Faculty of Medicine, University of Southampton, Southampton, UK; [3]School of Experimental Psychology, University of Bristol, Bristol, UK. [4]NIHR Applied Research Collaboration (ARC) Wessex, Southampton, UK.

Corresponding Author: Laura Dennison, School of Psychology, University of Southampton, Highfield Campus, Southampton, SO17 1BJ, l.k.dennison@soton.ac.uk

Word count (excluding title page, abstract, references, figures and tables)=5484

Keywords: Respiratory Tract Infections, Common Cold, Influenza, Prevention, Qualitative Research, Interviews, Thematic Analysis

For peer review only

ABSTRACT

Objectives: Respiratory Tract Infections (RTIs) are extremely common, usually self-limiting, but responsible for considerable work sickness absence, reduced quality of life, inappropriate antibiotic prescriptions and healthcare costs. Patients who experience recurrent RTIs and those with certain comorbid conditions have higher personal impact and healthcare costs and may be more likely to suffer disease exacerbations, hospitalisation and death. We explored how these patients experience and perceive their RTIs to understand how best to engage them in prevention behaviours.

Design: A qualitative interview study

Setting: Primary Care, UK

Methods: 23 participants who reported recurrent RTIs and/or had relevant comorbid health conditions were interviewed about their experiences of RTIs. Interviews took place as the COVID-19 pandemic began. Data was analysed using inductive thematic analysis.

Findings: Four themes were developed: *Understanding Causes and Vulnerability, Attempting to Prevent RTIs, Uncertainty and Ambivalence about Prevention, and Changing Experiences because of COVID-19.* Participants' understandings of their susceptibility to RTIs were multifactorial and included both transmission via others and personal vulnerabilities. They engaged in various approaches to try to prevent infections or alter their progression yet perceived they had limited personal control. The COVID-19 pandemic had improved their understanding of transmission, heightened their concern and motivation to avoid RTIs, and extended their repertoire of protective behaviours.

Conclusions: Patients who experience frequent or severe RTIs are likely to welcome and benefit from advice and support regarding RTI prevention. To engage people effectively, those developing interventions or delivering health services must consider their beliefs and concerns about susceptibility and prevention.

For peer review only

ARTICLE SUMMARY: STRENGTHS AND LIMITATIONS OF THIS STUDY

- This study contributes to a limited evidence-base on the experiences of people who suffer recurrent RTIs and/or are at risk of complications, morbidity and mortality as a consequence of RTIs.
- The interview technique and inductive thematic analysis allowed an in-depth exploration of beliefs and experiences, prioritising the participants' perspectives.
- The interview timing coincided with the beginning of the COVID-19 pandemic, allowing interesting observations of how experiencing the pandemic and its restrictions affected people's understandings and behaviour relating to non-pandemic RTIs.
- The purposive sampling strategy ensured that participants had a range of clinical and demographic characteristics but may have failed to capture underserved populations; particularly minority ethnic groups and people with high levels of deprivation.

For peer review only

INTRODUCTION

Respiratory tract infections (RTIs) are infectious diseases of the upper or lower respiratory tract that are usually caused by viruses. RTIs include the common cold, influenza (flu), pharyngitis, tonsillitis, sinusitis and bronchitis along with pandemic infections such as COVID-19. RTIs are common with most adults experiencing at least one cold per year[1]. RTIs typically produce mild to moderate symptoms and are usually self-limiting. Nonetheless they are a leading cause of work sickness absence[2] and reduce health-related quality of life, affecting physical, mental, and social functioning[3]. They are also responsible for substantial healthcare costs, mostly in primary care[4 5]. Consultations for RTIs can also result in unnecessary antibiotics prescribing, contributing to antimicrobial resistance[6 7]. Certain subgroups of patients have worse health outcomes and incur higher healthcare costs from RTIs. This includes patients who experience frequent or recurrent infections and those whose older age and/or comorbid conditions put them at higher risk of disease exacerbations, hospitalisation and death[8 9]. RTI prevention interventions, particularly those focussed on these recurrent and at-risk patients, would benefit individuals and healthcare systems alike.

A wide range of medical and behavioural RTI prevention approaches have been investigated including; hand hygiene[10-12], vaccination[13-15], social distancing[10 16], mask-wearing[10 11 17], vitamins and supplements,[18 19] gargling, nasal rinses and nasal sprays[20], exercise[21 22], and mindfulness meditation[21]. The efficacy for some of these approaches is well-established whilst others still have an unclear evidence base. Interest and urgency around RTI prevention has been prompted by the COVID-19 pandemic. However, in addition to efficacy, prevention interventions must be acceptable to the target population; they must feel personally relevant, feasible and make sense in the context of people's experiences. Therefore, gaining insight into how people understand and experience RTIs and RTI prevention is a vital step in the development and implementation of successful prevention interventions[23].

Many studies have investigated lay perspectives on RTIs, using either qualitative designs or survey methods. Insights include that colds and flu are considered familiar and trivial[24-26], with people often considering themselves to be at low risk of

both contagion and severe outcomes compared to others[25 27-29]. Transmission from symptomatic people is often correctly recognised as important[24 26 29 30]. Confusion may exist about the importance of viruses versus bacteria and the relevance of temperature, damp and pollution[25 30-35]. RTI severity seems to be judged based on symptom duration, discomfort and impact on activities[25 36 37]. People describe attempting to manage RTIs at home[25 32 36 37] with GP consultation triggered by long duration or specific symptoms[25 38] or where symptom resolution in previous illness episodes has been attributed to antibiotics[36 37]. Considerable fatalism exists about catching RTIs[24 26 39 40]. Furthermore, whilst people may be familiar with and adopt prevention approaches such as avoiding infectious people and hand-washing[35 41]. these behaviours can be considered unacceptable and/or unattainable[27 29 40]. People tend to emphasise their own good health, strong personal reserves, and healthy lifestyle as providing defence against RTIs[24 27 30 41]. Experiencing RTIs may even be considered beneficial: a natural, necessary strengthening of the immune system[24 26].

There are some important limitations to the scope of existing research. Firstly, most studies sample the general public, rather than patients with higher infection frequency, morbidity or mortality. Secondly, most studies focus specifically on colds and flu rather than the wider range of RTIs. Furthermore, this literature is dominated by understanding patient perspectives of RTIs specifically in the context of reducing antibiotic prescribing and increasing flu vaccine uptake.

The current paper extends the literature by focusing on patients who get recurrent RTIs and/or are at risk for having more severe consequences of RTIs. We originally aimed to explore how these patients experienced and understood their infections. This research was planned prior to the COVID-19 pandemic and was nested within a NIHR programme grant developing and evaluating RTI prevention approaches. This work was originally driven by the impact of recurrent *non-pandemic* RTIs on patients and healthcare systems. The pandemic, and the resulting requirement for both ‘at risk’ patients and whole populations to adopt and adhere to an array of RTI prevention behaviours, highlights and accelerates the need to understand people’s experiences of and beliefs about RTIs, their vulnerability, and infection prevention behaviours.

For peer review only

METHOD

This study is reported with reference to the COREQ checklist (Supplementary Material 2)

Ethics Approval

Ethics and research governance approvals were granted by NHS and the University of Southampton (REC/HRA19/SC/0354; ERGO:48223).

Patient and Public Involvement (PPI)

A panel of PPI contributors have supported the study planning and conduct, some from the grant application stage. Contributions included editing and improving our participant information sheets, consent forms and interview schedules and participating in pilot interviews to ensure minimal participant burden and improve the question coverage, wording and process.

Recruitment

We recruited adults who had experienced recurrent RTIs (operationalised as ≥3 in the last year and/or ≥1/year over the last 3 years). Because RTIs are common and usually self-limiting, they are not routinely recorded in medical records (only when patients consult). Therefore, a two-step process was used to identify participants. First, three Hampshire (UK) GP practices (a mixture of urban and semi-rural locations and with varying deprivation levels) identified possible participants. They searched their lists and posted invitations and information sheets to a) patients who consulted for ≥1 RTI within the last year and received an antibiotics prescription and b) a sub-group of patients who also had asthma, COPD or chronic sinusitis. Interested patients returned reply slips, on which they self-reported their recent RTI history. We then purposively sampled from the 52 interested patients to achieve a heterogeneous sample based on frequency of self-reported RTIs, health conditions, age and gender. Twenty-three participants were interviewed.

Data collection

Data collection took place between March and August 2020, coinciding with the beginning of the COVID-19 pandemic. Three participants were interviewed face-

For peer review only

to-face in their homes or University premises in early March. The rest participated by telephone during the first UK lockdown or as national restrictions eased.

Participants provided written or online consent prior to taking part. After gathering brief demographic and health data participants were interviewed using a schedule which allowed in depth exploration of their experiences of having RTIs (Table 1) Questions were phrased openly and non-directive prompts were used to elicit extensive discussion. Participants also completed an 'intervention optimisation' section of the interview. Here, their views and experiences of interventions under development as part of a wider project were explored to ensure they were engaging, persuasive and relevant[23]. Participants used and provided feedback on early versions of digital interventions about a) nasal spray use to prevent infections by stopping viruses replicating in the nose and throat, or b) physical activity and stress management to boost immune system functioning (Table 1).

SW (n=20) and LD (n=3) conducted the interviews. Both interviewers were female, with qualitative interviewing and health psychology expertise. Interviews ranged from 41 to 104 minutes (mean=70.5). Interviewers took field notes. Interviews were audio-recorded then transcribed verbatim. Participants received a £10 retail voucher to thank them for their time.

Data Analysis

An inductive thematic analysis was conducted from a critical realist perspective[42]. LD led the analysis with input from SW, KG, JV, AG and MH. The analysts have health psychology, medicine or healthcare research backgrounds.

The analysis primarily drew on data from interview section 1 (see Table 1). Findings from Sections 2 and 3 were primarily used for iterative intervention optimisation. However, data relating to the research question of the current study was included in this paper's analysis.

First, familiarisation with the data was achieved through listening to audio-recordings and reading transcripts. Next, thorough line-by-line coding began, facilitated by NVivo 12. Descriptive labels (codes) were attached to words or phrases that captured ideas relating to the research questions. Codes were reviewed frequently, and definitions were developed and iteratively edited. The analysis proceeded to examine the codes and develop them into themes which captured patterns and features of the data. This involved an iterative process of clustering together or merging similar codes and splicing or dividing codes, all the time engaging in constant comparison between codes and transcripts and searching for deviant cases. Themes were iteratively reviewed, refined, organised and relabelled, with the input of the wider team, to ensure the final set of themes and subthemes were insightful and coherent themes.

For peer review only

Table 1: Overview of interview sections

Interview section	N participants completing the section	Interview coverage and procedure
**Section 1.** **In depth interview about personal experiences of RTIs**	23 (all)	Participants were prompted to share details of their personal experiences of having RTIs, including the types of infections they experience, how infections affect them, when they experience infections, why they believe they experience them, how they manage them and if/how they attempt to avoid them. Later interviews also explored the impact of COVID-19 and the pandemic restrictions on their experiences of RTIs. (see Interview Schedule Section 1: Online Supplementary Material 1)
**Section 2a** **INTERVENTION OPTIMISATION:** **Think-aloud interview**	16[1] Of which: 10 nasal spray 6 lifestyle change (physical activity and stress management)	Participants were emailed a link to a digital intervention and worked through it whilst simultaneously being interviewed by a researcher. They were prompted to verbalise their thoughts and feelings as they encountered different pages, sections, messages, images and features. (see Interview Schedule Section 2a: Online Supplementary Material 1)
**Section 2b** **INTERVENTION OPTIMISATION:** **Longitudinal interview**	9[1] Of which: 5 nasal spray 4 lifestyle change (physical activity and stress management)	Participants were emailed a link to a digital intervention and used it independently over a period of 2-3 weeks. They then participated in an interview about their experiences of using the digital intervention, and of trying out the RTI infection prevention behaviours it recommends. (see Interview Schedule Section 2b: Online Supplementary Material 1)

[1] Two participants took part in both think aloud and longitudinal interviews (both nasal sprays)

For peer review only

FINDINGS

Participants

Table 2 shows participant characteristics. Participants reported a mean of 3 infections in the previous year. Ten participants had asthma, three had COPD, and three had chronic sinusitis. More females than males participated. Age ranged between 18 and 83.

Table 2: Demographic and clinical characteristics of participants (n=23)

	Mean (SD), range or N (%)
**Age (years)**	53.87 (19.62), range 18-83
**Gender**	
Male	6 (26.09%)
Female	17 (73.91%)
**Marital status**	
Married or living with partner	11 (47.83%)
Single	5 (21.74%)
Divorced	4 (17.39%)
Widowed	3 (13.04%)
**Employment status**	
In paid work (full or part time, employed, self-employed)	10 (43.48%)
	6 (26.09%)
Retired	2 (8.70%)
Not working because of illness/disability	5 (21.74%)
Other (unemployed, homemakers, students, volunteers)	
**Education (age left education)**	
16 or before	3 (13.04%)
17 or 18	6 (26.09%)
Over 18	14 (60.87%)
**Deprivation (2019 Index of Multiple Deprivation Decile[1])**	Median= 9 (IQR 6.5), range 2-10
**Health Conditions[2]**	
Asthma	10 (43.48%)
COPD	3 (13.04%)
Chronic Sinusitis	3 (13.04%)
None of these conditions	12 (52.17%)
**Number of RTIs in last 12 months**	3.23 (1.6), range 1-6
**RTIs per year in last 3 years**	
≥1	21 (91.3%)
≥3	15 (65.2%)

For peer review only

Types of RTIs experienced at least once in last 12 months	
Cold	17 (73.91%)
Flu	4 (17.39%)
Throat infection	13 (56.52%)
Chest infection	12 (52.17%)
Sinus infection	10 (43.48%)
Ear infection	4 (17.39%)
**Concern about RTIs** (0-10)[3]	5.36 (3.09), 0-10
**Consequences of RTIs** (0-10)[3]	6.04 (3.08), 1-10

[1] (1=most deprived, 10 is least deprived), data available for 17/23 participants; [2] 4 participants had more than one of these health conditions; [3] Brief illness Perception Questionnaire (B-IPQ) concern and consequences items, higher scores indicate higher concern and worse impact.

For peer review only

Themes and subthemes

Four themes and 12 subthemes were developed from the data (Table 3). These are explained below, alongside illustrative quotations.

Table 3: Themes and Subthemes

**Themes**	**Subthemes**
Understanding causes and vulnerability	Catching RTIs from other people<br>Something in the environment<br>Defective bodies and inadequate defences<br>Wondering why
Attempting to prevent RTIs	Trying (desperately) not to get RTIs<br>Hygiene measures<br>Avoiding symptomatic people<br>Keeping yourself strong and healthy<br>Nipping infections in the bud
Uncertainty & ambivalence about prevention	Inevitability and limited control<br>Judging whether things help<br>Not letting it dominate
Changing experiences because of COVID-19.	

Understanding causes and vulnerability

Participants had complex, multidimensional understandings about why RTIs occur and how and why they are more susceptible than others.

Catching RTIs from other people

All participants understood that RTIs were transmitted between people, identifying people and situations that they considered pertinent for their own infections. Some discussions included the relevance of symptomatic people, highlighting coughs and sneezes as transmission mechanisms and occasionally drawing on the concepts of germs and viruses.

> If you're next to somebody who's coughing and spluttering, the chances are you'll end up with the same. (P21_female_66yrs).

Participants emphasised proximity to others as important for spreading infection and physical contact as particularly risky.

> [On the Underground] you're like sardines in a can and if there's anything going you're going to catch it (P9_male_83yrs)
>
> Where people are hugging and kissing. That's when these things get passed around. (P23_female_53yrs)

A few participants also described getting infections from contaminated objects and surfaces.

Many participants emphasised children as frequent and significant sources of infection; "little germ machines" (P23_female_53yrs), "efficient little bug spreaders" (P21_female_66yrs), "super-spreaders" (P4_female_67yrs). They emphasised children's poor respiratory hygiene, "particularly revolting and snotty" (P21_female_66yrs).They were described as having "constantly got something (P14_female_18yrs)", and being likely to pass infections on to their families "bringing infections into the house from school" (P9_male_83yrs). Children posed particular problems for older participants who were wary of catching RTIs.

For peer review only

> [My grandchildren] had a cold and I didn't say anything because they are glad to come down to see us, but I was thinking they were sat on that [chair] and I was thinking 'please don't get any closer'. (P1_Male_66yrs)

Several participants highlighted their current or past occupation as incurring high RTI risk. They described exposure to large numbers or certain types of people (e.g. children, students, general public), or close contact (e.g. a tattoo artist), as well as commuting on public transport.

Something in the environment

Many participants referred to environmental influences as responsible for their RTIs. Season was referenced as a very obvious factor by many although a few described having year-round infections.

> Most of the winter months, I am like, 'Oh, I've got a cold again.' (P2_female_37yrs)

A few considered exposure to cold air and/or temperature *change* as causing infections and/or provoking symptoms.

> Maybe when I go out and I don't keep warm enough, so my chest is exposed (P14_female_18yrs)

> Going out into the cold, coming back to the heat, and I think that's just where people get colds from. (P8_female_53yrs)

A minority described mixing more with people indoors as relevant to seasonality and, consistent with their understandings of transmission via people, participants sometimes referred to breathing virus-contaminated air. A few participants perceived pollution, damp or sensitivity to triggers in the atmosphere (e.g. deodorants, cleaning products) as potential causes of their RTIs. Those who emphasised poor air quality and environmental triggers tended to also discuss having asthma and/or allergies.

Defective bodies and inadequate defences

In addition to the causal factors mentioned above, most participants perceived that deficiencies within their bodies had created vulnerabilities to RTIs. Many participants

For peer review only

discussed picking up infections more readily than others, experiencing them more severely and finding them harder to shift.

> I just seem to pick up everything. (P4_female_67yrs)

> [My husband] doesn't tend to suffer as badly as I do. We joke about it that I make more fuss, but it isn't that. Mine just goes on for longer and is always worse. (P12_female_72yrs).

Participants described how ageing and age-related lifestyle influenced RTI vulnerability. A few of the older participants spoke of their advanced age as weakening their ability to fight off infections.

> When you're older you're just not as strong or it's not as easy to shrug these things off. (P1_male_66yrs)

However, some older people found that ageing-related lifestyle changes and retirement had reduced exposure to people, making them *less* susceptible to picking them up.

Several participants felt their chronic health conditions, or a previous severe RTI meant their body did not fight off RTIs effectively, describing weakened defences or immune systems.

> It's just a little bit weaker and just that little kind of chink in its armour is enough to let infections in whereas previously [before asthma] I was a bit healthy and robust and the infection would be fought off instantly by my own immune system. Now, it's just a little bit tired and it just can't cope quite as well. (P18_female_57yrs)

> The very first one [RTI] has made me susceptible […] I seem to now not get them [RTIs] regularly, but when I get one, I get them very badly. (P10_female_62yrs)

> My immune system is rock bottom at the moment where I've just come out the other side of [a lengthy recovery from a severe RTI] so that makes me, in my head, more vulnerable. I think I could easily pick up something else. (P15_female_51yrs)

For peer review only

Some discussed how past medical treatments, may have left them with weakened defences. P4 (female_67yrs) described radical sinus surgery over 30 years ago that had left her with "no natural defences at all" and P12 (female_72yrs) suspected that her treatment for a thyroid condition had affected her immune system.

Some participants believed part of their body was weakened ("a slight weakness on the throat", P3_male_72yrs), malfunctioning ("There's something, somewhere in me, that decides it's going to not work properly", P7_female_81yrs), or malformed ("something wonky in my nose", P17_female_41yrs).

Participants also discussed the importance of stress. Many participants believed that experiencing upsetting, difficult experiences made *people in general* more likely to suffer RTIs (and other illnesses) or make your RTI more severe. A few participants considered positive mental health to be protective.

> The happier you are, and the more well-being you have, I think you generally fight things off more quickly. (P4_female_67yrs)

Many also considered stress pertinent to their personal experience of recurrent or severe RTIs.

> That's why I succumbed to being so unwell because of the stress that I was under. I think that was a massive, played a massive part. (P15_female_51yrs)

A few participants felt that vulnerability tended to occur *after* stress or busyness.

> As soon as you started to relax, you'd get a bug or something. You'd get a cold. (P17_female_41yrs)

In contrast, some participants did not perceive a role for stress in getting infections. Some emphasised an absence of stress (particularly older participants) "I haven't nowadays got anything to be stressed about" (P12_female_72yrs) or a lack of stress reactivity "I'm not that sort of person" (P17_female_41yrs). Some of these participants, however, discussed busyness and exhaustion as personally pertinent; emphasising being 'run down', rather than 'stressed'.

> If you're not feeling great anyway, you're tired etc., etc., and you are rundown a little bit, I think you're kind of more susceptible to catch these germs (P1_Male_66yrs)

For peer review only

Wondering why

Despite offering the explanations outlined above, some participants simultaneously referred to *not* fully understanding why they suffered with RTIs. Some expressed a sense of mystery surrounding becoming ill, especially being unable to trace the infection's origin.

> There seems to be no rhyme or reason to it because nobody else seems to be ill around me, it just seems to be me. (P7_female_81yrs)

Other comments appeared to reflect a sense of injustice; that their infections are undeserved given their personal choices and behaviour.

> *[Interviewer: Why do you think you get these infections?]* Participant: That's something I ask myself many, many times. I've got friends who are older than me and they don't suffer like I do. I've got friends who are younger than me. They go out drinking. They smoke. They also indulge in other things. They don't suffer like me. (P22_female_73yrs)

Attempting to prevent RTIs

Almost all participants wanted to prevent RTIs and were taking steps to try to protect themselves.

Trying (desperately) not to get RTIs

Some participants' attempts to avoid getting RTIs involved multiple, complex behaviours and regimes.

> When you know that you have a tendency to be poorly when you catch things, you just do everything you can to not catch them. (P21_female_66yrs).

Many RTI prevention strategies were long-term habits, often learned in childhood (e.g. hand and respiratory hygiene). These behaviours were considered obvious and given little thought, "It's just been second nature" (P15_female_51yrs).

Participants also described their repertoire of RTI prevention behaviours as evolving over time, with new products and strategies adopted following recommendations

For peer review only

from family, friends, and healthcare professionals. Participants were open to and enthusiastic about trying prevention approaches

> I would be open to trying anything new that people suggest. (P5_female_24yrs)

> You can get really fed up of getting ill and you can be like desperate to latch on to anything. (P13_female_25yrs)

Hygiene measures

Most participants described hygiene measures to prevent germs getting into their system. Handwashing was almost universally reported, usually as something obvious ("All the normal hygiene, hand-washing that sort of thing", P17_female_41yrs). Some mentioned handwashing very briefly, others described specifics of their meticulous regimes. A few described other hygiene measures including cleaning objects and safe tissue disposal.

> If someone comes in and they say they're feeling a bit run down then I'll make sure that I've sterilised and cleaned the room and my hands properly. (P5_female_24yrs)

Mask wearing and ventilation were discussed infrequently. A few people mentioned avoiding touching their face as important, but difficult. In general, participants described their exemplary hygiene behaviour but highlighted poor practices of others.

> They've sneezed, and I felt it on the back of my head. (P1_Male_66yrs)

> I wish more people did it [handwashing]. (P23_female_53yrs)

Avoiding symptomatic people

A key prevention strategy our participants adopted was avoiding symptomatic people. Most described deliberately avoiding anyone displaying or declaring cold symptoms.

> I will take a very wide berth (P10_female_62yrs)

> I'm just, 'Stay away from me'. (P1_male_66yrs)

For peer review only

Although some were extremely vigilant about avoiding ill people, (“everybody knew not to come and visit if they had so much as a sniffle” (P23_female_53yrs), other participants described how this is impossible or undesirable (see below *Not Letting it Dominate*).

Keeping yourself strong and healthy

Many participants described undertaking health-enhancing behaviours such as eating healthily, taking vitamins, keeping physically fit, nurturing psychological wellbeing, not smoking, alcohol avoidance and staying hydrated. However, these behaviours were rarely adopted or sustained with preventing RTIs as the end goal. Instead they were long-term habits or preferences (“That's what I've always done. That is who I am”, P4_female_67yrs), “It's good life practice, P6_male_78yrs), or related to healthy ageing or managing other health conditions. Nonetheless, participants believed or hoped that these healthy behaviours would provide some protection from RTIs.

> I would hope that it means that I'm a bit more resilient to fighting them off. (P21_female_66yrs)

Unlike the behaviours mentioned above, a few behaviours were adopted specifically for preventing RTIs. Several participants discussed the flu vaccination (with mixed uptake and views on efficacy and safety). A few ensured they kept warm in cold weather. Several participants with asthma or COPD described ‘keeping on top of’ their medications, monitoring indicators of health conditions (e.g. peak flow) or having a medication escalation regime in place.

> It just keeps your body at an even keel, don't it? Because if you let your medication drop, then your body immune system will drop as well. (P8_female_53yrs)

Nipping infections in the bud

Besides attempts to *prevent* infections, many participants discussed taking prompt action when RTI symptoms began and attempting to alter the infection’s course.

> When I know [a] cold's coming [..] I try and stop it going any further (P17_female_41yrs)

For peer review only

> If I'm out and about and I don't deal with a suspected cold quickly, it will take hold. (P10_female_62yrs)

Through experience, participants recognised early infection symptoms and subsequently tried to 'nip infections in the bud', stopping symptoms progressing and worsening. Some found this was at least sometimes effective. The behaviours considered helpful varied and included early use of over-the-counter cold and flu remedies, extra vitamins or supplements, gargling, nasal rinses and nasal sprays, or swift GP consultation for antibiotics.

> With a sore throat, sometimes I just go straight home and gargle […] I get the tickle, get the thing, it goes away in a couple of days (P3_Male_72yrs)

> I'll start trying to do those a little bit more to try and counteract perhaps catching things. (P17_female_41yrs)

> Obviously, I try and get some antibiotics. If I can get them early, I can stop it getting hold. If it gets hold, there's nothing I can do. (P22_female_73yrs)

Participants with asthma and COPD discussed altering medications in response to suspected beginnings of infections.

> I do "up" [increase] my steroid inhaler, so that kind of gives my lungs a boost before the illness strikes and then can usually fend things off that way, but if I don't feel it coming on, then I have to fight it once it arrives, which is slightly harder. (P18_female_57yrs)

Uncertainty and ambivalence about prevention

Most participants discussed the extent to which RTI prevention seems possible or desirable.

Inevitability and limited control

Despite their concerted prevention efforts, most participants described feeling somewhat powerless over contracting infections, with some discussing the inevitability of symptoms worsening and progressing.

> You don't have any control over it. It's just going to get you (P8_female_53yrs)

For peer review only

> You can do all these things to try and prevent but if you're going to get something you're going to get it I think! (P15_female_51yrs)

> I've done everything I possibly can to try and prevent it happening and not making it as bad as what it is, but it just never seems to work, and I always end up at the doctor's with steroids, antibiotics. (P11_female_19yrs)

Central to the perceived inevitability was the understanding that RTIs come from other people. Problems arose because is not always obvious who has infections ("things are out there, and you don't know who's got it, P15_female_51yrs), nor is it always possible or desirable to avoid people. Participants often described feeling at the mercy of other people's behaviour and hygiene.

> I've got all my systems are all in place to avoid all those things. It's only really when I step out anywhere else that I'm in an environment, that I can't control (P23_female_53yrs)

> I can't control everybody else, the snotty noses and the likes. (P2_female_37yrs)

Judging whether things help

As discussed in the previous themes, sometimes participants had experienced perceptible reductions in RTIs as a result of nipping infections in the bud or avoiding people (see previous theme). However, more often participants described uncertainty about whether the strategies they adopted were helpful. Some seemed indifferent to scrutinising helpfulness, lacking curiosity about habitual behaviours. Some spoke of the difficulties of evaluating their effectiveness.

> Whether they do any good or not I have no idea, because I feel exactly the same as I did before (P13_female_25yrs)

> I suppose I'd have to not take them [vitamins] for a year and then try and see the difference (P10_female_62yrs).

Sometimes participants abandoned strategies that did not produce obvious benefits but sometimes they persevered through habit or hope.

> I'm assuming they're doing something! (P19_female_42yrs)

For peer review only

> I'm going to continue doing it because it might make a difference soon, or one day. (P11_female_19yrs)

Not letting it dominate

Participants often discussed how a balance between RTI prevention and quality of life must be achieved. Some RTI avoidance behaviours were considered undesirable or almost impossible, particularly those that involved social distancing from even asymptomatic people.

> You can't put yourself into a bubble and do nothing. That would be a very destructive way of living. Then, you become victimised by being unwell which is no good to anybody. (P4_Female_67yrs)

> There's the trade-off isn't there? With quality of life. (P23_female_53yrs)

Whilst some felt so vulnerable that they considered it essential to be 'hypervigilant', 'so careful' and 'a little bit oversensitive' (P23_female_53yrs), several participants expressed wariness of becoming, or being perceived as, a hypochondriac.

> There's a fine line between taking care and allowing your concerns to impact on your life. (P21_female_66yrs)

Changing experiences because of COVID-19.

Participants' perceptions of RTIs and prevention behaviours were influenced by the context of the COVID-19 pandemic. Participants recognised COVID-19 as an RTI, albeit one that is unfamiliar and particularly severe, creating heightened motivation for RTI prevention.

> Particularly now, you need to be armed to the teeth with everything you can to protect yourself! (P23_female_53yrs)

Several participants were fearful, perceiving themselves to be at risk of severe COVID-19 due to the same vulnerabilities that they understood predisposed them to other RTIs.

> I'm paranoid. Absolutely paranoid about getting it because I can't breathe very well anyway (P15_female_51yrs)

For peer review only

Participants recognised similarities in transmission mechanisms between COVID-19 and non-pandemic RTIs and described developing better understandings about spread of infections and increasing insight into how to stop transmission, particularly hygiene measures.

> Before this, I wasn't so aware of how illnesses travel, especially a cough and a sneeze [..] and how long it stays on the surface. (P14_female_18yrs)

> We're all used to using these hand sanitisers and we understand how they're protecting us. (P23_female_53yrs)

> I've definitely learned through coronavirus that I touch my face all the time. (P10_female_62yrs)

However, their sense that their health was at the mercy of other people appeared heightened; participants were extremely conscious and critical of other people's behaviour.

> They're mixing with people and then still going out and about the shops like their normal day-to-day living, and that's the scary bit. They don't understand that they might not have it, but they might be a carrier, and carry it to people like me. (P8_female_53yrs)

During national lockdown, shielding and other restrictions, participants' usual behaviour of distancing themselves from *symptomatic* people changed to distancing from almost everyone. Consequently, participants experienced an unequivocally successful RTI prevention method, often for the first time.

> Where you're not mixing with people, you're not catching colds! (P21_female_66yrs)

Some participants found social distancing and isolation burdensome ("I'm questioning where that trade-off is comfortable", P23_female_53yrs). Nonetheless some had discovered approaches that they speculated adopting in the longer term for 'normal' RTIs.

> Being maybe more cautious in social interactions, to prevent catching things anyway. Maybe we'll all be like that! (P20_male_59yrs)

For peer review only

DISCUSSION

Novel contributions and implications

This study extends the literature by exploring how a recurrent and at-risk patient group experiences and understands their RTIs, including if and how they can be prevented. Some findings echoed those from previous research but several important new insights emerged. These may be useful for determining how to successfully engage these patients in RTI prevention behaviours and are therefore discussed in these terms below.

Like previous studies on colds and flu[24-26 29 30], our participants demonstrated a largely accurate understanding of RTI transmission. Their identification of children as important infectors (for non-pandemic RTIs) came out particularly strongly in this study, compared to past research. Alongside their correct understandings of virus transmission, many participants held concurrent beliefs about the causal role of temperature, damp, allergens and pollution. Such beliefs have been noted previously, but sometimes considered erroneous, folklore, or culture-specific.[31] However, these factors could be relevant; modest evidence suggests that cold and damp could be causally linked to RTIs[43 44]. Furthermore, it is widely accepted that respiratory viruses thrive in cold and damp environments. Another explanation is that patients may confuse a causal role of these factors with their tendency to trigger or exacerbate respiratory symptoms from their chronic conditions (e.g. coughs and wheezes from asthma or allergies). Addressing causal beliefs may be necessary if an intervention relies on accurate understandings of virus transmission.

Unlike some earlier studies[27 29] our patients were highly motivated to prevent RTIs and engaged in many relevant behaviours. Consistent with their grasp of transmission mechanisms, they focused (even pre-COVID-19) on avoiding contact with symptomatic others and undertaking hygiene measures. Previous literature, in contrast, suggested such measures might be considered unfeasible, unnecessary or unacceptable[27 29 41]. Our participants were open to prevention recommendations and described extending their prevention repertoire over time. Furthermore, some were considering continuing with COVID-19 measures such as social distancing and mask-wearing to avoid routine RTIs in the future. It therefore seems these recurrent and at-risk patients will be highly receptive to additional support with infection

For peer review only

prevention. The differences in motivation and acceptance of prevention behaviours between the current study and previous research likely reflects our sampling of a higher risk/higher impact patient group but may also reflect the heightened concern due to the COVID-19 context. Contrary to previous research where RTIs have often been described as trivial and participants have not considered themselves susceptible to catching RTIs or vulnerable to severe consequences[24 26 27 29] most of our participants perceived a personal vulnerability to frequent and/or severe infections. Some described how RTIs were linked to medical conditions, stress was salient for others, some had vague ideas about weak immune systems, and other showed uncertainty and frustration about vulnerability. Interventions that offer an acceptable explanatory framework for recurrent infections, help people understand their personal vulnerability, and provide a corresponding, coherent prevention strategy may prove validating and helpful to these patients.

As in some previous studies[40 41], some participants described engaging in behaviours to stay healthy and strong. However, our analysis highlighted how most of these efforts appeared to be engaged in through enjoyment, habit or in the service of managing other health conditions. Few participants appeared to have consciously considered and committed to boosting their immune system to prevent RTIs, though, for example, increased physical activity, adequate sleep and stress reduction. No participants mentioned recommendations from health professionals to make lifestyle changes for RTI prevention. Patients suffering from recurrent or severe RTIs should be given information about how these behaviours relate to immune health. They may benefit from support and encouragement with initiating, stepping up, and maintaining these behaviours.

Despite seeking to avoid RTIs, participants had some doubts about whether prevention is indeed possible or desirable and were unclear about the extent to which their strategies were helping them. Fatalism about RTIs has been identified previously[24 26 41] but, importantly, here participants describe helplessness despite considerable prevention efforts; the idea of being at the mercy of other people's risky and inconsiderate behaviour is apparent. Interventions will need to improve outcome expectancies by convincing people that prevention approaches will be effective, and helping people to identify situations and behaviours where they can exert personal control, despite the role of other people in infection transmission. A novel finding was

For peer review only

that many participants believed that they sometimes succeeded in stopping RTIs developing or worsening through rapid intervention when they begin. These anecdotes suggest people perceive their own actions sometimes alter the progression of RTIs. Drawing on these experiences might help people to develop confidence about other approaches to preventing infections. Finally, participants expressed concerns about prevention behaviours dominating life. Concerns about prevention strategies may require more detailed research, including worries or barriers specific to particular approaches. Acknowledging and addressing concerns and careful communication and framing may be necessary to make prevention interventions acceptable.

Strengths and limitations

Whilst our recruitment strategy successfully identified recurrent and at risk RTI patients, the sample was self-selecting and nesting our data collection within an RTI prevention intervention development study may have attracted participants who were particularly interested in prevention. This may limit the transferability of findings. Furthermore, we are unlikely to have included the experiences of underserved populations. Although we recruited from practices serving a variety of communities and included 6 participants with IMD deciles <4, the sample was skewed towards low deprivation. No ethnicity data was collected. This is a limitation, especially given the increasing awareness of the unequal susceptibility to and impact of COVID-19[45-48]. Capturing the perspectives of underserved populations who are also unequally burdened by the impact of RTIs remains a priority.

Conclusion

Our participants described multifaceted causal understandings of their recurrent and/or severe RTIs, including both transmission from others and their own perceived weakened defences. Despite trying various strategies to prevent infections, RTIs persisted and they often described feeling powerless to influence infections. People with a history of recurrent RTIs seem likely to welcome prevention interventions but may need to be persuaded that approaches will be effective, drawing on a convincing and personally meaningful rationale for how interventions will help them. Our study shows how COVID-19 has facilitated learning about RTI spread, heightened feelings of vulnerability but also exposed participants to highly effective

For peer review only

prevention strategies. The current heightened concern and awareness about infections may be fertile ground for introducing prevention approaches for non-pandemic infections. Future research should explore how people respond to interventions to help them reduce RTI frequency and severity.

Author Contributions

AG, LY and PL conceived the study idea and initial study design with later input from KB, LD, KG, SW and JV. SW and LD collected the data. LD led data analysis with analytic and interpretive input from all authors at different stages. LD drafted the manuscript and all authors contributed to critically editing and approving the final manuscript.

Other contributors: Kate Martinson manged ethical approvals and recruitment. Thank you also to our public contributors Hazel Patel, Debs Smith and Samantha Richards-Hall.

Competing interests: none declared.

Funding: This study was nested within a NIHR Programme Grant for Applied Research: REducing Common infections in Usual practice for Recurrent Respiratory tract Infections (RECUR). (Prof Paul Little, Dr Adam Geraghty) [RP-PG-0218-20005]. Lucy Yardley is an NIHR Senior Investigator and her research programme is partly supported by NIHR Applied Research Collaboration (ARC)-West, NIHR Health Protection Research Unit (HPRU) for Behavioural Science and Evaluation, and the NIHR Southampton Biomedical Research Centre (BRC).

Online Supplementary Materials

1. Interview schedule
2. COREQ Checklist

For peer review only

REFERENCES

1. Heikkinen T, Järvinen A. The common cold. *Lancet* 2003;361(9351):51-59. doi: 10.1016/s0140-6736(03)12162-9
2. ONS. Sickness absence in the UK labour market: 2020 2021 [Available from: https://www.ons.gov.uk/employmentandlabourmarket/peopleinwork/labourproductivity/articles/sicknessabsenceinthelabourmarket/2020.
3. Linder JA, Singer DE. Health-related quality of life of adults with upper respiratory tract infections. *J Gen Intern Med* 2003;18(10):802-07. doi: 10.1046/j.1525-1497.2003.21246.x
4. Finley CR, Chan DS, Garrison S, et al. What are the most common conditions in primary care? Systematic review. *Can Fam Physician* 2018;64(11):832-40. [published Online First: 2018/11/16]
5. Morbey RA, Harcourt S, Pebody R, et al. The burden of seasonal respiratory infections on a national telehealth service in England. *Epidemiol Infect* 2017;145(9):1922-32. doi: 10.1017/s095026881700070x
6. Gulliford MC, Dregan A, Moore MV, et al. Continued high rates of antibiotic prescribing to adults with respiratory tract infection: survey of 568 UK general practices. *BMJ Open* 2014;4(10):e006245. doi: 10.1136/bmjopen-2014-006245
7. Nowakowska M, Van Staa T, Mölter A, et al. Antibiotic choice in UK general practice: rates and drivers of potentially inappropriate antibiotic prescribing. *J Antimicrob Chemother* 2019;74(11):3371-78. doi: 10.1093/jac/dkz345
8. Nam HH, Ison MG. Respiratory syncytial virus infection in adults. *BMJ* 2019;366:l5021. doi: 10.1136/bmj.l5021
9. Satia I, Cusack R, Greene JM, et al. Prevalence and contribution of respiratory viruses in the community to rates of emergency department visits and hospitalizations with respiratory tract infections, chronic obstructive pulmonary disease and asthma. *PLOS ONE* 2020;15(2):e0228544. doi: 10.1371/journal.pone.0228544
10. Jefferson T, Del Mar CB, Dooley L, et al. Physical interventions to interrupt or reduce the spread of respiratory viruses. *Cochrane Database Syst Rev* 2020 doi: 10.1002/14651858.cd006207.pub5
11. Saunders-Hastings P, Crispo JAG, Sikora L, et al. Effectiveness of personal protective measures in reducing pandemic influenza transmission: A systematic review and meta-analysis. *Epidemics* 2017;20:1-20. doi: 10.1016/j.epidem.2017.04.003
12. Warren-Gash C, Fragaszy E, Hayward AC. Hand hygiene to reduce community transmission of influenza and acute respiratory tract infection: a systematic review. *Influenza Other Respir Viruses* 2013;7(5):738-49. doi: 10.1111/irv.12015
13. Lee JKH, Lam GKL, Shin T, et al. Efficacy and effectiveness of high-dose versus standard-dose influenza vaccination for older adults: a systematic review and meta-analysis. *Expert Rev Vaccines* 2018;17(5):435-43. doi: 10.1080/14760584.2018.1471989
14. Polack FP, Thomas SJ, Kitchin N, et al. Safety and Efficacy of the BNT162b2 mRNA Covid-19 Vaccine. *N Engl J Med* 2020;383(27):2603-15. doi: 10.1056/nejmoa2034577
15. Voysey M, Clemens SAC, Madhi SA, et al. Safety and efficacy of the ChAdOx1 nCoV-19 vaccine (AZD1222) against SARS-CoV-2: an interim analysis of four randomised controlled trials in Brazil, South Africa, and the UK. *Lancet* 2021;397(10269):99-111. doi: 10.1016/s0140-6736(20)32661-1
16. Hayward AC, Beale S, Johnson AM, et al. Public activities preceding the onset of acute respiratory infection syndromes in adults in England - implications for the use of social distancing to control pandemic respiratory infections. *Wellcome Open Res* 2020;5:54. doi: 10.12688/wellcomeopenres.15795.1

For peer review only

17. Brainard J, Jones NR, Lake IR, et al. Community use of face masks and similar barriers to prevent respiratory illness such as COVID-19: a rapid scoping review. *Euro Surveill* 2020;25(49) doi: 10.2807/1560-7917.es.2020.25.49.2000725
18. Martineau AR, Jolliffe DA, Hooper RL, et al. Vitamin D supplementation to prevent acute respiratory tract infections: systematic review and meta-analysis of individual participant data. *BMJ* 2017:i6583. doi: 10.1136/bmj.i6583
19. Van Driel ML, Beller EM, Thielemans E, et al. Oral vitamin C supplements to prevent and treat acute upper respiratory tract infections. *Cochrane Database Syst Rev* 2019 doi: 10.1002/14651858.cd013292
20. Kramer A, Eggers M, Hübner N, et al. Virucidal gargling and virucidal nasal spray. *GMS Hyg Infect Control* 2021 doi: 10.3205/dgkh000373
21. Barrett B, Hayney MS, Muller D, et al. Meditation or Exercise for Preventing Acute Respiratory Infection: A Randomized Controlled Trial. *Ann Fam Med* 2012;10(4):337-46. doi: 10.1370/afm.1376
22. Grande AJ, Keogh J, Silva V, et al. Exercise versus no exercise for the occurrence, severity, and duration of acute respiratory infections. *Cochrane Database Syst Rev* 2020;2020(4) doi: 10.1002/14651858.cd010596.pub3
23. Yardley L, Morrison L, Bradbury K, et al. The Person-Based Approach to Intervention Development: Application to Digital Health-Related Behavior Change Interventions. *J Med Internet Res* 2015;17(1):e30. doi: 10.2196/jmir.4055
24. Lundgren B. The Common Cold, Influenza and Immunity in Post-Pandemic Times: Lay representations of Self and Other among older people in Sweden. *Health, Culture and Society* 2015;8(2):46-59. doi: 10.5195/hcs.2015.200
25. Mayrhuber EA-S, Peersman W, Van De Kraats N, et al. "With fever it's the real flu I would say": laypersons' perception of common cold and influenza and their differences - a qualitative study in Austria, Belgium and Croatia. *BMC Infect Dis* 2018;18(1) doi: 10.1186/s12879-018-3568-9
26. Yardley L, Miller S, Teasdale E, et al. Using Mixed Methods to Design a Web-based Behavioural Intervention to Reduce Transmission of Colds and Flu. *J Health Psychol* 2011;16(2):353-64. doi: 10.1177/1359105310377538
27. Cedraschi C, Saya L, Klein P, et al. Representations of influenza and influenza-like illness in the community - a qualitative study. *BMC Fam Pract* 2013;14(1):15. doi: 10.1186/1471-2296-14-15
28. Seale H, Mak JP, Razee H, et al. Examining the knowledge, attitudes and practices of domestic and international university students towards seasonal and pandemic influenza. *BMC Public Health* 2012;12(1):307. doi: 10.1186/1471-2458-12-307
29. Teasdale E, Santer M, Geraghty AWA, et al. Public perceptions of non-pharmaceutical interventions for reducing transmission of respiratory infection: systematic review and synthesis of qualitative studies. *BMC Public Health* 2014;14(1):589. doi: 10.1186/1471-2458-14-589
30. Prior L, Evans MR, Prout H. Talking about colds and flu: The lay diagnosis of two common illnesses among older British people. *Soc Sci Med* 2011;73(6):922-28. doi: 10.1016/j.socscimed.2010.09.054
31. Baer RD, Weller SC, De Alba Garca JG, et al. Cross-Cultural Perspectives on Physician and Lay Models of the Common Cold. *Med Anthropol Q* 2008;22(2):148-66. doi: 10.1111/j.1548-1387.2008.00012.x
32. Braun B, Fowles J, Solberg L, et al. Patient Beliefs About the Characteristics, Causes, and Care of the Common Cold. *J Fam Pract* 2000;49(2):153.
33. Sigelman CK. Age and Ethnic Differences in Cold Weather and Contagion Theories of Colds and Flu. *Health Educ Behav* 2012;39(1):67-76. doi: 10.1177/1090198111407187

For peer review only

34. Snell L, Wilson R, Oeffinger K, et al. Patient and physician explanatory models for acute bronchitis. *J Fam Pract* 2002;51(12):1035-40.
35. Telford R. What influences elderly peoples' decisions about whether to accept the influenza vaccination? A qualitative study. *Health Educ Res* 2003;18(6):743-53. doi: 10.1093/her/cyf059
36. Leydon GM, McDermott L, Thomas T, et al. 'Well, it literally stops me from having a life when it's really bad': a nested qualitative interview study of patient views on the use of self-management treatments for the management of recurrent sinusitis (SNIFS trial). *BMJ Open* 2017;7(11):e017130. doi: 10.1136/bmjopen-2017-017130
37. McSweeney LA, Rousseau NS, Wilson JA, et al. Stakeholders' views of recurrent sore throat, tonsillitis and their management: a qualitative interview study for the NAtional Trial of Tonsillectomy IN Adults (NATTINA Part 1). *Clin Otolaryngol* 2017;42(2):301-06. doi: 10.1111/coa.12720
38. Mortazhejri S, Patey AM, Stacey D, et al. Understanding determinants of patients' decisions to attend their family physician and to take antibiotics for upper respiratory tract infections: a qualitative descriptive study. *BMC Fam Pract* 2020;21(1) doi: 10.1186/s12875-020-01196-9
39. Alqahtani AS, Sheikh M, Wiley K, et al. Australian Hajj pilgrims' infection control beliefs and practices: Insight with implications for public health approaches. *Travel Med Infect Dis* 2015;13(4):329-34. doi: 10.1016/j.tmaid.2015.05.009
40. Davis MDM, Stephenson N, Lohm D, et al. Beyond resistance: social factors in the general public response to pandemic influenza. *BMC Public Health* 2015;15(1) doi: 10.1186/s12889-015-1756-8
41. Petricek G, Hoffmann K, Vandenbroucke A, et al. Laypersons' perception of common cold and influenza prevention—a qualitative study in Austria, Belgium and Croatia. *Eur J Gen Pract* 2019;25(4):220-28. doi: 10.1080/13814788.2019.1645831
42. Collier A. Critical Realism: An Introduction to Roy Bhaskar's Philosophy. London: Verso 1994.
43. Johnson C, Eccles R. Acute cooling of the feet and the onset of common cold symptoms. *Family Practice* 2005;22(6):608-13. doi: 10.1093/fampra/cmi072
44. Sauni R, Verbeek JH, Uitti J, et al. Remediating buildings damaged by dampness and mould for preventing or reducing respiratory tract symptoms, infections and asthma. *Cochrane Database Syst Rev* 2015 doi: 10.1002/14651858.cd007897.pub3
45. Chaudhuri K, Chakrabarti A, Lima JM, et al. The interaction of ethnicity and deprivation on COVID-19 mortality risk: a retrospective ecological study. *Sci Rep* 2021;11(1) doi: 10.1038/s41598-021-91076-8
46. Daras K, Alexiou A, Rose TC, et al. How does vulnerability to COVID-19 vary between communities in England? Developing a Small Area Vulnerability Index (SAVI). *J Epidemiol Community Health* 2021:jech-2020-21522. doi: 10.1136/jech-2020-215227
47. Razai MS, Kankam HKN, Majeed A, et al. Mitigating ethnic disparities in covid-19 and beyond. *BMJ* 2021:m4921. doi: 10.1136/bmj.m4921
48. Whitehead M, Barr B, Taylor-Robinson D. Covid-19: We are not "all in it together"—less privileged in society are suffering the brunt of the damage. *BMJ Opinion* 2020. https://blogs.bmj.com/bmj/2020/05/22/covid-19-we-are-not-all-in-it-together-less-privileged-in-society-are-suffering-the-brunt-of-the-damage/.

For peer review only

UNIVERSITY OF Southampton

Online supplementary Material 1: Interview Schedules

Section 1: In depth interview about personal experiences of RTIs

1. Can you tell me all about your experience of these sorts of infections [repeat list of RTIs if necessary: colds, flu, coughs, chest infections, bronchitis, ear infections, sinusitis, sore throats, throat infections and tonsillitis].
   - Prompts:
     - Can you tell me about the types of illnesses you tend to get?
     - Can you tell me about when you tend to get these illnesses?
     - What's it like for you when you have them?
     - How often do you get them?
     - How long do they last?

2. Can you tell me about why you think you get these sorts of illnesses?
   - Prompts:
     - Can you tell me about what you think the causes of these illness?
     - Any other reasons why you think you get them?

3. Can you tell me about things you do to try and stop getting these illnesses?
   - Prompts:
     - What made you decide to use these things? Why is it important for you to x/y/z? (e.g. eat healthy, exercise, get the flu jab)
     - How helpful do you find these things?
     - Why do you think they work?

4. When you have these sorts of illnesses is there anything you do to try and make it go away quicker?
   - Prompts
     - Any things you take, or things you do, or avoid doing?
     - What made you decide to use these things? Why is it important for you to x/y/z?
     - How helpful do you find these things?
     - Why do you think they work?

For peer review only

UNIVERSITY OF Southampton

Section 2a:
Intervention Optimisation: Think-aloud interview

Prompts about key pages of intervention content [e.g. menus/ first page etc.]

- What are your first impressions of this page?
- What are you thinking now?
- What made you choose that option?
- What do you think about [this activity, this information, this strategy/tool/idea]?
- Can you tell me a bit more about why you think that?
- [in response to an expression of like/dislike] What is it you like/don't like about that?
- That's really interesting…..
- [picking up on facial expressions/body language/tone of voice etc] I noticed that you frowned/smiled/laughed/sighed etc. at……… Can you tell me what you thought about that?

After working through the key pages of intervention content:

- Overall, what do you think about the web pages?
- Can you tell me about anything you thought was particularly good about the web pages?
- Can you tell me anything about the web pages that you were less keen on?
- Which parts did you find most relevant to you? Which parts were the least relevant to you?
- Having looked at the web pages, can you tell me how you feel about trying to: [tailor to intervention viewed]
    - become more active to try to reduce these sorts of infections
    - reduce stress to try to reduce these sorts of infections
    - use a nasal spray to try to reduce these sorts of infections

- How much of what you've seen today do you think is relevant to coronavirus?
- How at risk do you feel about getting these infections at the moment?
    - [sprays only]. What do you feel about the recommendation to use the spray when at high risk and how this applies to coronavirus?

- We have been thinking about whether to give people optional brief support from their practice nurse to help them follow the advice in the website. This support would likely be 2 x 10-minute phone calls or brief email support
    - What do you think of this idea?
    - What do you like about this idea?
    - What do you dislike about this idea?
    - Do you think you would need any help?
    - [If optional support would be helpful]
    - What would you like to talk to the health professional about?

UNIVERSITY OF Southampton

  - In what sort of ways might they be able to help you? Why would this be helpful?
  - You could contact the health professional over the phone, and by email. Which one of these options would you prefer? Why?
- What device did you use to look at the website today?
  - If you were using the website over a longer period of time, how would you access the website?
  - Would you use mobile phone at all?

For peer review only

UNIVERSITY OF Southampton

Section 2b
Intervention Optimisation: longitudinal interview

Version for nasal sprays arm:

- Can you start by telling me overall how you got on with trying the spray?

Questions if they have tried the spray:

- Can you tell me all about how you found using the nasal spray?
- Can you tell me about anything you liked or found easy?
- Can you tell me about anything you disliked or found difficult?
    - Can you let me know if there was anything you found helped with that?
    - Can you tell me about anything that worried you about using the spray?
- Can you tell me about when you used the nasal spray?
    - When did you think to use it?
    - Can you tell me about whether any situations came up where you could have used the spray (e.g. first symptoms, feeling a risk of catching an infection)? Can you tell about how you decided whether to use the spray?
- Can you tell me about what you thought were the advantages of using the nasal spray?
- Can you tell me about what you thought were the disadvantages of using the nasal spray?
- Can you tell me what it's been like for you trying these activities/changes whilst in (partial) lockdown because of coronavirus/COVID-19?
    - Explore the context – have they been self-isolating? Shielding?
    - What have your infections been like during this time? (More/less?)
    - What aspects of lockdown have made it easier to try these activities/manage your infections?
    - What aspects of lockdown have made it harder to try these activities/manage your infections?
    - Can you tell me about any information or advice that was difficult for you to follow during lockdown?

Questions if they have not managed to try the spray:

- Can you tell about what you thought about the idea of using a spray to try to prevent infections?
- Can you tell me about anything about the spray that you liked or found easy?
- Can you tell about anything about the spray that seemed off-putting or difficult for you?
    - Can you tell me about anything that worried you about using the spray?

For peer review only

UNIVERSITY OF Southampton

- Can you tell me anything you feel would help you in the future with trying the spray?
- Can you tell me about whether any situations came up where you could have used the spray (e.g. first symptoms, feeling a risk of catching an infection)?
- Can you tell about how you decided whether to use the spray?
- Can you tell me about what you thought were the advantages of using the nasal spray?
- Can you tell me about what you thought were the disadvantages of using the nasal spray?
- Can you tell me what it's been like for you trying these activities/changes whilst in (partial) lockdown because of coronavirus/COVID-19?
    - Explore the context – have they been self-isolating? Shielding?
    - What have your infections been like during this time? (More/less?)
    - What aspects of lockdown have made it easier to try these activities/manage your infections?
    - What aspects of lockdown have made it harder to try these activities/manage your infections?
    - Can you tell me about any information or advice that was difficult for you to follow during lockdown?
    -

<u>Website questions</u>

- What did you think of website that gave you information and advice about using the nasal spray?
- Can you tell me about anything that you liked about the website?
- Can you tell me about anything that you disliked?
- Can you tell me about anything that you would change in the website?
- Can you tell me about anything that you thought was particularly relevant to you?
- Can you tell me about anything that you thought was not particularly relevant to you?
- Can you tell me about any information or advice that didn't make sense?
- How do you think that could be changed?
- Could you tell me about anything that you thought didn't work properly?
- Can you tell me about whether you went onto the website more than once? (explore why they returned/whether they found what they needed).
- Since looking at the website, how do you feel about infections now?

<u>Instructions</u>

- On the website, it mentions 3 situations where you should use the spray. What did you think about these instructions?
- On the website, it mentions how often to use the spray in each of these 3 situations. What did you think about these instructions? prompting around the instructions.
- On the website, there is a video about how to use the spray. What did you think about this?

For peer review only

UNIVERSITY OF Southampton

- Can you tell me what you thought about the paper booklet about the spray? (repeat questions above as necessary- liked, disliked etc).

<u>Version for lifestyle (physical activity and stress) arm:</u>

<u>Programme/website questions</u>

- Can you start by telling me how you've got on with the programme?
- Can you tell me about the parts of the website that you had a look at?
- Can you talk me through anything you tried out yourself?
- What did you think of:
    - the first few pages of the website, which explained a bit about infections and how getting active/reducing stress might help?
    - the website called 'getting active?
    - the website called 'healthy paths'?
- Can you tell me about anything that you liked?
- Can you tell me about anything that you disliked?
- Can you tell me about anything that you would change?
- Can you tell me about anything that you thought was particularly relevant to you?
- Can you tell me about anything that you thought was not particularly relevant to you?
- Can you tell me about any information or advice that didn't make sense?
    - How do you think that could be changed?
- Could you tell me about anything that you thought didn't work properly?
- Since looking at the website, how do you feel about infections now?

<u>If they didn't look at "Getting Active" or "Healthy Paths":</u>

- What made you decide to focus on only "Getting Active"/only "Healthy Paths"?

<u>Questions regarding activities and tools to help incorporate these into routine:</u>

"Getting Active"

- How did you feel about trying to get more active?
- How did you get on with trying to get more active?
- Can you tell me about anything you found easy about trying to get more active?
- Can you tell about anything you found difficult about trying to get more active?
    - Can you tell me about anything you feel would help you with trying these activities?

For peer review only

- What did you think about the range of activities suggested?
- What do you think the advantages might be for you in trying these activities/making these changes?
- Can you tell me about any disadvantages there might be for you in trying these activities/making these changes?
- Can you tell me what it's been like for you trying these activities/changes whilst in (partial) lockdown because of coronavirus/COVID-19?
    - Explore the context – have they been self-isolating? Shielding?
    - What have your infections been like during this time? (More/less?)
    - What aspects of lockdown have made it easier to try these activities/manage your infections?
    - What aspects of lockdown have made it harder to try these activities/manage your infections?
    - Can you tell me about any information or advice that was difficult for you to follow during lockdown?

"Healthy Paths"

- How did you feel about trying to reduce your stress?
- How did you get on with trying to reduce your stress?
- Can you tell me about anything you found easy about trying to reduce your stress?
- Can you tell me about anything you found difficult about trying to reduce your stress?
- Can you tell me about anything you feel would help you with trying to reduce your stress?
- What did you think about the range of stress reduction techniques suggested?
- What do you think the advantages might be for you in trying these activities/making these changes?
- Can you tell me about any disadvantages there might be for you in trying these activities/making these changes?
- Can you tell me what it's been like for you trying these activities/changes whilst in (partial) lockdown because of coronavirus/COVID-19?
    - Explore the context – have they been self-isolating? Shielding?
    - What have your infections been like during this time? (More/less?)
    - What aspects of lockdown have made it easier to try these activities/manage your infections?
    - What aspects of lockdown have made it harder to try these activities/manage your infections?
    - Can you tell me about any information or advice that was difficult for you to follow during lockdown?

For peer review only

Online Supplementary Material 2: COREQ checklist

		Item	Guide questions/description	Manuscript section where information can be found
**Domain 1: Research team and reflexivity**				
Personal Characteristics	1	Interviewer/facilitator	Which author/s conducted the interview or focus group?	Method- data collection
	2	Credentials	What was their occupation at the time of the study?	Method- data collection
	3	Occupation	What was their occupation at the time of the study?	Method- data collection
	4	Gender	Was the researcher male or female?	Method- data collection
	5	Experience and training	What experience or training did the researcher have?	Method- data collection
Relationship with participants	6	Relationship established	Was a relationship established prior to study commencement?	Method- recruitment
	7	Participant knowledge of the interviewer	What did the participants know about the researcher? e.g. *personal goals, reasons for doing the research*	Method- recruitment
	8	Interviewer characteristics	What characteristics were reported about the interviewer/facilitator? e.g. *Bias, assumptions, reasons and interests in the research topic*	Method- data collection<br>Discussion<br>Funding statement
**Domain 2: study design**				
Theoretical framework	9	Methodological orientation and Theory	What methodological orientation was stated to underpin the study? *e.g. grounded theory, discourse analysis, ethnography, phenomenology, content analysis*	Method- data analysis
Participant selection	10	Sampling	How were participants selected? *e.g. purposive, convenience, consecutive, snowball*	Method- recruitment
	11	Method of approach	How were participants approached? e.g. *face-to-face, telephone, mail, email*	Method- recruitment
	12	Sample size	How many participants were in the study?	Method- recruitment
	13	Non-participation	How many people refused to participate or dropped out? Reasons?	n/a<br><br>Not reported in main manuscript for conciseness.

For peer review only

				Our recruitment method does not allow us to know why participants did not respond to our invitation to participate.
	14.	Setting of data collection	Where was the data collected? e.g. *home, clinic, workplace*	Method- data collection
	15.	Presence of non-participants	Was anyone else present besides the participants and researchers?	Not reported in main manuscript for conciseness.<br><br>Participants were asked to be in a quiet room with no interruptions but we do not know for sure if it was always possible as most interviews were via telephone. Field notes and interview recordings from one face-to-face interview suggest a spouse was present and commenting occasionally.
	16.	Description of sample	What are the important characteristics of the sample? *e.g. demographic data, date*	Method- participants; Table 2
Data collection	17.	Interview guide	Were questions, prompts, guides provided by the authors? Was it pilot tested?	Method- PPI section, data collection; Online Supplementary Materials 1
	18.	Repeat interviews	Were repeat interviews carried out? If yes, how many?	Method- data collection; Table 1.
	19.	Audio/visual recording	Did the research use audio or visual recording to collect the data?	Method- data collection
	20	Field notes	Were field notes made during and/or after the interview or focus group?	Methods- data collection
	21.	Duration	What was the duration of the interviews or focus group?	Methods- data collection
	22.	Data saturation	Was data saturation discussed?	Not reported in main manuscript for conciseness.<br><br>The authors are very cautious about claims of data saturation in thematic analysis (see, for example, arguments in Braun & Clarke, 2021; “To Saturate or not to saturate” Qual Res in Sport, Exercise Health). Data saturation for the current analysis was not aimed for but may have been achieved or approached.<br><br>Recruitment ceased when iterative intervention development was concluded i.e. the research team were satisfied that the interventions were as engaging as possible and a range of different viewpoints from

For peer review only

				patients with different clinical and demographic characteristics had been heard and used.
	23.	Transcripts returned	Were transcripts returned to participants for comment and/or correction?	n/a<br><br>(member checks with participants were not conducted, professional transcribers transcribed the interviews and researchers checked for accuracy)
**Domain 3: analysis and findings**				
Data analysis	24.	Number of data coders	How many data coders coded the data?	Method- data analysis
	25.	Description of the coding tree	Did authors provide a description of the coding tree?	Method- data analysis. A coding tree was not used. We present a description of our process of inductive thematic analysis, including the steps we took in coding the data.
	26.	Derivation of themes	Were themes identified in advance or derived from the data?	Method- data analysis
	27.	Software	What software, if applicable, was used to manage the data?	Method- data analysis
	28.	Participant checking	Did participants provide feedback on the findings?	n/a<br><br>(member checks were not conducted)
Reporting	29.	Quotations presented	Were participant quotations presented to illustrate the themes / findings? Was each quotation identified? e.*g. participant number*	Findings
	30.	Data and findings consistent	Was there consistency between the data presented and the findings?	Findings
	31.	Clarity of major themes	Were major themes clearly presented in the findings?	Findings; Table 3
	32.	Clarity of minor themes	Is there a description of diverse cases or discussion of minor themes?	Findings; Table 3

For peer review only

BMJ Open

Patient perceptions of vulnerability to recurrent respiratory tract infections and prevention strategies; a qualitative study

Journal:	*BMJ Open*
Manuscript ID	bmjopen-2021-055565.R1
Article Type:	Original research
Date Submitted by the Author:	01-Feb-2022
Complete List of Authors:	Dennison, Laura; University of Southampton, Centre for Clinical and Community Applications of Health Psychology, Department of Psychology<br>Williamson, Sian; University of Southampton, Centre for Clinical and Community Applications of Health Psychology, Department of Psychology<br>Greenwell, Kate; University of Southampton, Centre for Clinical and Community Applications of Health Psychology, Department of Psychology<br>Handcock, Molly; University of Southampton, School of Primary Care, Population Sciences and Medical Education, Faculty of Medicine,<br>Bradbury, Katherine; University of Southampton, Centre for Clinical and Community Applications of Health Psychology, Department of Psychology; NIHR Applied Research Collaboration (ARC) Wessex<br>Vennik, Jane; University of Southamption , School of Primary Care, Population Sciences and Medical Education, Faculty of Medicine<br>Yardley, Lucy; University of Southampton, Centre for Clinical and Community Applications of Health Psychology, Department of Psychology; University of Bristol<br>Little, Paul; University of Southampton, School of Primary Care, Population Sciences and Medical Education, Faculty of Medicine<br>Geraghty, Adam; University of Southampton, School of Primary Care, Population Sciences and Medical Education, Faculty of Medicine
**Primary Subject Heading**:	Public health
Secondary Subject Heading:	Qualitative research, Infectious diseases, General practice / Family practice
Keywords:	Respiratory infections < THORACIC MEDICINE, QUALITATIVE RESEARCH, PREVENTIVE MEDICINE, Public health < INFECTIOUS DISEASES, COVID-19, PRIMARY CARE

SCHOLARONE™
Manuscripts

I, the Submitting Author has the right to grant and does grant on behalf of all authors of the Work (as defined in the below author licence), an exclusive licence and/or a non-exclusive licence for contributions from authors who are: i) UK Crown employees; ii) where BMJ has agreed a CC-BY licence shall apply, and/or iii) in accordance with the terms applicable for US Federal Government officers or employees acting as part of their official duties; on a worldwide, perpetual, irrevocable, royalty-free basis to BMJ Publishing Group Ltd ("BMJ") its licensees and where the relevant Journal is co-owned by BMJ to the co-owners of the Journal, to publish the Work in this journal and any other BMJ products and to exploit all rights, as set out in our licence.

The Submitting Author accepts and understands that any supply made under these terms is made by BMJ to the Submitting Author unless you are acting as an employee on behalf of your employer or a postgraduate student of an affiliated institution which is paying any applicable article publishing charge ("APC") for Open Access articles. Where the Submitting Author wishes to make the Work available on an Open Access basis (and intends to pay the relevant APC), the terms of reuse of such Open Access shall be governed by a Creative Commons licence – details of these licences and which Creative Commons licence will apply to this Work are set out in our licence referred to above.

Other than as permitted in any relevant BMJ Author's Self Archiving Policies, I confirm this Work has not been accepted for publication elsewhere, is not being considered for publication elsewhere and does not duplicate material already published. I confirm all authors consent to publication of this Work and authorise the granting of this licence.

For peer review only

Patient perceptions of vulnerability to recurrent respiratory tract infections and prevention strategies; a qualitative study

Laura Dennison[1], Sian Williamson[1], Kate Greenwell[1], Molly Handcock[2], Katherine Bradbury[1,4], Jane Vennik[2], Lucy Yardley[1,3], Paul Little[2] and Adam W A Geraghty[2]

[1]Centre for Clinical and Community Applications of Health Psychology, Faculty of Environmental and Life Sciences, University of Southampton, Southampton, UK; [2]School of Primary Care, Population Sciences and Medical Education, Faculty of Medicine, University of Southampton, Southampton, UK; [3]School of Experimental Psychology, University of Bristol, Bristol, UK. [4]NIHR Applied Research Collaboration (ARC) Wessex, Southampton, UK.

Corresponding Author: Laura Dennison, School of Psychology, University of Southampton, Highfield Campus, Southampton, SO17 1BJ, l.k.dennison@soton.ac.uk

Word count (excluding title page, abstract, references, figures and tables)=5484, with additions as per peer review= 5993

Keywords: Respiratory Tract Infections, Common Cold, Influenza, Prevention, Qualitative Research, Interviews, Thematic Analysis

For peer review only - http://bmjopen.bmj.com/site/about/guidelines.xhtml

ABSTRACT

Objectives: Respiratory Tract Infections (RTIs) are extremely common, usually self-limiting, but responsible for considerable work sickness absence, reduced quality of life, inappropriate antibiotic prescriptions and healthcare costs. Patients who experience recurrent RTIs and those with certain comorbid conditions have higher personal impact and healthcare costs and may be more likely to suffer disease exacerbations, hospitalisation and death. We explored how these patients experience and perceive their RTIs to understand how best to engage them in prevention behaviours.

Design: A qualitative interview study

Setting: Primary Care, UK

Methods: 23 participants who reported recurrent RTIs and/or had relevant comorbid health conditions were interviewed about their experiences of RTIs. Interviews took place as the COVID-19 pandemic began. Data was analysed using inductive thematic analysis.

Findings: Three themes were developed: *Understanding Causes and Vulnerability, Attempting to Prevent RTIs, Uncertainty and Ambivalence about Prevention, along with an overarching theme; Changing Experiences because of COVID-19.* Participants' understandings of their susceptibility to RTIs were multifactorial and included both transmission via others and personal vulnerabilities. They engaged in various approaches to try to prevent infections or alter their progression yet perceived they had limited personal control. The COVID-19 pandemic had improved their understanding of transmission, heightened their concern and motivation to avoid RTIs, and extended their repertoire of protective behaviours.

Conclusions: Patients who experience frequent or severe RTIs are likely to welcome and benefit from advice and support regarding RTI prevention. To engage people effectively, those developing interventions or delivering health services must consider their beliefs and concerns about susceptibility and prevention.

For peer review only - http://bmjopen.bmj.com/site/about/guidelines.xhtml

ARTICLE SUMMARY: STRENGTHS AND LIMITATIONS OF THIS STUDY

- This study contributes to a limited evidence-base on the experiences of people who suffer recurrent RTIs and/or are at risk of complications, morbidity and mortality as a consequence of RTIs.
- The interview technique and inductive thematic analysis allowed an in-depth exploration of beliefs and experiences, prioritising the participants' perspectives.
- The interview timing coincided with the beginning of the COVID-19 pandemic, allowing interesting observations of how experiencing the pandemic and its restrictions affected people's understandings and behaviour relating to non-pandemic RTIs.
- The purposive sampling strategy ensured that participants had a range of clinical and demographic characteristics but may have failed to capture underserved populations; particularly minority ethnic groups and people with high levels of deprivation.

For peer review only

INTRODUCTION

Respiratory tract infections (RTIs) are infectious diseases of the upper or lower respiratory tract that are usually caused by viruses. RTIs include the common cold, influenza (flu), pharyngitis, tonsillitis, sinusitis and bronchitis along with pandemic infections such as COVID-19. RTIs are common with most adults experiencing at least one cold per year[1]. RTIs typically produce mild to moderate symptoms and are usually self-limiting. Nonetheless they are a leading cause of work sickness absence[2] and reduce health-related quality of life, affecting physical, mental, and social functioning[3]. They are also responsible for substantial healthcare costs, mostly in primary care[4 5]. Consultations for RTIs can also result in unnecessary antibiotics prescribing, contributing to antimicrobial resistance[6 7]. Certain subgroups of patients have worse health outcomes and incur higher healthcare costs from RTIs. This includes patients who experience frequent or recurrent infections and those whose older age and/or comorbid conditions put them at higher risk of disease exacerbations, hospitalisation and death[8 9]. RTI prevention interventions, particularly those focussed on these recurrent and at-risk patients, would benefit individuals and healthcare systems alike.

A wide range of medical and behavioural RTI prevention approaches have been investigated including; hand hygiene[10-12], vaccination[13-15], social distancing[10 16], mask-wearing[10 11 17], vitamins and supplements,[18 19] gargling, nasal rinses and nasal sprays[20], exercise[21 22], and mindfulness meditation[21]. Interest and urgency around RTI prevention has been prompted by the COVID-19 pandemic. However, in addition to efficacy, prevention interventions must be acceptable to the target population; they must feel personally relevant, feasible and make sense in the context of people's experiences. Therefore, gaining insight into how people understand and experience RTIs and RTI prevention is a vital step in the development and implementation of successful prevention interventions[23].

Many studies have investigated lay perspectives on RTIs, using either qualitative designs or survey methods. Insights include that colds and flu are considered familiar and trivial[24-26], with people often considering themselves to be at low risk of both contagion and severe outcomes compared to others[25 27-29]. Transmission from

For peer review only - http://bmjopen.bmj.com/site/about/guidelines.xhtml

symptomatic people is often correctly recognised as important[24 26 29 30]. Confusion may exist about the importance of viruses versus bacteria and the relevance of temperature, damp and pollution[25 30-35]. RTI severity seems to be judged based on symptom duration, discomfort and impact on activities[25 36 37]. People describe attempting to manage RTIs at home[25 32 36 37]. Seeking a GP consultation tends to be triggered by long symptom duration, specific symptoms[25 38], or where symptom resolution in earlier illness episodes has been attributed to antibiotics[36 37]. Considerable fatalism exists about catching RTIs[24 26 39 40]. Furthermore, whilst people may be familiar with and adopt prevention approaches such as avoiding infectious people and hand-washing[35 41]. these behaviours can be considered unacceptable and/or unattainable[27 29 40]. People tend to emphasise their own good health, strong personal reserves, and healthy lifestyle as providing defence against RTIs[24 27 30 41]. Experiencing RTIs may even be considered beneficial: a natural, necessary strengthening of the immune system[24 26].

There are some important limitations to the scope of existing research. Firstly, most studies sample the general public, rather than patients with higher infection frequency, morbidity or mortality. Secondly, most studies focus specifically on colds and flu rather than the wider range of RTIs. Furthermore, this literature is dominated by understanding patient perspectives of RTIs specifically in the context of reducing antibiotic prescribing and increasing flu vaccine uptake.

The current paper extends the literature by focusing on patients who get recurrent RTIs and/or are at risk for having more severe consequences of RTIs. We explored how these patients experienced and understood their infections in order to understand how best to engage them in prevention behaviours. This research was planned prior to the COVID-19 pandemic and was nested within a NIHR programme grant developing and evaluating RTI prevention approaches. This work was originally driven by the impact of recurrent *non-pandemic* RTIs on patients and healthcare systems. The pandemic, and the resulting requirement for both 'at risk' patients and whole populations to adopt and adhere to an array of RTI prevention behaviours, highlights and accelerates the need to understand people's experiences of and beliefs about RTIs, their vulnerability, and infection prevention behaviours.

For peer review only - http://bmjopen.bmj.com/site/about/guidelines.xhtml

METHOD

We report this qualitative interview study with reference to the COREQ checklist (Supplementary Material 1).

Ethics Approval

Ethics and research governance approvals were granted by NHS and the University of Southampton (REC/HRA19/SC/0354; ERGO:48223). Participants provided written or online consent after reading the participant information sheet and having the opportunity to discuss participation with the research team. They were free to withdraw at any time. Procedures were in place to pause or discontinue interviews should participants become distressed however this was not necessary. Participants were assured of confidentiality and quotations from their interviews have been anonymised. Participants were debriefed and received a £10 retail voucher to thank them for their time.

Patient and Public Involvement (PPI)

A panel of five PPI contributors have supported the study planning and conduct, some from the grant application stage. The PPI panel includes people with experiences of recurrent infections and/or long-term health conditions that make them vulnerable to more severe RTIs. Contributions included editing and improving our participant information sheets, consent forms and interview schedules. They also participated in pilot interviews to ensure minimal participant burden and improve the question coverage, wording and process.

Recruitment

We recruited adults (18+) who had experienced recurrent RTIs (operationalised as ≥3 in the last year and/or ≥1/year over the last 3 years). Because RTIs are common and usually self-limiting, they are not routinely recorded in medical records (only when patients consult). Therefore, a combination of medical record searching and self-report was used to identify participants. Three Hampshire (UK) GP practices identified possible participants. The practices were chosen to capture a mixture of urban and semi-rural locations and varying deprivation levels. Practices searched their lists and posted invitations and information sheets to a) patients who consulted for ≥1 RTI within the last year and received an antibiotics prescription and b) a sub-group of patients who also had asthma, COPD or chronic sinusitis. Interested

For peer review only - http://bmjopen.bmj.com/site/about/guidelines.xhtml

patients returned reply slips, on which they self-reported their recent RTI history. We then purposively sampled from the 52 interested patients to achieve a heterogeneous sample based on frequency of self-reported RTIs, health conditions, age and gender. Twenty-three participants were interviewed. Data collection and analysis were conducted in parallel. Recruitment ceased when patients with a range of clinical and demographic characteristics had been sampled and sufficient data was available to support both the thematic analysis of RTI experiences and beliefs and the iterative intervention refinement (see below).

Data collection

Data collection took place between March and August 2020, coinciding with the beginning of the COVID-19 pandemic. Three participants were interviewed face-to-face in their homes or University premises in early March. The rest participated by telephone during the first UK lockdown or as national restrictions eased. After gathering brief demographic and health data participants were interviewed following a schedule consisting of two sections (see Online Supplementary Material 2).

Section 1 involved an in-depth exploration of their personal experiences of having RTIs, including the types of infections they experience, how infections affect them, when they experience infections, why they believe they experience them, how they manage them and if and how they attempt to avoid them. Later interviews also included questions on the impact of COVID-19 and the pandemic restrictions on their experiences of RTIs. Questions were phrased openly and non-directive prompts were used to elicit extensive discussion.

Section 2 gathered data on participants views and experiences of web-based RTI prevention interventions that were under development. Participants used and discussed either an intervention promoting nasal spray use (n=13) or physical activity and stress management interventions (n=10). Some of the interviews were think-aloud style (n=14), others were post-intervention use interviews (n=7). 2 participants engaged in both types of interview, over two different sessions. Section 2 data collection was primarily intended to be used for a iterative intervention optimisation, as part of a wider project (reported elsewhere) where the data was used to ensure that the interventions were engaging, persuasive and relevant[23].

For peer review only - http://bmjopen.bmj.com/site/about/guidelines.xhtml

However, as participants viewed and discussed the interventions they tended to elaborate on their personal RTI experiences and beliefs. Therefore, any section 2 data relating to current study research questions was included in the current analysis.

SW and LD conducted the interviews (interviewing 20 and 3 participants respectively). Both interviewers were female academics, with qualitative interviewing and health psychology expertise. Interviews ranged from 41 to 104 minutes (mean=70.5), including both section 1 and 2 . Interviewers took field notes. Interviews were audio-recorded then transcribed verbatim.

Data Analysis

An inductive thematic analysis was conducted from a critical realist perspective[42]. The analysts have health psychology (LD, KG, SW, AG), medicine or healthcare (MH, JV) research backgrounds and conducted the analysis as a study nested within a programme of research relating to behavioural approaches to RTI prevention. LD conducted the analysis, periodically presenting and discussing preliminary codes and themes and with co-authors SW, KG, JV, AG and MH. This practice allowed multiple perspectives to be considered, avoided idiosyncratic interpretations and helped to highlight modifications or clarifications to improve the analysis. The authors do not consider an unbiased or objective qualitative analysis as achievable and instead aim to present descriptive and interpretative accounts of patterns in the data that have been derived from a rigorous engagement with the data using analytic procedures that stay close to the original data in order to derive inductive themes.

To begin the analysis, familiarisation with the data was achieved through listening to audio-recordings and reading transcripts. Next, thorough line-by-line coding began, facilitated by NVivo 12 software. Descriptive labels (codes) were attached to words or phrases that captured ideas relating to the research questions. Codes were reviewed frequently, and definitions were developed and iteratively edited. The analysis proceeded to examine the codes and develop them into themes which captured patterns and features of the data. This involved an iterative process of clustering together or merging similar codes and splicing or dividing codes, all the

For peer review only - http://bmjopen.bmj.com/site/about/guidelines.xhtml

time engaging in constant comparison between codes and transcripts and searching for deviant cases. Themes were iteratively reviewed, refined, organised and relabelled, with the input of the wider team, to ensure the final set of themes and subthemes were insightful and coherent.

FINDINGS

Participants

Table 1 shows participant characteristics. Participants self-reported a mean of 3 infections in the previous year. Ten participants had asthma, three had COPD, and three had chronic sinusitis. More females than males participated. Age ranged between 18 and 83.

Table 1: Demographic and clinical characteristics of participants (n=23)

	Mean (SD), range or N (%)
**Age (years)**	53.87 (19.62)<br>range 18-83
**Gender**	
Male	6 (26.09%)
Female	17 (73.91%)
**Marital status**	
Married or living with partner	11 (47.83%)
Single	5 (21.74%)
Divorced	4 (17.39%)
Widowed	3 (13.04%)
**Employment status**	
In paid work (full or part time including self-employed)	10 (43.48%)
Retired	6 (26.09%)
Not working because of illness/disability	2 (8.70%)
Other (unemployed, homemakers, students, volunteers)	5 (21.74%)
**Education (age left education)**	
16 or before	3 (13.04%)
17 or 18	6 (26.09%)
Over 18	14 (60.87%)
**Ethnicity**	
White British or White Irish	16
Mixed White/Asian	1
No data	6
**Deprivation (2019 Index of Multiple Deprivation Decile[1])**	Mdn= 9 (IQR 6.5)<br>range 2-10

For peer review only - http://bmjopen.bmj.com/site/about/guidelines.xhtml

**Health Conditions**[2]<br>Asthma<br>COPD<br>Chronic Sinusitis<br>None of these conditions	 10 (43.48%)<br>3 (13.04%)<br>3 (13.04%)<br>12 (52.17%)
**Number of RTIs in last 12 months (self-report)**	3.23 (1.6)<br>range 1-6
**Number of RTIs per year in last 3 years (self-report)**<br>≥1<br>≥3	 21 (91.3%)<br>15 (65.2%)
**Types of RTIs experienced at least once in last 12 months (self-report)**<br>Cold<br>Flu<br>Throat infection<br>Chest infection<br>Sinus infection<br>Ear infection	 <br>17 (73.91%)<br>4 (17.39%)<br>13 (56.52%)<br>12 (52.17%)<br>10 (43.48%)<br>4 (17.39%)
**Concern about RTIs** (0-10)[3]	5.36 (3.09), 0-10
**Consequences of RTIs** (0-10)[3]	6.04 (3.08), 1-10

[1] (1=most deprived, 10 is least deprived), data available for 17/23 participants; [2] 4 participants had more than one of these health conditions; [3] Brief illness Perception Questionnaire (B-IPQ) concern and consequences items, higher scores indicate higher concern and worse perceived impact.

For peer review

Themes and subthemes

Three themes and 12 subthemes were developed from the data (Table 2). A further, overarching theme which influenced all other themes is described. This final theme relates to the evolving nature of RTI experiences, beliefs and behaviours given the emerging pandemic context. The themes are explained below, alongside illustrative quotations. Unless described, no clear patterns in findings relating to demographic characteristics were discerned.

For peer review only - http://bmjopen.bmj.com/site/about/guidelines.xhtml

Table 2: Themes and Subthemes

	Themes	Subthemes
**Overarching Theme:** Changing experiences because of COVID-19.	Understanding causes and vulnerability	Catching RTIs from other people<br>Something in the environment<br>Defective bodies and inadequate defences<br>Wondering why
	Attempting to prevent RTIs	Trying (desperately) not to get RTIs<br>Hygiene measures<br>Avoiding symptomatic people<br>Keeping yourself strong and healthy<br>Nipping infections in the bud
	Uncertainty & ambivalence about prevention	Inevitability and limited control<br>Judging whether things help<br>Not letting it dominate

Understanding causes and vulnerability

Participants had complex, multidimensional understandings about why RTIs occur and how and why they are more susceptible than others.

Catching RTIs from other people

All participants understood that RTIs were transmitted between people, identifying people and situations that they considered pertinent for their own infections. Some discussions included the relevance of symptomatic people, highlighting coughs and sneezes as transmission mechanisms and occasionally drawing on the concepts of germs and viruses.

> If you're next to somebody who's coughing and spluttering, the chances are you'll end up with the same. (P21, female, 60s).

Participants emphasised proximity to others as important for spreading infection and physical contact as particularly risky.

> [On the Underground] you're like sardines in a can and if there's anything going you're going to catch it (P9, male, 80s)

> Where people are hugging and kissing. That's when these things get passed around. (P23, female, 53s)

For peer review only - http://bmjopen.bmj.com/site/about/guidelines.xhtml

For peer review only

A few participants also described getting infections from contaminated objects and surfaces.

Many participants emphasised children as frequent and significant sources of infection.

> Little germ machines (P23, female, 50s)

> Super-spreaders (P4, female, 60s).

Children were described as having frequent infection and being likely to pass infections on to their families. Their poor respiratory hygiene was highlighted.

> Particularly revolting and snotty (P21, female, 60s)

Children posed particular problems for older participants who were wary of catching RTIs.

> [My grandchildren] had a cold and I didn't say anything because they are glad to come down to see us, but I was thinking they were sat on that [chair] and I was thinking 'please don't get any closer'. (P1, male, 60s)

Several participants highlighted their current or past occupation as incurring high RTI risk. They described exposure to large numbers or certain types of people (e.g. children, students, general public), or close contact (e.g. a tattoo artist), as well as commuting on public transport.

Something in the environment

Many participants referred to environmental influences as responsible for their RTIs. Season was referenced as a very obvious factor by many although a few described having year-round infections.

> Most of the winter months, I am like, 'Oh, I've got a cold again.' (P2, female, 30s)

A few considered exposure to cold air and/or temperature *change* as causing infections and/or provoking symptoms.

> Maybe when I go out and I don't keep warm enough, so my chest is exposed (P14, female, <20s)

For peer review only - http://bmjopen.bmj.com/site/about/guidelines.xhtml

> Going out into the cold, coming back to the heat, and I think that's just where people get colds from. (P8, female, 50s)

A few participants described mixing more with people indoors as relevant to seasonality and, consistent with their understandings of transmission via people, participants sometimes referred to breathing virus-contaminated air. A few participants perceived pollution, damp or sensitivity to triggers in the atmosphere (e.g. deodorants, cleaning products) as potential causes of their RTIs. Those who emphasised poor air quality and environmental triggers tended to also discuss having asthma and/or allergies.

Defective bodies and inadequate defences

In addition to the causal factors mentioned above, most participants perceived that deficiencies within their bodies had created vulnerabilities to RTIs. Many participants discussed picking up infections more readily than others, experiencing them more severely and finding them harder to shift.

> I just seem to pick up everything. (P4, female, 60s)

> [My husband] doesn't tend to suffer as badly as I do. We joke about it that I make more fuss, but it isn't that. Mine just goes on for longer and is always worse. (P12, female, 70s).

Participants described how ageing and age-related lifestyle influenced RTI vulnerability. A few of the older participants spoke of their advanced age as weakening their ability to fight off infections.

> When you're older you're just not as strong or it's not as easy to shrug these things off. (P1, male, 60s)

However, some older people found that ageing-related lifestyle changes and retirement had reduced exposure to people, making them *less* susceptible to picking them up.

Several participants felt their chronic health conditions, or a previous severe RTI meant their body did not fight off RTIs effectively, describing weakened defences or immune systems.

For peer review only - http://bmjopen.bmj.com/site/about/guidelines.xhtml

> It's just a little bit weaker and just that little kind of chink in its armour is enough to let infections in whereas previously [before asthma] I was a bit healthy and robust and the infection would be fought off instantly by my own immune system. Now, it's just a little bit tired and it just can't cope quite as well. (P18, female, 50s)

> My immune system is rock bottom at the moment where I've just come out the other side of [a lengthy recovery from a severe RTI] so that makes me, in my head, more vulnerable. I think I could easily pick up something else. (P15, female, 50s)

Some discussed how past medical treatments, may have left them with weakened defences. P4 (female, 60s) described radical sinus surgery over 30 years ago that had left her with "no natural defences at all" and P12 (female, 70s) suspected that her treatment for a thyroid condition had affected her immune system.

Some participants believed a part of their body was weakened, malfunctioning, or malformed.

> A slight weakness on the throat (P3, male, 70s),

> There's something, somewhere in me, that decides it's going to not work properly (P7, female, 80s),

> Something wonky in my nose (P17, female, 40s).

Participants also discussed the importance of stress. Many participants believed that experiencing upsetting, difficult experiences made *people in general* more likely to suffer RTIs (and other illnesses) or make your RTI more severe. A few participants considered positive mental health and happiness to be protective, providing a better ability to fight infections. Many also considered stress pertinent to their personal experience of recurrent or severe RTIs.

> That's why I succumbed to being so unwell because of the stress that I was under. I think that was a massive, played a massive part. (P15, female, 50s)

A few participants felt that vulnerability tended to occur *after* stress or busyness, and discussed a perception of succumbing at the point of beginning to relax or take a break. In contrast, some participants did not perceive a role for stress in getting

For peer review only - http://bmjopen.bmj.com/site/about/guidelines.xhtml

infections. Some emphasised an absence of stress (particularly older participants), citing a lack of life stressors or a lack of personal stress reactivity (“I'm not that sort of person”, P17, female, 40s). Some of these participants, however, discussed busyness and exhaustion as personally pertinent; emphasising being ‘run down’ rather than ‘stressed’.

Wondering why

Despite offering the explanations of personal vulnerability and describing an understanding of viral transmission outlined above, some participants simultaneously referred to *not* fully understanding why they suffered regularly or severely with RTIs. Some expressed a residual, enduring sense of mystery surrounding their RTIs and a tendency to review and analyse their own illness episodes to try to understand and explain their experiences. The unknowns sometimes related to being unable to trace the infection’s origin, for instance where participants could not identify how or where they had been exposed to RTIs. Some participants struggled with explaining why their own infections were worse than those of people they knew who had seemingly caught the same infection.

> There seems to be no rhyme or reason to it because nobody else seems to be ill around me, it just seems to be me. (P7, female, 80s)

Importantly, claims to not understand why they suffered RTIs appeared not only to reflect genuine confusion and uncertainty. They could also function as a conversational device to communicate a sense of injustice; that their infections are undeserved. Unfairness was keenly felt, given their cautious behaviour and attempts to look after their health.

> *[Interviewer: Why do you think you get these infections?]* Participant: That's something I ask myself many, many times. I've got friends who are older than me and they don't suffer like I do. I've got friends who are younger than me. They go out drinking. They smoke. They also indulge in other things. They don't suffer like me. (P22, female, 70s)

Attempting to prevent RTIs

For peer review only - http://bmjopen.bmj.com/site/about/guidelines.xhtml

Almost all participants wanted to prevent RTIs and were taking steps to try to protect themselves.

Trying (desperately) not to get RTIs

Some participants' attempts to avoid getting RTIs involved multiple, complex behaviours and regimes.

> When you know that you have a tendency to be poorly when you catch things, you just do everything you can to not catch them. (P21, female, 60s).

Many RTI prevention strategies were long-term habits, often learned in childhood (e.g. hand and respiratory hygiene). These behaviours were considered obvious and given little thought.

> It's just been second nature" (P15, female, 50s).

Participants also described their repertoire of RTI prevention behaviours as evolving over time, with new products and strategies adopted following recommendations from family, friends, and healthcare professionals. Participants were open to and enthusiastic about trying prevention approaches

> You can get really fed up of getting ill and you can be like desperate to latch on to anything. (P13, female, 20s)

Hygiene measures

Most participants described hygiene measures to prevent germs getting into their system. Handwashing was almost universally reported, usually as something obvious.

> All the normal hygiene, hand-washing that sort of thing (P17, female, 40s).

Some mentioned handwashing very briefly, others described specifics of their meticulous regimes. A few described other hygiene measures including cleaning objects and safe tissue disposal.

> If someone comes in and they say they're feeling a bit run down then I'll make sure that I've sterilised and cleaned the room and my hands properly. (P5, female, 20s)

For peer review only - http://bmjopen.bmj.com/site/about/guidelines.xhtml

Mask wearing and ventilation were discussed infrequently. A few people mentioned avoiding touching their face as important, but difficult. In general, participants described their exemplary hygiene behaviour but highlighted poor practices of others.

> They've sneezed, and I felt it on the back of my head. (P1, male, 60s)

> I wish more people did it [handwashing]. (P23, female, 50s)

Avoiding symptomatic people

A key prevention strategy our participants adopted was avoiding symptomatic people. Most described deliberately avoiding anyone displaying or declaring cold symptoms.

> I will take a very wide berth (P10, female, 60s)

> I'm just, 'Stay away from me'. (P1, male, 60s)

Some were extremely vigilant about avoiding ill people

> Everybody knew not to come and visit if they had so much as a sniffle (P23, female, 50s).

However, other participants described how this is impossible or undesirable (see below *Not Letting it Dominate*).

Keeping yourself strong and healthy

Many participants described undertaking health-enhancing behaviours such as eating healthily, taking vitamins, keeping physically fit, nurturing psychological wellbeing, not smoking, alcohol avoidance and staying hydrated. However, these behaviours were rarely adopted or sustained with preventing RTIs as the end goal. Instead they were long-term habits or preferences or related to healthy ageing or managing other health conditions

> That's what I've always done. That is who I am (P4, female, 60s),

> It's good life practice (P6, male, 70s),

Nonetheless, participants believed or hoped that these healthy behaviours would provide some protection from RTIs.

For peer review only

For peer review only - http://bmjopen.bmj.com/site/about/guidelines.xhtml

> I would hope that it means that I'm a bit more resilient to fighting them off. (P21, female, 60s)

Unlike the behaviours mentioned above, a few behaviours were adopted specifically for preventing RTIs. Several participants discussed the flu vaccination (with mixed uptake and views on efficacy and safety). A few ensured they kept warm in cold weather. Several participants with asthma or COPD described ‘keeping on top of’ their medications, monitoring indicators of health conditions (e.g. peak flow) or having a medication escalation regime in place.

> It just keeps your body at an even keel, don't it? Because if you let your medication drop, then your body immune system will drop as well. (P8, female, 50s)

Nipping infections in the bud

Besides attempts to *prevent* infections, many participants discussed taking prompt action when RTI symptoms began and attempting to alter the infection’s course.

> When I know [a] cold's coming [..] I try and stop it going any further (P17, female, 40s)

> [if] I don't deal with a suspected cold quickly, it will take hold. (P10, female, 60s)

Through experience, participants recognised early infection symptoms and subsequently tried to ‘nip infections in the bud’, stopping symptoms progressing and worsening. Some found this was at least sometimes effective. The behaviours considered helpful varied and included early use of over-the-counter cold and flu remedies, extra vitamins or supplements, gargling, nasal rinses and nasal sprays, or swift GP consultation for antibiotics.

> With a sore throat, sometimes I just go straight home and gargle […] I get the tickle, get the thing, it goes away in a couple of days. (P3, male, 70s)

> Obviously, I try and get some antibiotics. If I can get them early, I can stop it getting hold. If it gets hold, there's nothing I can do. (P22, female, 70s)

Participants with asthma and COPD discussed altering medications in response to suspected beginnings of infections.

For peer review only - http://bmjopen.bmj.com/site/about/guidelines.xhtml

> I do "up" [increase] my steroid inhaler, so that kind of gives my lungs a boost before the illness strikes and then can usually fend things off that way, but if I don't feel it coming on, then I have to fight it once it arrives, which is slightly harder. (P18, female, 50s)

Uncertainty and ambivalence about prevention

Most participants discussed the extent to which RTI prevention seems possible or desirable.

Inevitability and limited control

Despite their concerted prevention efforts, most participants described feeling somewhat powerless over contracting infections, with some discussing the inevitability of symptoms worsening and progressing.

> You can do all these things to try and prevent but if you're going to get something you're going to get it I think! (P15, female, 50s)

> I've done everything I possibly can to try and prevent it happening and not making it as bad as what it is, but it just never seems to work, and I always end up at the doctor's with steroids, antibiotics. (P11, female, <20)

Central to the perceived inevitability was the understanding that RTIs come from other people. Problems arose because is not always obvious who has infections nor is it always possible or desirable to avoid people. Participants often described feeling at the mercy of other people's behaviour and hygiene.

> I've got all my systems are all in place to avoid all those things. It's only really when I step out anywhere else that I'm in an environment, that I can't control (P23, female, 50s)

> I can't control everybody else, the snotty noses and the likes.(P2, female, 30s)

Judging whether things help

As discussed in the previous themes, sometimes participants had experienced perceptible reductions in RTIs as a result of nipping infections in the bud or avoiding

For peer review only

people (see previous theme). However, more often participants described uncertainty about whether the strategies they adopted were helpful. Some seemed indifferent to scrutinising helpfulness, lacking curiosity about habitual behaviours. Some spoke of the difficulties of evaluating their effectiveness.

> Whether they do any good or not I have no idea, because I feel exactly the same as I did before (P13, female, 20s)

> I suppose I'd have to not take them [vitamins] for a year and then try and see the difference (P10, female, 60s).

Sometimes participants abandoned strategies that did not produce obvious benefits but sometimes they persevered through habit or hope.

> I'm assuming they're doing something! (P19, female, 40s)

> I'm going to continue doing it because it might make a difference soon, or one day. (P11, female, <20)

Not letting it dominate

Participants often discussed how a balance between RTI prevention and quality of life must be achieved. Some RTI avoidance behaviours were considered undesirable or almost impossible, particularly those that involved social distancing from even asymptomatic people.

> You can't put yourself into a bubble and do nothing. That would be a very destructive way of living. Then, you become victimised by being unwell which is no good to anybody. (P4, female, 60s)

> There's the trade-off isn't there? With quality of life. (P23, female, 50s)

Whilst some felt so vulnerable that they considered it essential to be ‘hypervigilant’, ‘so careful’ and ‘a little bit oversensitive’ (P23, female, 50s), several participants expressed wariness of becoming, or being perceived as, a hypochondriac.

> There's a fine line between taking care and allowing your concerns to impact on your life. (P21, female, 60s)

Changing experiences because of COVID-19.

For peer review only - http://bmjopen.bmj.com/site/about/guidelines.xhtml

For peer review only

Participants' perceptions of RTIs and prevention behaviours were influenced by the context of the COVID-19 pandemic. Participants recognised COVID-19 as an RTI, albeit one that is unfamiliar and particularly severe, creating heightened motivation for RTI prevention.

> Particularly now, you need to be armed to the teeth with everything you can to protect yourself! (P23, female, 50s)

Several participants were fearful, perceiving themselves to be at risk of severe COVID-19 due to the same vulnerabilities that they understood predisposed them to other RTIs.

> I'm paranoid. Absolutely paranoid about getting it because I can't breathe very well anyway (P15, female, 50s)

Participants recognised similarities in transmission mechanisms between COVID-19 and non-pandemic RTIs and described developing better understandings about spread of infections and increasing insight into how to stop transmission, particularly hygiene measures.

> Before this, I wasn't so aware of how illnesses travel, especially a cough and a sneeze [..] and how long it stays on the surface. (P14, female, <20)

> We're all used to using these hand sanitisers and we understand how they're protecting us. (P23, female, 50s)

> I've definitely learned through coronavirus that I touch my face all the time. (P10, female, 60s)

However, their sense that their health was at the mercy of other people appeared heightened; participants were extremely conscious and critical of other people's behaviour.

> They're mixing with people and then still going out and about the shops like their normal day-to-day living, and that's the scary bit. They don't understand that they might not have it, but they might be a carrier, and carry it to people like me. (P8, female, 50s)

During national lockdown, shielding and other restrictions, participants' usual behaviour of distancing themselves from *symptomatic* people changed to distancing

For peer review only - http://bmjopen.bmj.com/site/about/guidelines.xhtml

from almost everyone. Consequently, participants experienced an unequivocally successful RTI prevention method, often for the first time.

> Where you're not mixing with people, you're not catching colds! (P21, female, 60s)

Some participants found social distancing and isolation burdensome

> I'm questioning where that trade-off is comfortable. (P23, female, 50s)

Nonetheless some had discovered approaches that they speculated adopting in the longer term for 'normal' RTIs.

> Being maybe more cautious in social interactions, to prevent catching things anyway. Maybe we'll all be like that! (P20, male, 50s)

DISCUSSION

Novel contributions and implications

This study extends the literature by exploring how a recurrent and at-risk patient group experiences and understands their RTIs, including if and how they can be prevented. Some findings echoed those from previous research but several important new insights emerged. These may be useful for determining how to successfully engage these patients in RTI prevention behaviours and are therefore discussed in these terms below.

Like previous studies on colds and flu[24-26 29 30], our participants demonstrated a largely accurate understanding of RTI transmission. Their identification of children as important infectors (for non-pandemic RTIs) came out particularly strongly in this study, compared to past research. Alongside their correct understandings of virus transmission, many participants held concurrent beliefs about the causal role of temperature, damp, allergens and pollution. Such beliefs have been noted previously, but sometimes considered erroneous, folklore, or culture-specific.[31] However, these factors could be relevant; modest evidence suggests that cold and damp could be causally linked to RTIs[43 44]. Furthermore, it is widely accepted that respiratory viruses thrive in cold and damp environments. Another explanation is that patients may confuse a causal role of these factors with their tendency to trigger or exacerbate respiratory symptoms from their chronic conditions (e.g. coughs and

For peer review only - http://bmjopen.bmj.com/site/about/guidelines.xhtml

wheezes from asthma or allergies). Addressing causal beliefs may be necessary if an intervention relies on accurate understandings of virus transmission.

Unlike some earlier studies[27 29] our patients were highly motivated to prevent RTIs and engaged in many relevant behaviours. Consistent with their grasp of transmission mechanisms, they focused (even pre-COVID-19) on avoiding contact with symptomatic others and undertaking hygiene measures. Previous literature, in contrast, suggested such measures might be considered unfeasible, unnecessary or unacceptable[27 29 41]. Our participants were open to prevention recommendations and described extending their prevention repertoire over time. Furthermore, some were considering continuing with COVID-19 measures such as social distancing and mask-wearing to avoid routine RTIs in the future. It therefore seems these recurrent and at-risk patients will be highly receptive to additional support with infection prevention. The differences in motivation and acceptance of prevention behaviours between the current study and previous research likely reflects our sampling of a higher risk/higher impact patient group but may also reflect the heightened concern due to the COVID-19 context. Contrary to previous research where RTIs have often been described as trivial and participants have not considered themselves susceptible to catching RTIs or vulnerable to severe consequences[24 26 27 29] most of our participants perceived a personal vulnerability to frequent and/or severe infections. Some described how RTIs were linked to medical conditions, stress was salient for others, some had vague ideas about weak immune systems, and other showed uncertainty and frustration about vulnerability. Interventions that offer an acceptable explanatory framework for recurrent infections, help people understand their personal vulnerability, and provide a corresponding, coherent prevention strategy may prove validating and helpful to these patients.

As in some previous studies[40 41], some participants described engaging in behaviours to stay healthy and strong. However, our analysis highlighted how most of these efforts appeared to be engaged in through enjoyment, habit or in the service of managing other health conditions. Few participants appeared to have consciously considered and committed to boosting their immune system to prevent RTIs, though, for example, increased physical activity, adequate sleep and stress reduction. No participants mentioned recommendations from health professionals to make lifestyle changes for RTI prevention. Patients suffering from recurrent or severe RTIs should

For peer review only - http://bmjopen.bmj.com/site/about/guidelines.xhtml

be given information about how these behaviours relate to immune health. They may benefit from support and encouragement with initiating, stepping up, and maintaining these behaviours.

Despite seeking to avoid RTIs, participants had some doubts about whether prevention is indeed possible or desirable and were unclear about the extent to which their strategies were helping them. Fatalism about RTIs has been identified previously[24 26 41] but, importantly, here participants describe helplessness despite considerable prevention efforts; the idea of being at the mercy of other people's risky and inconsiderate behaviour is apparent as is a sense of unfairness. Interventions will need to improve outcome expectancies by convincing people that prevention approaches will be effective, and by helping people to identify situations and behaviours where they can exert personal control, despite the role of other people in infection transmission. A novel finding was that many participants believed that they sometimes succeeded in stopping RTIs developing or worsening through rapid intervention when they begin. These anecdotes suggest people perceive their own actions sometimes alter the progression of RTIs. Drawing on these experiences might help people to develop confidence about other approaches to preventing infections. Finally, participants expressed concerns about prevention behaviours dominating life. Concerns about prevention strategies may require more detailed research, including worries or barriers specific to particular approaches. Acknowledging and addressing concerns and careful communication and framing may be necessary to make prevention interventions acceptable.

Strengths and limitations

Whilst our recruitment strategy successfully identified recurrent and at risk RTI patients, the sample was self-selecting and nesting our data collection within an RTI prevention intervention development study may have attracted participants who were particularly interested in prevention. This may limit the transferability of findings. Furthermore, we are unlikely to have included the experiences of underserved populations. Although we recruited from practices serving a variety of communities and included 6 participants with IMD deciles <4, the sample was skewed towards low deprivation. Of those that provided ethnicity data, all but one were White British/Irish. This is a limitation, especially given the increasing awareness of the unequal

For peer review only - http://bmjopen.bmj.com/site/about/guidelines.xhtml

susceptibility to and impact of COVID-19[45-48]. Capturing the perspectives of underserved populations who are also unequally burdened by the impact of RTIs remains a priority.

Conclusion

Our participants described multifaceted causal understandings of their recurrent and/or severe RTIs, including both transmission from others and their own perceived weakened defences. Despite trying various strategies to prevent infections, RTIs persisted and they often described feeling powerless to influence infections. People with a history of recurrent RTIs seem likely to welcome prevention interventions but may need to be persuaded that approaches will be effective, drawing on a convincing and personally meaningful rationale for how interventions will help them. Our study shows how COVID-19 has facilitated learning about RTI spread, heightened feelings of vulnerability but also exposed participants to highly effective prevention strategies. The current heightened concern and awareness about infections may be fertile ground for introducing prevention approaches for non-pandemic infections, at least in populations who already perceive themselves to be badly affected or vulnerable. Future research should explore how people respond to interventions to help them reduce RTI frequency and severity.

Contributorship statement. Authors AG, LY and PL conceived the study idea and initial study design with later input in planning the study from KB, LD, KG, SW and JV. SW and LD collected the data. LD led data analysis with analytic and interpretive input from SW,KG,MH,KB,JV,LY and AG at different stages. LD drafted the manuscript and SW,KG,MH,KB,JV,LY,PL and AG contributed to critically editing and approving the final manuscript.

Acknowledgement statement: Kate Martinson manged ethical approvals and recruitment. Thank you also to our public contributors Hazel Patel, Debs Smith and Samantha Richards-Hall.

Competing interests: none declared.

For peer review only - http://bmjopen.bmj.com/site/about/guidelines.xhtml

Funding: This article presents independent research funded by the National Institute for Health Research (NIHR) under its Programme Grants for Applied Research (PGfAR) Programme (Grant Reference Number RP-PG-0218-20005). The views expressed are those of the authors and not necessarily those of the NIHR or the Department of Health and Social Care. Lucy Yardley is an NIHR Senior Investigator and her research programme is partly supported by NIHR Applied Research Collaboration (ARC)-West, NIHR Health Protection Research Unit (HPRU) for Behavioural Science and Evaluation, and the NIHR Southampton Biomedical Research Centre (BRC).

Data Availability Statement

Data are available upon reasonable request. The data that support the findings of this study are available from the corresponding author upon reasonable request.

Online Supplementary Materials

1. COREQ Checklist
2. Interview schedule

REFERENCES

1. Heikkinen T, Järvinen A. The common cold. *Lancet* 2003;361(9351):51-59. doi: 10.1016/s0140-6736(03)12162-9
2. ONS. Sickness absence in the UK labour market: 2020 2021 [Available from: https://www.ons.gov.uk/employmentandlabourmarket/peopleinwork/labourproductivity/articles/sicknessabsenceinthelabourmarket/2020.
3. Linder JA, Singer DE. Health-related quality of life of adults with upper respiratory tract infections. *J Gen Intern Med* 2003;18(10):802-07. doi: 10.1046/j.1525-1497.2003.21246.x
4. Finley CR, Chan DS, Garrison S, et al. What are the most common conditions in primary care? Systematic review. *Can Fam Physician* 2018;64(11):832-40. [published Online First: 2018/11/16]
5. Morbey RA, Harcourt S, Pebody R, et al. The burden of seasonal respiratory infections on a national telehealth service in England. *Epidemiol Infect* 2017;145(9):1922-32. doi: 10.1017/s095026881700070x
6. Gulliford MC, Dregan A, Moore MV, et al. Continued high rates of antibiotic prescribing to adults with respiratory tract infection: survey of 568 UK general practices. *BMJ Open* 2014;4(10):e006245. doi: 10.1136/bmjopen-2014-006245
7. Nowakowska M, Van Staa T, Mölter A, et al. Antibiotic choice in UK general practice: rates and drivers of potentially inappropriate antibiotic prescribing. *J Antimicrob Chemother* 2019;74(11):3371-78. doi: 10.1093/jac/dkz345

For peer review only - http://bmjopen.bmj.com/site/about/guidelines.xhtml

8. Nam HH, Ison MG. Respiratory syncytial virus infection in adults. *BMJ* 2019;366:l5021. doi: 10.1136/bmj.l5021
9. Satia I, Cusack R, Greene JM, et al. Prevalence and contribution of respiratory viruses in the community to rates of emergency department visits and hospitalizations with respiratory tract infections, chronic obstructive pulmonary disease and asthma. *PLOS ONE* 2020;15(2):e0228544. doi: 10.1371/journal.pone.0228544
10. Jefferson T, Del Mar CB, Dooley L, et al. Physical interventions to interrupt or reduce the spread of respiratory viruses. *Cochrane Database Syst Rev* 2020 doi: 10.1002/14651858.cd006207.pub5
11. Saunders-Hastings P, Crispo JAG, Sikora L, et al. Effectiveness of personal protective measures in reducing pandemic influenza transmission: A systematic review and meta-analysis. *Epidemics* 2017;20:1-20. doi: 10.1016/j.epidem.2017.04.003
12. Warren-Gash C, Fragaszy E, Hayward AC. Hand hygiene to reduce community transmission of influenza and acute respiratory tract infection: a systematic review. *Influenza Other Respir Viruses* 2013;7(5):738-49. doi: 10.1111/irv.12015
13. Lee JKH, Lam GKL, Shin T, et al. Efficacy and effectiveness of high-dose versus standard-dose influenza vaccination for older adults: a systematic review and meta-analysis. *Expert Rev Vaccines* 2018;17(5):435-43. doi: 10.1080/14760584.2018.1471989
14. Polack FP, Thomas SJ, Kitchin N, et al. Safety and Efficacy of the BNT162b2 mRNA Covid-19 Vaccine. *N Engl J Med* 2020;383(27):2603-15. doi: 10.1056/nejmoa2034577
15. Voysey M, Clemens SAC, Madhi SA, et al. Safety and efficacy of the ChAdOx1 nCoV-19 vaccine (AZD1222) against SARS-CoV-2: an interim analysis of four randomised controlled trials in Brazil, South Africa, and the UK. *Lancet* 2021;397(10269):99-111. doi: 10.1016/s0140-6736(20)32661-1
16. Hayward AC, Beale S, Johnson AM, et al. Public activities preceding the onset of acute respiratory infection syndromes in adults in England - implications for the use of social distancing to control pandemic respiratory infections. *Wellcome Open Res* 2020;5:54. doi: 10.12688/wellcomeopenres.15795.1
17. Brainard J, Jones NR, Lake IR, et al. Community use of face masks and similar barriers to prevent respiratory illness such as COVID-19: a rapid scoping review. *Euro Surveill* 2020;25(49) doi: 10.2807/1560-7917.es.2020.25.49.2000725
18. Martineau AR, Jolliffe DA, Hooper RL, et al. Vitamin D supplementation to prevent acute respiratory tract infections: systematic review and meta-analysis of individual participant data. *BMJ* 2017:i6583. doi: 10.1136/bmj.i6583
19. Van Driel ML, Beller EM, Thielemans E, et al. Oral vitamin C supplements to prevent and treat acute upper respiratory tract infections. *Cochrane Database Syst Rev* 2019 doi: 10.1002/14651858.cd013292
20. Kramer A, Eggers M, Hübner N, et al. Virucidal gargling and virucidal nasal spray. *GMS Hyg Infect Control* 2021 doi: 10.3205/dgkh000373
21. Barrett B, Hayney MS, Muller D, et al. Meditation or Exercise for Preventing Acute Respiratory Infection: A Randomized Controlled Trial. *Ann Fam Med* 2012;10(4):337-46. doi: 10.1370/afm.1376
22. Grande AJ, Keogh J, Silva V, et al. Exercise versus no exercise for the occurrence, severity, and duration of acute respiratory infections. *Cochrane Database Syst Rev* 2020;2020(4) doi: 10.1002/14651858.cd010596.pub3
23. Yardley L, Morrison L, Bradbury K, et al. The Person-Based Approach to Intervention Development: Application to Digital Health-Related Behavior Change Interventions. *J Med Internet Res* 2015;17(1):e30. doi: 10.2196/jmir.4055
24. Lundgren B. The Common Cold, Influenza and Immunity in Post-Pandemic Times: Lay representations of Self and Other among older people in Sweden. *Health, Culture and Society* 2015;8(2):46-59. doi: 10.5195/hcs.2015.200

For peer review only
For peer review only - http://bmjopen.bmj.com/site/about/guidelines.xhtml

25. Mayrhuber EA-S, Peersman W, Van De Kraats N, et al. "With fever it's the real flu I would say": laypersons' perception of common cold and influenza and their differences - a qualitative study in Austria, Belgium and Croatia. *BMC Infect Dis* 2018;18(1) doi: 10.1186/s12879-018-3568-9
26. Yardley L, Miller S, Teasdale E, et al. Using Mixed Methods to Design a Web-based Behavioural Intervention to Reduce Transmission of Colds and Flu. *J Health Psychol* 2011;16(2):353-64. doi: 10.1177/1359105310377538
27. Cedraschi C, Saya L, Klein P, et al. Representations of influenza and influenza-like illness in the community - a qualitative study. *BMC Fam Pract* 2013;14(1):15. doi: 10.1186/1471-2296-14-15
28. Seale H, Mak JP, Razee H, et al. Examining the knowledge, attitudes and practices of domestic and international university students towards seasonal and pandemic influenza. *BMC Public Health* 2012;12(1):307. doi: 10.1186/1471-2458-12-307
29. Teasdale E, Santer M, Geraghty AWA, et al. Public perceptions of non-pharmaceutical interventions for reducing transmission of respiratory infection: systematic review and synthesis of qualitative studies. *BMC Public Health* 2014;14(1):589. doi: 10.1186/1471-2458-14-589
30. Prior L, Evans MR, Prout H. Talking about colds and flu: The lay diagnosis of two common illnesses among older British people. *Soc Sci Med* 2011;73(6):922-28. doi: 10.1016/j.socscimed.2010.09.054
31. Baer RD, Weller SC, De Alba Garca JG, et al. Cross-Cultural Perspectives on Physician and Lay Models of the Common Cold. *Med Anthropol Q* 2008;22(2):148-66. doi: 10.1111/j.1548-1387.2008.00012.x
32. Braun B, Fowles J, Solberg L, et al. Patient Beliefs About the Characteristics, Causes, and Care of the Common Cold. *J Fam Pract* 2000;49(2):153.
33. Sigelman CK. Age and Ethnic Differences in Cold Weather and Contagion Theories of Colds and Flu. *Health Educ Behav* 2012;39(1):67-76. doi: 10.1177/1090198111407187
34. Snell L, Wilson R, Oeffinger K, et al. Patient and physician explanatory models for acute bronchitis. *J Fam Pract* 2002;51(12):1035-40.
35. Telford R. What influences elderly peoples' decisions about whether to accept the influenza vaccination? A qualitative study. *Health Educ Res* 2003;18(6):743-53. doi: 10.1093/her/cyf059
36. Leydon GM, McDermott L, Thomas T, et al. 'Well, it literally stops me from having a life when it's really bad': a nested qualitative interview study of patient views on the use of self-management treatments for the management of recurrent sinusitis (SNIFS trial). *BMJ Open* 2017;7(11):e017130. doi: 10.1136/bmjopen-2017-017130
37. McSweeney LA, Rousseau NS, Wilson JA, et al. Stakeholders' views of recurrent sore throat, tonsillitis and their management: a qualitative interview study for the NAtional Trial of Tonsillectomy IN Adults (NATTINA Part 1). *Clin Otolaryngol* 2017;42(2):301-06. doi: 10.1111/coa.12720
38. Mortazhejri S, Patey AM, Stacey D, et al. Understanding determinants of patients' decisions to attend their family physician and to take antibiotics for upper respiratory tract infections: a qualitative descriptive study. *BMC Fam Pract* 2020;21(1) doi: 10.1186/s12875-020-01196-9
39. Alqahtani AS, Sheikh M, Wiley K, et al. Australian Hajj pilgrims' infection control beliefs and practices: Insight with implications for public health approaches. *Travel Med Infect Dis* 2015;13(4):329-34. doi: 10.1016/j.tmaid.2015.05.009
40. Davis MDM, Stephenson N, Lohm D, et al. Beyond resistance: social factors in the general public response to pandemic influenza. *BMC Public Health* 2015;15(1) doi: 10.1186/s12889-015-1756-8

For peer review only - http://bmjopen.bmj.com/site/about/guidelines.xhtml

41. Petricek G, Hoffmann K, Vandenbroucke A, et al. Laypersons' perception of common cold and influenza prevention—a qualitative study in Austria, Belgium and Croatia. *Eur J Gen Pract* 2019;25(4):220-28. doi: 10.1080/13814788.2019.1645831
42. Collier A. Critical Realism: An Introduction to Roy Bhaskar's Philosophy. London: Verso 1994.
43. Johnson C, Eccles R. Acute cooling of the feet and the onset of common cold symptoms. *Family Practice* 2005;22(6):608-13. doi: 10.1093/fampra/cmi072
44. Sauni R, Verbeek JH, Uitti J, et al. Remediating buildings damaged by dampness and mould for preventing or reducing respiratory tract symptoms, infections and asthma. *Cochrane Database Syst Rev* 2015 doi: 10.1002/14651858.cd007897.pub3
45. Chaudhuri K, Chakrabarti A, Lima JM, et al. The interaction of ethnicity and deprivation on COVID-19 mortality risk: a retrospective ecological study. *Sci Rep* 2021;11(1) doi: 10.1038/s41598-021-91076-8
46. Daras K, Alexiou A, Rose TC, et al. How does vulnerability to COVID-19 vary between communities in England? Developing a Small Area Vulnerability Index (SAVI). *J Epidemiol Community Health* 2021:jech-2020-21522. doi: 10.1136/jech-2020-215227
47. Razai MS, Kankam HKN, Majeed A, et al. Mitigating ethnic disparities in covid-19 and beyond. *BMJ* 2021:m4921. doi: 10.1136/bmj.m4921
48. Whitehead M, Barr B, Taylor-Robinson D. Covid-19: We are not "all in it together"—less privileged in society are suffering the brunt of the damage. *BMJ Opinion* 2020. https://blogs.bmj.com/bmj/2020/05/22/covid-19-we-are-not-all-in-it-together-less-privileged-in-society-are-suffering-the-brunt-of-the-damage/.

For peer review only

UNIVERSITY OF Southampton

Online supplementary Material: Interview Schedules

Section 1: In depth interview about personal experiences of RTIs

1. Can you tell me all about your experience of these sorts of infections [repeat list of RTIs if necessary: colds, flu, coughs, chest infections, bronchitis, ear infections, sinusitis, sore throats, throat infections and tonsillitis].
   - Prompts:
     - Can you tell me about the types of illnesses you tend to get?
     - Can you tell me about when you tend to get these illnesses?
     - What's it like for you when you have them?
     - How often do you get them?
     - How long do they last?

2. Can you tell me about why you think you get these sorts of illnesses?
   - Prompts:
     - Can you tell me about what you think the causes of these illness?
     - Any other reasons why you think you get them?

3. Can you tell me about things you do to try and stop getting these illnesses?
   - Prompts:
     - What made you decide to use these things? Why is it important for you to x/y/z? (e.g. eat healthy, exercise, get the flu jab)
     - How helpful do you find these things?
     - Why do you think they work?

4. When you have these sorts of illnesses is there anything you do to try and make it go away quicker?
   - Prompts
     - Any things you take, or things you do, or avoid doing?
     - What made you decide to use these things? Why is it important for you to x/y/z?
     - How helpful do you find these things?
     - Why do you think they work?

For peer review only

UNIVERSITY OF Southampton

Section 2a:
Intervention Optimisation: Think-aloud interview

Prompts about key pages of intervention content [e.g. menus/ first page etc.]

- What are your first impressions of this page?
- What are you thinking now?
- What made you choose that option?
- What do you think about [this activity, this information, this strategy/tool/idea]?
- Can you tell me a bit more about why you think that?
- [in response to an expression of like/dislike] What is it you like/don't like about that?
- That's really interesting…..
- [picking up on facial expressions/body language/tone of voice etc] I noticed that you frowned/smiled/laughed/sighed etc. at……… Can you tell me what you thought about that?

After working through the key pages of intervention content:

- Overall, what do you think about the web pages?
- Can you tell me about anything you thought was particularly good about the web pages?
- Can you tell me anything about the web pages that you were less keen on?
- Which parts did you find most relevant to you? Which parts were the least relevant to you?
- Having looked at the web pages, can you tell me how you feel about trying to: [tailor to intervention viewed]
    - become more active to try to reduce these sorts of infections
    - reduce stress to try to reduce these sorts of infections
    - use a nasal spray to try to reduce these sorts of infections

- How much of what you've seen today do you think is relevant to coronavirus?
- How at risk do you feel about getting these infections at the moment?
    - [sprays only]. What do you feel about the recommendation to use the spray when at high risk and how this applies to coronavirus?

- We have been thinking about whether to give people optional brief support from their practice nurse to help them follow the advice in the website. This support would likely be 2 x 10-minute phone calls or brief email support
    - What do you think of this idea?
    - What do you like about this idea?
    - What do you dislike about this idea?
    - Do you think you would need any help?
    - [If optional support would be helpful]
    - What would you like to talk to the health professional about?

For peer review only

UNIVERSITY OF Southampton

- In what sort of ways might they be able to help you? Why would this be helpful?
- You could contact the health professional over the phone, and by email. Which one of these options would you prefer? Why?

- What device did you use to look at the website today?
  - If you were using the website over a longer period of time, how would you access the website?
  - Would you use mobile phone at all?

For peer review only

Section 2b
Intervention Optimisation: post-intervention use interview

Version for nasal sprays arm:

- Can you start by telling me overall how you got on with trying the spray?

Questions if they have tried the spray:

- Can you tell me all about how you found using the nasal spray?
- Can you tell me about anything you liked or found easy?
- Can you tell me about anything you disliked or found difficult?
  - Can you let me know if there was anything you found helped with that?
  - Can you tell me about anything that worried you about using the spray?
- Can you tell me about when you used the nasal spray?
  - When did you think to use it?
  - Can you tell me about whether any situations came up where you could have used the spray (e.g. first symptoms, feeling a risk of catching an infection)? Can you tell about how you decided whether to use the spray?
- Can you tell me about what you thought were the advantages of using the nasal spray?
- Can you tell me about what you thought were the disadvantages of using the nasal spray?
- Can you tell me what it's been like for you trying these activities/changes whilst in (partial) lockdown because of coronavirus/COVID-19?
  - Explore the context – have they been self-isolating? Shielding?
  - What have your infections been like during this time? (More/less?)
  - What aspects of lockdown have made it easier to try these activities/manage your infections?
  - What aspects of lockdown have made it harder to try these activities/manage your infections?
  - Can you tell me about any information or advice that was difficult for you to follow during lockdown?

Questions if they have not managed to try the spray:

- Can you tell about what you thought about the idea of using a spray to try to prevent infections?
- Can you tell me about anything about the spray that you liked or found easy?
- Can you tell about anything about the spray that seemed off-putting or difficult for you?
  - Can you tell me about anything that worried you about using the spray?

UNIVERSITY OF Southampton

- Can you tell me anything you feel would help you in the future with trying the spray?
- Can you tell me about whether any situations came up where you could have used the spray (e.g. first symptoms, feeling a risk of catching an infection)?
- Can you tell about how you decided whether to use the spray?
- Can you tell me about what you thought were the advantages of using the nasal spray?
- Can you tell me about what you thought were the disadvantages of using the nasal spray?
- Can you tell me what it's been like for you trying these activities/changes whilst in (partial) lockdown because of coronavirus/COVID-19?
    - Explore the context – have they been self-isolating? Shielding?
    - What have your infections been like during this time? (More/less?)
    - What aspects of lockdown have made it easier to try these activities/manage your infections?
    - What aspects of lockdown have made it harder to try these activities/manage your infections?
    - Can you tell me about any information or advice that was difficult for you to follow during lockdown?
    -

<u>Website questions</u>

- What did you think of website that gave you information and advice about using the nasal spray?
- Can you tell me about anything that you liked about the website?
- Can you tell me about anything that you disliked?
- Can you tell me about anything that you would change in the website?
- Can you tell me about anything that you thought was particularly relevant to you?
- Can you tell me about anything that you thought was not particularly relevant to you?
- Can you tell me about any information or advice that didn't make sense?
- How do you think that could be changed?
- Could you tell me about anything that you thought didn't work properly?
- Can you tell me about whether you went onto the website more than once? (explore why they returned/whether they found what they needed).
- Since looking at the website, how do you feel about infections now?

<u>Instructions</u>

- On the website, it mentions 3 situations where you should use the spray. What did you think about these instructions?
- On the website, it mentions how often to use the spray in each of these 3 situations. What did you think about these instructions? prompting around the instructions.
- On the website, there is a video about how to use the spray. What did you think about this?

For peer review only

- Can you tell me what you thought about the paper booklet about the spray? (repeat questions above as necessary- liked, disliked etc).

Version for lifestyle (physical activity and stress) arm:

Programme/website questions

- Can you start by telling me how you've got on with the programme?
- Can you tell me about the parts of the website that you had a look at?
- Can you talk me through anything you tried out yourself?
- What did you think of:
  - the first few pages of the website, which explained a bit about infections and how getting active/reducing stress might help?
  - the website called 'getting active?
  - the website called 'healthy paths'?
- Can you tell me about anything that you liked?
- Can you tell me about anything that you disliked?
- Can you tell me about anything that you would change?
- Can you tell me about anything that you thought was particularly relevant to you?
- Can you tell me about anything that you thought was not particularly relevant to you?
- Can you tell me about any information or advice that didn't make sense?
  - How do you think that could be changed?
- Could you tell me about anything that you thought didn't work properly?
- Since looking at the website, how do you feel about infections now?

If they didn't look at "Getting Active" or "Healthy Paths":

- What made you decide to focus on only "Getting Active"/only "Healthy Paths"?

Questions regarding activities and tools to help incorporate these into routine:

"Getting Active"

- How did you feel about trying to get more active?
- How did you get on with trying to get more active?
- Can you tell me about anything you found easy about trying to get more active?
- Can you tell about anything you found difficult about trying to get more active?
  - Can you tell me about anything you feel would help you with trying these activities?

For peer review only

University of Southampton

- What did you think about the range of activities suggested?
- What do you think the advantages might be for you in trying these activities/making these changes?
- Can you tell me about any disadvantages there might be for you in trying these activities/making these changes?
- Can you tell me what it's been like for you trying these activities/changes whilst in (partial) lockdown because of coronavirus/COVID-19?
  - Explore the context – have they been self-isolating? Shielding?
  - What have your infections been like during this time? (More/less?)
  - What aspects of lockdown have made it easier to try these activities/manage your infections?
  - What aspects of lockdown have made it harder to try these activities/manage your infections?
  - Can you tell me about any information or advice that was difficult for you to follow during lockdown?

"Healthy Paths"

- How did you feel about trying to reduce your stress?
- How did you get on with trying to reduce your stress?
- Can you tell me about anything you found easy about trying to reduce your stress?
- Can you tell me about anything you found difficult about trying to reduce your stress?
- Can you tell me about anything you feel would help you with trying to reduce your stress?
- What did you think about the range of stress reduction techniques suggested?
- What do you think the advantages might be for you in trying these activities/making these changes?
- Can you tell me about any disadvantages there might be for you in trying these activities/making these changes?
- Can you tell me what it's been like for you trying these activities/changes whilst in (partial) lockdown because of coronavirus/COVID-19?
  - Explore the context – have they been self-isolating? Shielding?
  - What have your infections been like during this time? (More/less?)
  - What aspects of lockdown have made it easier to try these activities/manage your infections?
  - What aspects of lockdown have made it harder to try these activities/manage your infections?
  - Can you tell me about any information or advice that was difficult for you to follow during lockdown?

For peer review only

COREQ checklist

		Item	Guide questions/description	Manuscript section where information can be found (original manuscript page numbers)
**Domain 1: Research team and reflexivity**				
Personal Characteristics	1	Interviewer/facilitator	Which author/s conducted the interview or focus group?	Method- data collection (p7)
	2	Credentials	What was their occupation at the time of the study?	Method- data collection (p7)
	3	Occupation	What was their occupation at the time of the study?	Method- data collection (p7)
	4	Gender	Was the researcher male or female?	Method- data collection (p7)
	5	Experience and training	What experience or training did the researcher have?	Method- data collection (p7)
Relationship with participants	6	Relationship established	Was a relationship established prior to study commencement?	Method- ethics approval and recruitment (p6)
	7	Participant knowledge of the interviewer	What did the participants know about the researcher? e.g. *personal goals, reasons for doing the research*	Method- ethics approval and recruitment (p6)
	8	Interviewer characteristics	What characteristics were reported about the interviewer/facilitator? e.g. *Bias, assumptions, reasons and interests in the research topic*	Method- data collection (p7)<br>Funding statement and competing interests statement (p25)
**Domain 2: study design**				
Theoretical framework	9	Methodological orientation and Theory	What methodological orientation was stated to underpin the study? *e.g. grounded theory, discourse analysis, ethnography, phenomenology, content analysis*	Method- data analysis (p8)
Participant selection	10	Sampling	How were participants selected? *e.g. purposive, convenience, consecutive, snowball*	Method- recruitment (page 6)
	11	Method of approach	How were participants approached? e.g. *face-to-face, telephone, mail, email*	Method- recruitment (page 6)
	12	Sample size	How many participants were in the study?	Method- recruitment (page 6)<br>Table 1 (page 9)
	13	Non-participation	How many people refused to participate or dropped out? Reasons?	n/a

For peer review only

				Not reported in main manuscript for conciseness.<br><br>Our recruitment method does not allow us to know why participants did not respond to our invitation to participate.
	14.	Setting of data collection	Where was the data collected? e.*g. home, clinic, workplace*	Method- data collection (p7)
	15.	Presence of non-participants	Was anyone else present besides the participants and researchers?	Not reported in main manuscript for conciseness.<br><br>Participants were asked to be in a quiet room with no interruptions but we do not know for sure if it was always possible as most interviews were via telephone. Field notes and interview recordings from one face-to-face interview suggest a spouse was present and commenting occasionally.
	16.	Description of sample	What are the important characteristics of the sample? *e.g. demographic data, date*	Method- recruitment (p6) ; Findings- participants (p9), Table 1 (p9)
Data collection	17.	Interview guide	Were questions, prompts, guides provided by the authors? Was it pilot tested?	Method- PPI section (p6), data collection (p7); Online Supplementary Materials 1
	18.	Repeat interviews	Were repeat interviews carried out? If yes, how many?	Method- data collection (p7);
	19.	Audio/visual recording	Did the research use audio or visual recording to collect the data?	Method- data collection (p7)
	20	Field notes	Were field notes made during and/or after the interview or focus group?	Methods- data collection (p7)
	21.	Duration	What was the duration of the interviews or focus group?	Methods- data collection (p7)
	22.	Data saturation	Was data saturation discussed?	The term 'saturation' is not reported in main manuscript for conciseness.<br><br>The authors are very cautious about claims of data saturation in thematic analysis (see, for example, arguments in Braun & Clarke, 2021; "To Saturate or not to saturate" Qual Res in Sport, Exercise Health). Data saturation for the current analysis was not aimed for but may have been achieved or approached.<br><br>Recruitment ceased when both RTI experiences were

For peer review only

				well analysed and understood and iterative intervention development was concluded i.e. the research team were satisfied that the interventions were as engaging as possible. Stopping point also required that a range of different viewpoints from patients with different clinical and demographic characteristics had been heard and used. Method- Recruitment - (p6)
	23.	Transcripts returned	Were transcripts returned to participants for comment and/or correction?	n/a<br><br>(member checks with participants were not conducted, professional transcribers transcribed the interviews and researchers checked for accuracy)
**Domain 3: analysis and findings**				
Data analysis	24.	Number of data coders	How many data coders coded the data?	Method- data analysis (p8)
	25.	Description of the coding tree	Did authors provide a description of the coding tree?	Method- data analysis (p8). A coding tree was not used. We present a description of our process of inductive thematic analysis, including the steps we took in coding the data.
	26.	Derivation of themes	Were themes identified in advance or derived from the data?	Method- data analysis (p8).
	27.	Software	What software, if applicable, was used to manage the data?	Method- data analysis (p8).
	28.	Participant checking	Did participants provide feedback on the findings?	n/a<br><br>(member checks were not conducted)
Reporting	29.	Quotations presented	Were participant quotations presented to illustrate the themes / findings? Was each quotation identified? e.*g. participant number*	Findings (p10-22)
	30.	Data and findings consistent	Was there consistency between the data presented and the findings?	Findings (p10-22)
	31.	Clarity of major themes	Were major themes clearly presented in the findings?	Findings(p10-22); Table 2 (p10)
	32.	Clarity of minor themes	Is there a description of diverse cases or discussion of minor themes?	Findings(p10-22); Table 2 (p10)

For peer review only

BMJ Open

Patient perceptions of vulnerability to recurrent respiratory tract infections and prevention strategies; a qualitative study

Journal:	*BMJ Open*
Manuscript ID	bmjopen-2021-055565.R2
Article Type:	Original research
Date Submitted by the Author:	02-Mar-2022
Complete List of Authors:	Dennison, Laura; University of Southampton, Centre for Clinical and Community Applications of Health Psychology, Department of Psychology<br>Williamson, Sian; University of Southampton, Centre for Clinical and Community Applications of Health Psychology, Department of Psychology<br>Greenwell, Kate; University of Southampton, Centre for Clinical and Community Applications of Health Psychology, Department of Psychology<br>Handcock, Molly; University of Southampton, School of Primary Care, Population Sciences and Medical Education, Faculty of Medicine,<br>Bradbury, Katherine; University of Southampton, Centre for Clinical and Community Applications of Health Psychology, Department of Psychology; NIHR Applied Research Collaboration (ARC) Wessex<br>Vennik, Jane; University of Southamption , School of Primary Care, Population Sciences and Medical Education, Faculty of Medicine<br>Yardley, Lucy; University of Southampton, Centre for Clinical and Community Applications of Health Psychology, Department of Psychology; University of Bristol<br>Little, Paul; University of Southampton, School of Primary Care, Population Sciences and Medical Education, Faculty of Medicine<br>Geraghty, Adam; University of Southampton, School of Primary Care, Population Sciences and Medical Education, Faculty of Medicine
**Primary Subject Heading**:	Public health
Secondary Subject Heading:	Qualitative research, Infectious diseases, General practice / Family practice
Keywords:	Respiratory infections < THORACIC MEDICINE, QUALITATIVE RESEARCH, PREVENTIVE MEDICINE, Public health < INFECTIOUS DISEASES, COVID-19, PRIMARY CARE

SCHOLARONE™
Manuscripts

I, the Submitting Author has the right to grant and does grant on behalf of all authors of the Work (as defined in the below author licence), an exclusive licence and/or a non-exclusive licence for contributions from authors who are: i) UK Crown employees; ii) where BMJ has agreed a CC-BY licence shall apply, and/or iii) in accordance with the terms applicable for US Federal Government officers or employees acting as part of their official duties; on a worldwide, perpetual, irrevocable, royalty-free basis to BMJ Publishing Group Ltd ("BMJ") its licensees and where the relevant Journal is co-owned by BMJ to the co-owners of the Journal, to publish the Work in this journal and any other BMJ products and to exploit all rights, as set out in our licence.

The Submitting Author accepts and understands that any supply made under these terms is made by BMJ to the Submitting Author unless you are acting as an employee on behalf of your employer or a postgraduate student of an affiliated institution which is paying any applicable article publishing charge ("APC") for Open Access articles. Where the Submitting Author wishes to make the Work available on an Open Access basis (and intends to pay the relevant APC), the terms of reuse of such Open Access shall be governed by a Creative Commons licence – details of these licences and which Creative Commons licence will apply to this Work are set out in our licence referred to above.

Other than as permitted in any relevant BMJ Author's Self Archiving Policies, I confirm this Work has not been accepted for publication elsewhere, is not being considered for publication elsewhere and does not duplicate material already published. I confirm all authors consent to publication of this Work and authorise the granting of this licence.

For peer review only

Patient perceptions of vulnerability to recurrent respiratory tract infections and prevention strategies; a qualitative study

Laura Dennison[1], Sian Williamson[1], Kate Greenwell[1], Molly Handcock[2], Katherine Bradbury[1,4], Jane Vennik[2], Lucy Yardley[1,3], Paul Little[2] and Adam W A Geraghty[2]

[1]Centre for Clinical and Community Applications of Health Psychology, Faculty of Environmental and Life Sciences, University of Southampton, Southampton, UK; [2]School of Primary Care, Population Sciences and Medical Education, Faculty of Medicine, University of Southampton, Southampton, UK; [3]School of Experimental Psychology, University of Bristol, Bristol, UK. [4]NIHR Applied Research Collaboration (ARC) Wessex, Southampton, UK.

Corresponding Author: Laura Dennison, School of Psychology, University of Southampton, Highfield Campus, Southampton, SO17 1BJ, l.k.dennison@soton.ac.uk

Word count (excluding title page, abstract, references, figures and tables)=5484, with additions as per peer review= 5993

Keywords: Respiratory Tract Infections, Common Cold, Influenza, Prevention, Qualitative Research, Interviews, Thematic Analysis

For peer review only - http://bmjopen.bmj.com/site/about/guidelines.xhtml

ABSTRACT

Objectives: Respiratory Tract Infections (RTIs) are extremely common, usually self-limiting, but responsible for considerable work sickness absence, reduced quality of life, inappropriate antibiotic prescribing and healthcare costs. Patients who experience recurrent RTIs and those with certain comorbid conditions have higher personal impact and healthcare costs and may be more likely to suffer disease exacerbations, hospitalisation and death. We explored how these patients experience and perceive their RTIs to understand how best to engage them in prevention behaviours.

Design: A qualitative interview study

Setting: Primary Care, UK

Methods: 23 participants who reported recurrent RTIs and/or had relevant comorbid health conditions were interviewed about their experiences of RTIs. Interviews took place as the COVID-19 pandemic began. Data was analysed using inductive thematic analysis.

Results: Three themes were developed: *Understanding Causes and Vulnerability, Attempting to Prevent RTIs, Uncertainty and Ambivalence about Prevention, along with an overarching theme; Changing Experiences because of COVID-19.* Participants' understandings of their susceptibility to RTIs were multifactorial and included both transmission via others and personal vulnerabilities. They engaged in various approaches to try to prevent infections or alter their progression yet perceived they had limited personal control. The COVID-19 pandemic had improved their understanding of transmission, heightened their concern and motivation to avoid RTIs, and extended their repertoire of protective behaviours.

Conclusions: Patients who experience frequent or severe RTIs are likely to welcome and benefit from advice and support regarding RTI prevention. To engage people effectively, those developing interventions or delivering health services must consider their beliefs and concerns about susceptibility and prevention.

For peer review only - http://bmjopen.bmj.com/site/about/guidelines.xhtml

ARTICLE SUMMARY: STRENGTHS AND LIMITATIONS OF THIS STUDY

- This study contributes to a limited evidence-base on the experiences of people who suffer recurrent RTIs and/or are at risk of complications, morbidity and mortality as a consequence of RTIs.
- The interview technique and inductive thematic analysis allowed an in-depth exploration of beliefs and experiences, prioritising the participants' perspectives.
- The interview timing coincided with the beginning of the COVID-19 pandemic, allowing interesting observations of how experiencing the pandemic and its restrictions affected people's understandings and behaviour relating to non-pandemic RTIs.
- The purposive sampling strategy ensured that participants had a range of clinical and demographic characteristics but may have failed to capture underserved populations; particularly minority ethnic groups and people with high levels of deprivation.

For peer review only

For peer review only - http://bmjopen.bmj.com/site/about/guidelines.xhtml

INTRODUCTION

Respiratory tract infections (RTIs) are infectious diseases of the upper or lower respiratory tract that are usually caused by viruses. RTIs include the common cold, influenza (flu), pharyngitis, tonsillitis, sinusitis and bronchitis along with pandemic infections such as COVID-19. RTIs are common with most adults experiencing at least one cold per year[1]. RTIs typically produce mild to moderate symptoms and are usually self-limiting. Nonetheless they are a leading cause of work sickness absence[2] and reduce health-related quality of life, affecting physical, mental, and social functioning[3]. They are also responsible for substantial healthcare costs, mostly in primary care[4 5]. Consultations for RTIs can also result in unnecessary antibiotics prescribing, contributing to antimicrobial resistance[6 7]. Certain subgroups of patients have worse health outcomes and incur higher healthcare costs from RTIs. This includes patients who experience frequent or recurrent infections and those whose older age and/or comorbid conditions put them at higher risk of disease exacerbations, hospitalisation and death[8 9]. RTI prevention interventions, particularly those focussed on these recurrent and at-risk patients, would benefit individuals and healthcare systems alike.

A wide range of medical and behavioural RTI prevention approaches have been investigated including; hand hygiene[10-12], vaccination[13-15], social distancing[10 16], mask-wearing[10 11 17], vitamins and supplements,[18 19] gargling, nasal rinses and nasal sprays[20], exercise[21 22], and mindfulness meditation[21]. Interest and urgency around RTI prevention has been prompted by the COVID-19 pandemic. However, in addition to efficacy, prevention interventions must be acceptable to the target population; they must feel personally relevant, feasible and make sense in the context of people's experiences. Therefore, gaining insight into how people understand and experience RTIs and RTI prevention is a vital step in the development and implementation of successful prevention interventions[23].

Many studies have investigated lay perspectives on RTIs, using either qualitative designs or survey methods. Insights include that colds and flu are considered familiar and trivial[24-26], with people often considering themselves to be at low risk of both contagion and severe outcomes compared to others[25 27-29]. Transmission from

For peer review only - http://bmjopen.bmj.com/site/about/guidelines.xhtml

symptomatic people is often correctly recognised as important[24 26 29 30]. Confusion may exist about the importance of viruses versus bacteria and the relevance of temperature, damp and pollution[25 30-35]. RTI severity seems to be judged based on symptom duration, discomfort and impact on activities[25 36 37]. People describe attempting to manage RTIs at home[25 32 36 37]. Seeking a GP consultation tends to be triggered by long symptom duration, specific symptoms[25 38], or where symptom resolution in earlier illness episodes has been attributed to antibiotics[36 37]. Considerable fatalism exists about catching RTIs[24 26 39 40]. Furthermore, whilst people may be familiar with and adopt prevention approaches such as avoiding infectious people and hand-washing[35 41]. these behaviours can be considered unacceptable and/or unattainable[27 29 40]. People tend to emphasise their own good health, strong personal reserves, and healthy lifestyle as providing defence against RTIs[24 27 30 41]. Experiencing RTIs may even be considered beneficial: a natural, necessary strengthening of the immune system[24 26].

There are some important limitations to the scope of existing research. Firstly, most studies sample the general public, rather than patients with higher infection frequency, morbidity or mortality. Secondly, most studies focus specifically on colds and flu rather than the wider range of RTIs. Furthermore, this literature is dominated by understanding patient perspectives of RTIs specifically in the context of reducing antibiotic prescribing and increasing flu vaccine uptake.

The current paper extends the literature by focusing on patients who get recurrent RTIs and/or are at risk for having more severe consequences of RTIs. We explored how these patients experienced and understood their infections in order to understand how best to engage them in prevention behaviours. This research was planned prior to the COVID-19 pandemic and was nested within a NIHR programme grant developing and evaluating RTI prevention approaches. This work was originally driven by the impact of recurrent *non-pandemic* RTIs on patients and healthcare systems. The pandemic, and the resulting requirement for both 'at risk' patients and whole populations to adopt and adhere to an array of RTI prevention behaviours, highlights and accelerates the need to understand people's experiences of and beliefs about RTIs, their vulnerability, and infection prevention behaviours.

For peer review only - http://bmjopen.bmj.com/site/about/guidelines.xhtml

METHOD

We report this qualitative interview study with reference to the COREQ checklist (Supplementary Material 1).

Ethics Approval

Ethics and research governance approvals were granted by NHS and the University of Southampton (REC/HRA19/SC/0354; ERGO:48223). Participants provided written or online consent after reading the participant information sheet and having the opportunity to discuss participation with the research team. They were free to withdraw at any time. Procedures were in place to pause or discontinue interviews should participants become distressed, however this was not necessary. Participants were assured of confidentiality and quotations from their interviews have been anonymised. Participants were debriefed and received a £10 retail voucher to thank them for their time.

Recruitment

We recruited adults (18+) who had experienced recurrent RTIs (operationalised as ≥3 in the last year and/or ≥1/year over the last 3 years). Because RTIs are common and usually self-limiting, they are not routinely recorded in medical records (only when patients consult). Therefore, a combination of medical record searching and self-report was used to identify participants. Three Hampshire (UK) GP practices identified possible participants. The practices were chosen to capture a mixture of urban and semi-rural locations and varying deprivation levels. Practices searched their lists and posted invitations and information sheets to a) patients who consulted for ≥1 RTI within the last year and received an antibiotics prescription and b) a sub-group of patients who also had asthma, COPD or chronic sinusitis. Interested patients returned reply slips, on which they self-reported their recent RTI history. We then purposively sampled from the 52 interested patients to achieve a heterogeneous sample based on frequency of self-reported RTIs, health conditions, age and gender. Twenty-three participants were interviewed. Data collection and analysis were conducted in parallel. Recruitment ceased when patients with a range of clinical and demographic characteristics had been sampled and sufficient data was available to support both the thematic analysis of RTI experiences and beliefs and the iterative intervention refinement (see below).

For peer review only - http://bmjopen.bmj.com/site/about/guidelines.xhtml

Data collection

Data collection took place between March and August 2020, coinciding with the beginning of the COVID-19 pandemic. Three participants were interviewed face-to-face in their homes or University premises in early March. The rest participated by telephone during the first UK lockdown or as national restrictions eased. After gathering brief demographic and health data participants were interviewed following a schedule consisting of two sections (see Online Supplementary Material 2).

Section 1 involved an in-depth exploration of their personal experiences of having RTIs, including the types of infections they experience, how infections affect them, when they experience infections, why they believe they experience them, how they manage them and if and how they attempt to avoid them. Later interviews also included questions on the impact of COVID-19 and the pandemic restrictions on their experiences of RTIs. Questions were phrased openly and non-directive prompts were used to elicit extensive discussion.

Section 2 gathered data on participants views and experiences of web-based RTI prevention interventions that were under development. Participants used and discussed either an intervention promoting nasal spray use (n=13) or physical activity and stress management interventions (n=10). Some of the interviews were think-aloud style (n=14), others were post-intervention use interviews (n=7). 2 participants engaged in both types of interview, over two different sessions. Section 2 data collection was primarily intended to be used for a iterative intervention optimisation, as part of a wider project (reported elsewhere) where the data was used to ensure that the interventions were engaging, persuasive and relevant[23]. However, as participants viewed and discussed the interventions they tended to elaborate on their personal RTI experiences and beliefs. Therefore, any section 2 data relating to current study research questions was included in the current analysis.

SW and LD conducted the interviews (interviewing 20 and 3 participants respectively). Both interviewers were female academics, with qualitative interviewing and health psychology expertise. Interviews ranged from 41 to 104 minutes

For peer review only - http://bmjopen.bmj.com/site/about/guidelines.xhtml

(mean=70.5), including both section 1 and 2 . Interviewers took field notes. Interviews were audio-recorded then transcribed verbatim.

Data Analysis

An inductive thematic analysis was conducted from a critical realist perspective[42]. The analysts have health psychology (LD, KG, SW, AG), medicine or healthcare (MH, JV) research backgrounds and conducted the analysis as a study nested within a programme of research relating to behavioural approaches to RTI prevention. LD conducted the analysis, periodically presenting and discussing preliminary codes and themes and with co-authors SW, KG, JV, AG and MH. This practice allowed multiple perspectives to be considered, avoided idiosyncratic interpretations and helped to highlight modifications or clarifications to improve the analysis. The authors do not consider an unbiased or objective qualitative analysis as achievable and instead aim to present descriptive and interpretative accounts of patterns in the data that have been derived from a rigorous engagement with the data using analytic procedures that stay close to the original data in order to derive inductive themes.

To begin the analysis, familiarisation with the data was achieved through listening to audio-recordings and reading transcripts. Next, thorough line-by-line coding began, facilitated by NVivo 12 software. Descriptive labels (codes) were attached to words or phrases that captured ideas relating to the research questions. Codes were reviewed frequently, and definitions were developed and iteratively edited. The analysis proceeded to examine the codes and develop them into themes which captured patterns and features of the data. This involved an iterative process of clustering together or merging similar codes and splicing or dividing codes, all the time engaging in constant comparison between codes and transcripts and searching for deviant cases. Themes were iteratively reviewed, refined, organised and relabelled, with the input of the wider team, to ensure the final set of themes and subthemes were insightful and coherent.

Patient and Public Involvement (PPI)

A panel of five PPI contributors have supported the study planning and conduct, some from the grant application stage. The PPI panel includes people with

For peer review only - http://bmjopen.bmj.com/site/about/guidelines.xhtml

experiences of recurrent infections and/or long-term health conditions that make them vulnerable to more severe RTIs. Contributions included editing and improving our participant information sheets, consent forms and interview schedules. They also participated in pilot interviews to ensure minimal participant burden and improve the question coverage, wording and process.

RESULTS

Participants

Table 1 shows participant characteristics. Participants self-reported a mean of 3 infections in the previous year. Ten participants had asthma, three had COPD, and three had chronic sinusitis. More females than males participated. Age ranged between 18 and 83.

For peer review only

Table 1: Demographic and clinical characteristics of participants (n=23)

	Mean (SD), range or N (%)
**Age (years)**	53.87 (19.62)<br>range 18-83
**Gender**	
Male	6 (26.09%)
Female	17 (73.91%)
**Marital status**	
Married or living with partner	11 (47.83%)
Single	5 (21.74%)
Divorced	4 (17.39%)
Widowed	3 (13.04%)
**Employment status**	
In paid work (full or part time including self-employed)	10 (43.48%)
Retired	6 (26.09%)
Not working because of illness/disability	2 (8.70%)
Other (unemployed, homemakers, students, volunteers)	5 (21.74%)
**Education (age left education)**	
16 or before	3 (13.04%)
17 or 18	6 (26.09%)
Over 18	14 (60.87%)
**Ethnicity**	
White British or White Irish	16
Mixed White/Asian	1
No data	6

For peer review only - http://bmjopen.bmj.com/site/about/guidelines.xhtml

**Deprivation (2019 Index of Multiple Deprivation Decile[1])**	Mdn= 9 (IQR 6.5)<br>range 2-10
**Health Conditions[2]**<br>Asthma<br>COPD<br>Chronic Sinusitis<br>None of these conditions	 10 (43.48%)<br>3 (13.04%)<br>3 (13.04%)<br>12 (52.17%)
**Number of RTIs in last 12 months (self-report)**	3.23 (1.6)<br>range 1-6
**Number of RTIs per year in last 3 years (self-report)**<br>≥1<br>≥3	 21 (91.3%)<br>15 (65.2%)
**Types of RTIs experienced at least once in last 12 months (self-report)**<br>Cold<br>Flu<br>Throat infection<br>Chest infection<br>Sinus infection<br>Ear infection	 17 (73.91%)<br>4 (17.39%)<br>13 (56.52%)<br>12 (52.17%)<br>10 (43.48%)<br>4 (17.39%)
**Concern about RTIs** (0-10)[3]	5.36 (3.09), 0-10
**Consequences of RTIs** (0-10)[3]	6.04 (3.08), 1-10

[1] (1=most deprived, 10 is least deprived), data available for 17/23 participants; [2] 4 participants had more than one of these health conditions; [3] Brief illness Perception Questionnaire (B-IPQ) concern and consequences items, higher scores indicate higher concern and worse perceived impact.

For peer review

Themes and subthemes

Three themes and 12 subthemes were developed from the data (Table 2). A further, overarching theme which influenced all other themes is described. This final theme relates to the evolving nature of RTI experiences, beliefs and behaviours given the emerging pandemic context. The themes are explained below, alongside illustrative quotations.

For peer review only - http://bmjopen.bmj.com/site/about/guidelines.xhtml

Table 2: Themes and Subthemes

	Themes	Subthemes
**Overarching Theme:** Changing experiences because of COVID-19.	Understanding causes and vulnerability	Catching RTIs from other people<br>Something in the environment<br>Defective bodies and inadequate defences<br>Wondering why
	Attempting to prevent RTIs	Trying (desperately) not to get RTIs<br>Hygiene measures<br>Avoiding symptomatic people<br>Keeping yourself strong and healthy<br>Nipping infections in the bud
	Uncertainty & ambivalence about prevention	Inevitability and limited control<br>Judging whether things help<br>Not letting it dominate

Understanding causes and vulnerability

Participants had complex, multidimensional understandings about why RTIs occur and how and why they are more susceptible than others.

Catching RTIs from other people

All participants understood that RTIs were transmitted between people, identifying people and situations that they considered pertinent for their own infections. Some discussions included the relevance of symptomatic people, highlighting coughs and sneezes as transmission mechanisms and occasionally drawing on the concepts of germs and viruses.

> If you're next to somebody who's coughing and spluttering, the chances are you'll end up with the same. (P21, female, 60s).

Participants emphasised proximity to others as important for spreading infection and physical contact as particularly risky.

> [On the Underground] you're like sardines in a can and if there's anything going you're going to catch it (P9, male, 80s)

> Where people are hugging and kissing. That's when these things get passed around. (P23, female, 53s)

For peer review only - http://bmjopen.bmj.com/site/about/guidelines.xhtml

A few participants also described getting infections from contaminated objects and surfaces.

Many participants emphasised children as frequent and significant sources of infection.

> Little germ machines (P23, female, 50s)

> Super-spreaders (P4, female, 60s).

Children were described as having frequent infection and being likely to pass infections on to their families. Their poor respiratory hygiene was highlighted.

> Particularly revolting and snotty (P21, female, 60s)

Children posed particular problems for older participants who were wary of catching RTIs.

> [My grandchildren] had a cold and I didn't say anything because they are glad to come down to see us, but I was thinking they were sat on that [chair] and I was thinking ‘please don't get any closer’. (P1, male, 60s)

Several participants highlighted their current or past occupation as incurring high RTI risk. They described exposure to large numbers or certain types of people (e.g. children, students, general public), or close contact (e.g. a tattoo artist), as well as commuting on public transport.

Something in the environment

Many participants referred to environmental influences as responsible for their RTIs. Season was referenced as a very obvious factor by many although a few described having year-round infections.

> Most of the winter months, I am like, 'Oh, I've got a cold again.' (P2, female, 30s)

A few considered exposure to cold air and/or temperature *change* as causing infections and/or provoking symptoms.

> Maybe when I go out and I don't keep warm enough, so my chest is exposed (P14, female, <20s)

For peer review only - http://bmjopen.bmj.com/site/about/guidelines.xhtml

> Going out into the cold, coming back to the heat, and I think that's just where people get colds from. (P8, female, 50s)

A few participants described mixing more with people indoors as relevant to seasonality and, consistent with their understandings of transmission via people, participants sometimes referred to breathing virus-contaminated air. A few participants perceived pollution, damp or sensitivity to triggers in the atmosphere (e.g. deodorants, cleaning products) as potential causes of their RTIs. Those who emphasised poor air quality and environmental triggers tended to also discuss having asthma and/or allergies.

Defective bodies and inadequate defences

In addition to the causal factors mentioned above, most participants perceived that deficiencies within their bodies had created vulnerabilities to RTIs. Many participants discussed picking up infections more readily than others, experiencing them more severely and finding them harder to shift.

> I just seem to pick up everything. (P4, female, 60s)

> [My husband] doesn't tend to suffer as badly as I do. We joke about it that I make more fuss, but it isn't that. Mine just goes on for longer and is always worse. (P12, female, 70s).

Participants described how ageing and age-related lifestyle influenced RTI vulnerability. A few of the older participants spoke of their advanced age as weakening their ability to fight off infections.

> When you're older you're just not as strong or it's not as easy to shrug these things off. (P1, male, 60s)

However, some older people found that ageing-related lifestyle changes and retirement had reduced exposure to people, making them *less* susceptible to picking them up.

Several participants felt their chronic health conditions, or a previous severe RTI meant their body did not fight off RTIs effectively, describing weakened defences or immune systems.

For peer review only - http://bmjopen.bmj.com/site/about/guidelines.xhtml

> It's just a little bit weaker and just that little kind of chink in its armour is enough to let infections in whereas previously [before asthma] I was a bit healthy and robust and the infection would be fought off instantly by my own immune system. Now, it's just a little bit tired and it just can't cope quite as well. (P18, female, 50s)

> My immune system is rock bottom at the moment where I've just come out the other side of [a lengthy recovery from a severe RTI] so that makes me, in my head, more vulnerable. I think I could easily pick up something else. (P15, female, 50s)

Some discussed how past medical treatments, may have left them with weakened defences. P4 (female, 60s) described radical sinus surgery over 30 years ago that had left her with "no natural defences at all" and P12 (female, 70s) suspected that her treatment for a thyroid condition had affected her immune system.

Some participants believed a part of their body was weakened, malfunctioning, or malformed.

> A slight weakness on the throat (P3, male, 70s),

> There's something, somewhere in me, that decides it's going to not work properly (P7, female, 80s),

> Something wonky in my nose (P17, female, 40s).

Participants also discussed the importance of stress. Many participants believed that experiencing upsetting, difficult experiences made *people in general* more likely to suffer RTIs (and other illnesses) or make your RTI more severe. A few participants considered positive mental health and happiness to be protective, providing a better ability to fight infections. Many also considered stress pertinent to their personal experience of recurrent or severe RTIs.

> That's why I succumbed to being so unwell because of the stress that I was under. I think that was a massive, played a massive part. (P15, female, 50s)

A few participants felt that vulnerability tended to occur *after* stress or busyness, and discussed a perception of succumbing at the point of beginning to relax or take a break. In contrast, some participants did not perceive a role for stress in getting

For peer review only - http://bmjopen.bmj.com/site/about/guidelines.xhtml

For peer review only

infections. Some emphasised an absence of stress (particularly older participants), citing a lack of life stressors or a lack of personal stress reactivity ("I'm not that sort of person", P17, female, 40s). Some of these participants, however, discussed busyness and exhaustion as personally pertinent; emphasising being 'run down' rather than 'stressed'.

Wondering why

Despite offering the explanations of personal vulnerability and describing an understanding of viral transmission outlined above, some participants simultaneously referred to *not* fully understanding why they suffered regularly or severely with RTIs. Some expressed a residual, enduring sense of mystery surrounding their RTIs and a tendency to review and analyse their own illness episodes to try to understand and explain their experiences. The unknowns sometimes related to being unable to trace the infection's origin, for instance where participants could not identify how or where they had been exposed to RTIs. Some participants struggled with explaining why their own infections were worse than those of people they knew who had seemingly caught the same infection.

> There seems to be no rhyme or reason to it because nobody else seems to be ill around me, it just seems to be me. (P7, female, 80s)

Importantly, claims to not understand why they suffered RTIs appeared not only to reflect genuine confusion and uncertainty. They could also function as a conversational device to communicate a sense of injustice; that their infections are undeserved. Unfairness was keenly felt, given their cautious behaviour and attempts to look after their health.

> *[Interviewer: Why do you think you get these infections?]* Participant: That's something I ask myself many, many times. I've got friends who are older than me and they don't suffer like I do. I've got friends who are younger than me. They go out drinking. They smoke. They also indulge in other things. They don't suffer like me. (P22, female, 70s)

Attempting to prevent RTIs

For peer review only - http://bmjopen.bmj.com/site/about/guidelines.xhtml

Almost all participants wanted to prevent RTIs and were taking steps to try to protect themselves.

Trying (desperately) not to get RTIs

Some participants' attempts to avoid getting RTIs involved multiple, complex behaviours and regimes.

> When you know that you have a tendency to be poorly when you catch things, you just do everything you can to not catch them. (P21, female, 60s).

Many RTI prevention strategies were long-term habits, often learned in childhood (e.g. hand and respiratory hygiene). These behaviours were considered obvious and given little thought.

> It's just been second nature" (P15, female, 50s).

Participants also described their repertoire of RTI prevention behaviours as evolving over time, with new products and strategies adopted following recommendations from family, friends, and healthcare professionals. Participants were open to and enthusiastic about trying prevention approaches

> You can get really fed up of getting ill and you can be like desperate to latch on to anything. (P13, female, 20s)

Hygiene measures

Most participants described hygiene measures to prevent germs getting into their system. Handwashing was almost universally reported, usually as something obvious.

> All the normal hygiene, hand-washing that sort of thing (P17, female, 40s).

Some mentioned handwashing very briefly, others described specifics of their meticulous regimes. A few described other hygiene measures including cleaning objects and safe tissue disposal.

> If someone comes in and they say they're feeling a bit run down then I'll make sure that I've sterilised and cleaned the room and my hands properly. (P5, female, 20s)

For peer review only - http://bmjopen.bmj.com/site/about/guidelines.xhtml

For peer review only

Mask wearing and ventilation were discussed infrequently. A few people mentioned avoiding touching their face as important, but difficult. In general, participants described their exemplary hygiene behaviour but highlighted poor practices of others.

> They've sneezed, and I felt it on the back of my head. (P1, male, 60s)

> I wish more people did it [handwashing]. (P23, female, 50s)

Avoiding symptomatic people

A key prevention strategy our participants adopted was avoiding symptomatic people. Most described deliberately avoiding anyone displaying or declaring cold symptoms.

> I will take a very wide berth (P10, female, 60s)

> I'm just, 'Stay away from me'. (P1, male, 60s)

Some were extremely vigilant about avoiding ill people

> Everybody knew not to come and visit if they had so much as a sniffle (P23, female, 50s).

However, other participants described how this is impossible or undesirable (see below *Not Letting it Dominate*).

Keeping yourself strong and healthy

Many participants described undertaking health-enhancing behaviours such as eating healthily, taking vitamins, keeping physically fit, nurturing psychological wellbeing, not smoking, alcohol avoidance and staying hydrated. However, these behaviours were rarely adopted or sustained with preventing RTIs as the end goal. Instead they were long-term habits or preferences or related to healthy ageing or managing other health conditions

> That's what I've always done. That is who I am (P4, female, 60s),

> It's good life practice (P6, male, 70s),

Nonetheless, participants believed or hoped that these healthy behaviours would provide some protection from RTIs.

For peer review only

> I would hope that it means that I'm a bit more resilient to fighting them off. (P21, female, 60s)

Unlike the behaviours mentioned above, a few behaviours were adopted specifically for preventing RTIs. Several participants discussed the flu vaccination (with mixed uptake and views on efficacy and safety). A few ensured they kept warm in cold weather. Several participants with asthma or COPD described 'keeping on top of' their medications, monitoring indicators of health conditions (e.g. peak flow) or having a medication escalation regime in place.

> It just keeps your body at an even keel, don't it? Because if you let your medication drop, then your body immune system will drop as well. (P8, female, 50s)

Nipping infections in the bud

Besides attempts to *prevent* infections, many participants discussed taking prompt action when RTI symptoms began and attempting to alter the infection's course.

> When I know [a] cold's coming [..] I try and stop it going any further (P17, female, 40s)

> [if] I don't deal with a suspected cold quickly, it will take hold. (P10, female, 60s)

Through experience, participants recognised early infection symptoms and subsequently tried to 'nip infections in the bud', stopping symptoms progressing and worsening. Some found this was at least sometimes effective. The behaviours considered helpful varied and included early use of over-the-counter cold and flu remedies, extra vitamins or supplements, gargling, nasal rinses and nasal sprays, or swift GP consultation for antibiotics.

> With a sore throat, sometimes I just go straight home and gargle […] I get the tickle, get the thing, it goes away in a couple of days. (P3, male, 70s)

> Obviously, I try and get some antibiotics. If I can get them early, I can stop it getting hold. If it gets hold, there's nothing I can do. (P22, female, 70s)

Participants with asthma and COPD discussed altering medications in response to suspected beginnings of infections.

For peer review only - http://bmjopen.bmj.com/site/about/guidelines.xhtml

> I do “up” [increase] my steroid inhaler, so that kind of gives my lungs a boost before the illness strikes and then can usually fend things off that way, but if I don't feel it coming on, then I have to fight it once it arrives, which is slightly harder. (P18, female, 50s)

Uncertainty and ambivalence about prevention

Most participants discussed the extent to which RTI prevention seems possible or desirable.

Inevitability and limited control

Despite their concerted prevention efforts, most participants described feeling somewhat powerless over contracting infections, with some discussing the inevitability of symptoms worsening and progressing.

> You can do all these things to try and prevent but if you're going to get something you're going to get it I think! (P15, female, 50s)

> I've done everything I possibly can to try and prevent it happening and not making it as bad as what it is, but it just never seems to work, and I always end up at the doctor's with steroids, antibiotics. (P11, female, <20)

Central to the perceived inevitability was the understanding that RTIs come from other people. Problems arose because is not always obvious who has infections nor is it always possible or desirable to avoid people. Participants often described feeling at the mercy of other people’s behaviour and hygiene.

> I've got all my systems are all in place to avoid all those things. It's only really when I step out anywhere else that I'm in an environment, that I can't control (P23, female, 50s)

> I can't control everybody else, the snotty noses and the likes.(P2, female, 30s)

Judging whether things help

As discussed in the previous themes, sometimes participants had experienced perceptible reductions in RTIs as a result of nipping infections in the bud or avoiding

For peer review only - http://bmjopen.bmj.com/site/about/guidelines.xhtml

people (see previous theme). However, more often participants described uncertainty about whether the strategies they adopted were helpful. Some seemed indifferent to scrutinising helpfulness, lacking curiosity about habitual behaviours. Some spoke of the difficulties of evaluating their effectiveness.

> Whether they do any good or not I have no idea, because I feel exactly the same as I did before (P13, female, 20s)

> I suppose I'd have to not take them [vitamins] for a year and then try and see the difference (P10, female, 60s).

Sometimes participants abandoned strategies that did not produce obvious benefits but sometimes they persevered through habit or hope.

> I'm assuming they're doing something! (P19, female, 40s)

> I'm going to continue doing it because it might make a difference soon, or one day. (P11, female, <20)

Not letting it dominate

Participants often discussed how a balance between RTI prevention and quality of life must be achieved. Some RTI avoidance behaviours were considered undesirable or almost impossible, particularly those that involved social distancing from even asymptomatic people.

> You can't put yourself into a bubble and do nothing. That would be a very destructive way of living. Then, you become victimised by being unwell which is no good to anybody. (P4, female, 60s)

> There's the trade-off isn't there? With quality of life. (P23, female, 50s)

Whilst some felt so vulnerable that they considered it essential to be 'hypervigilant', 'so careful' and 'a little bit oversensitive' (P23, female, 50s), several participants expressed wariness of becoming, or being perceived as, a hypochondriac.

> There's a fine line between taking care and allowing your concerns to impact on your life. (P21, female, 60s)

Changing experiences because of COVID-19.

For peer review only - http://bmjopen.bmj.com/site/about/guidelines.xhtml

Participants' perceptions of RTIs and prevention behaviours were influenced by the context of the COVID-19 pandemic. Participants recognised COVID-19 as an RTI, albeit one that is unfamiliar and particularly severe, creating heightened motivation for RTI prevention.

> Particularly now, you need to be armed to the teeth with everything you can to protect yourself! (P23, female, 50s)

Several participants were fearful, perceiving themselves to be at risk of severe COVID-19 due to the same vulnerabilities that they understood predisposed them to other RTIs.

> I'm paranoid. Absolutely paranoid about getting it because I can't breathe very well anyway (P15, female, 50s)

Participants recognised similarities in transmission mechanisms between COVID-19 and non-pandemic RTIs and described developing better understandings about spread of infections and increasing insight into how to stop transmission, particularly hygiene measures.

> Before this, I wasn't so aware of how illnesses travel, especially a cough and a sneeze [..] and how long it stays on the surface. (P14, female, <20)

> We're all used to using these hand sanitisers and we understand how they're protecting us. (P23, female, 50s)

> I've definitely learned through coronavirus that I touch my face all the time. (P10, female, 60s)

However, their sense that their health was at the mercy of other people appeared heightened; participants were extremely conscious and critical of other people's behaviour.

> They're mixing with people and then still going out and about the shops like their normal day-to-day living, and that's the scary bit. They don't understand that they might not have it, but they might be a carrier, and carry it to people like me. (P8, female, 50s)

During national lockdown, shielding and other restrictions, participants' usual behaviour of distancing themselves from *symptomatic* people changed to distancing

For peer review only - http://bmjopen.bmj.com/site/about/guidelines.xhtml

from almost everyone. Consequently, participants experienced an unequivocally successful RTI prevention method, often for the first time.

> Where you're not mixing with people, you're not catching colds! (P21, female, 60s)

Some participants found social distancing and isolation burdensome

> I'm questioning where that trade-off is comfortable. (P23, female, 50s)

Nonetheless some had discovered approaches that they speculated adopting in the longer term for 'normal' RTIs.

> Being maybe more cautious in social interactions, to prevent catching things anyway. Maybe we'll all be like that! (P20, male, 50s)

DISCUSSION

Novel contributions and implications

This study extends the literature by exploring how a recurrent and at-risk patient group experiences and understands their RTIs, including if and how they can be prevented. Some findings echoed those from previous research but several important new insights emerged. These may be useful for determining how to successfully engage these patients in RTI prevention behaviours and are therefore discussed in these terms below.

Like previous studies on colds and flu[24-26 29 30], our participants demonstrated a largely accurate understanding of RTI transmission. Their identification of children as important infectors (for non-pandemic RTIs) came out particularly strongly in this study, compared to past research. Alongside their correct understandings of virus transmission, many participants held concurrent beliefs about the causal role of temperature, damp, allergens and pollution. Such beliefs have been noted previously, but sometimes considered erroneous, folklore, or culture-specific.[31] However, these factors could be relevant; modest evidence suggests that cold and damp could be causally linked to RTIs[43 44]. Furthermore, it is widely accepted that respiratory viruses thrive in cold and damp environments. Another explanation is that patients may confuse a causal role of these factors with their tendency to trigger or exacerbate respiratory symptoms from their chronic conditions (e.g. coughs and

For peer review only - http://bmjopen.bmj.com/site/about/guidelines.xhtml

wheezes from asthma or allergies). Addressing causal beliefs may be necessary if an intervention relies on accurate understandings of virus transmission.

Unlike some earlier studies[27 29] our patients were highly motivated to prevent RTIs and engaged in many relevant behaviours. Consistent with their grasp of transmission mechanisms, they focused (even pre-COVID-19) on avoiding contact with symptomatic others and undertaking hygiene measures. Previous literature, in contrast, suggested such measures might be considered unfeasible, unnecessary or unacceptable[27 29 41]. Our participants were open to prevention recommendations and described extending their prevention repertoire over time. Furthermore, some were considering continuing with COVID-19 measures such as social distancing and mask-wearing to avoid routine RTIs in the future. It therefore seems these recurrent and at-risk patients will be highly receptive to additional support with infection prevention. The differences in motivation and acceptance of prevention behaviours between the current study and previous research likely reflects our sampling of a higher risk/higher impact patient group but may also reflect the heightened concern due to the COVID-19 context. Contrary to previous research where RTIs have often been described as trivial and participants have not considered themselves susceptible to catching RTIs or vulnerable to severe consequences[24 26 27 29] most of our participants perceived a personal vulnerability to frequent and/or severe infections. Some described how RTIs were linked to medical conditions, stress was salient for others, some had vague ideas about weak immune systems, and other showed uncertainty and frustration about vulnerability. Interventions that offer an acceptable explanatory framework for recurrent infections, help people understand their personal vulnerability, and provide a corresponding, coherent prevention strategy may prove validating and helpful to these patients.

As in some previous studies[40 41], some participants described engaging in behaviours to stay healthy and strong. However, our analysis highlighted how most of these efforts appeared to be engaged in through enjoyment, habit or in the service of managing other health conditions. Few participants appeared to have consciously considered and committed to boosting their immune system to prevent RTIs, though, for example, increased physical activity, adequate sleep and stress reduction. No participants mentioned recommendations from health professionals to make lifestyle changes for RTI prevention. Patients suffering from recurrent or severe RTIs should

For peer review only - http://bmjopen.bmj.com/site/about/guidelines.xhtml

be given information about how these behaviours relate to immune health. They may benefit from support and encouragement with initiating, stepping up, and maintaining these behaviours.

Despite seeking to avoid RTIs, participants had some doubts about whether prevention is indeed possible or desirable and were unclear about the extent to which their strategies were helping them. Fatalism about RTIs has been identified previously[24 26 41] but, importantly, here participants describe helplessness despite considerable prevention efforts; the idea of being at the mercy of other people's risky and inconsiderate behaviour is apparent as is a sense of unfairness. Interventions will need to improve outcome expectancies by convincing people that prevention approaches will be effective, and by helping people to identify situations and behaviours where they can exert personal control, despite the role of other people in infection transmission. A novel finding was that many participants believed that they sometimes succeeded in stopping RTIs developing or worsening through rapid intervention when they begin. These anecdotes suggest people perceive their own actions sometimes alter the progression of RTIs. Drawing on these experiences might help people to develop confidence about other approaches to preventing infections. Finally, participants expressed concerns about prevention behaviours dominating life. Concerns about prevention strategies may require more detailed research, including worries or barriers specific to particular approaches. Acknowledging and addressing concerns and careful communication and framing may be necessary to make prevention interventions acceptable.

Strengths and limitations

Whilst our recruitment strategy successfully identified recurrent and at risk RTI patients, the sample was self-selecting and nesting our data collection within an RTI prevention intervention development study may have attracted participants who were particularly interested in prevention. This may limit the transferability of findings. Furthermore, we are unlikely to have included the experiences of underserved populations. Although we recruited from practices serving a variety of communities and included 6 participants with IMD deciles <4, the sample was skewed towards low deprivation. Of those that provided ethnicity data, all but one were White British/Irish. This is a limitation, especially given the increasing awareness of the unequal

For peer review only - http://bmjopen.bmj.com/site/about/guidelines.xhtml

susceptibility to and impact of COVID-19[45-48]. Capturing the perspectives of underserved populations who are also unequally burdened by the impact of RTIs remains a priority.

Conclusion

Our participants described multifaceted causal understandings of their recurrent and/or severe RTIs, including both transmission from others and their own perceived weakened defences. Despite trying various strategies to prevent infections, RTIs persisted and they often described feeling powerless to influence infections. People with a history of recurrent RTIs seem likely to welcome prevention interventions but may need to be persuaded that approaches will be effective, drawing on a convincing and personally meaningful rationale for how interventions will help them. Our study shows how COVID-19 has facilitated learning about RTI spread, heightened feelings of vulnerability but also exposed participants to highly effective prevention strategies. The current heightened concern and awareness about infections may be fertile ground for introducing prevention approaches for non-pandemic infections, at least in populations who already perceive themselves to be badly affected or vulnerable. Future research should explore how people respond to interventions to help them reduce RTI frequency and severity.

Contributorship statement. Authors AG, LY and PL conceived the study idea and initial study design with later input in planning the study from KB, LD, KG, SW and JV. SW and LD collected the data. LD led data analysis with analytic and interpretive input from SW,KG,MH,KB,JV,LY and AG at different stages. LD drafted the manuscript and SW,KG,MH,KB,JV,LY,PL and AG contributed to critically editing and approving the final manuscript.

Acknowledgement statement: Kate Martinson manged ethical approvals and recruitment. Thank you also to our public contributors Hazel Patel, Debs Smith and Samantha Richards-Hall.

Competing interests: none declared.

Funding: This article presents independent research funded by the National Institute for Health Research (NIHR) under its Programme Grants for Applied Research

For peer review only - http://bmjopen.bmj.com/site/about/guidelines.xhtml

(PGfAR) Programme (Grant Reference Number RP-PG-0218-20005). The views expressed are those of the authors and not necessarily those of the NIHR or the Department of Health and Social Care. Lucy Yardley is an NIHR Senior Investigator and her research programme is partly supported by NIHR Applied Research Collaboration (ARC)-West, NIHR Health Protection Research Unit (HPRU) for Behavioural Science and Evaluation, and the NIHR Southampton Biomedical Research Centre (BRC).

Data Availability Statement

Data are available upon reasonable request. The data that support the findings of this study are available from the corresponding author upon reasonable request.

Online Supplementary Materials

1. COREQ Checklist
2. Interview schedule

REFERENCES

1. Heikkinen T, Järvinen A. The common cold. *Lancet* 2003;361(9351):51-59. doi: 10.1016/s0140-6736(03)12162-9
2. ONS. Sickness absence in the UK labour market: 2020 2021 [Available from: https://www.ons.gov.uk/employmentandlabourmarket/peopleinwork/labourproductivity/articles/sicknessabsenceinthelabourmarket/2020.
3. Linder JA, Singer DE. Health-related quality of life of adults with upper respiratory tract infections. *J Gen Intern Med* 2003;18(10):802-07. doi: 10.1046/j.1525-1497.2003.21246.x
4. Finley CR, Chan DS, Garrison S, et al. What are the most common conditions in primary care? Systematic review. *Can Fam Physician* 2018;64(11):832-40. [published Online First: 2018/11/16]
5. Morbey RA, Harcourt S, Pebody R, et al. The burden of seasonal respiratory infections on a national telehealth service in England. *Epidemiol Infect* 2017;145(9):1922-32. doi: 10.1017/s095026881700070x
6. Gulliford MC, Dregan A, Moore MV, et al. Continued high rates of antibiotic prescribing to adults with respiratory tract infection: survey of 568 UK general practices. *BMJ Open* 2014;4(10):e006245. doi: 10.1136/bmjopen-2014-006245
7. Nowakowska M, Van Staa T, Mölter A, et al. Antibiotic choice in UK general practice: rates and drivers of potentially inappropriate antibiotic prescribing. *J Antimicrob Chemother* 2019;74(11):3371-78. doi: 10.1093/jac/dkz345
8. Nam HH, Ison MG. Respiratory syncytial virus infection in adults. *BMJ* 2019;366:l5021. doi: 10.1136/bmj.l5021

For peer review only - http://bmjopen.bmj.com/site/about/guidelines.xhtml

9. Satia I, Cusack R, Greene JM, et al. Prevalence and contribution of respiratory viruses in the community to rates of emergency department visits and hospitalizations with respiratory tract infections, chronic obstructive pulmonary disease and asthma. *PLOS ONE* 2020;15(2):e0228544. doi: 10.1371/journal.pone.0228544
10. Jefferson T, Del Mar CB, Dooley L, et al. Physical interventions to interrupt or reduce the spread of respiratory viruses. *Cochrane Database Syst Rev* 2020 doi: 10.1002/14651858.cd006207.pub5
11. Saunders-Hastings P, Crispo JAG, Sikora L, et al. Effectiveness of personal protective measures in reducing pandemic influenza transmission: A systematic review and meta-analysis. *Epidemics* 2017;20:1-20. doi: 10.1016/j.epidem.2017.04.003
12. Warren-Gash C, Fragaszy E, Hayward AC. Hand hygiene to reduce community transmission of influenza and acute respiratory tract infection: a systematic review. *Influenza Other Respir Viruses* 2013;7(5):738-49. doi: 10.1111/irv.12015
13. Lee JKH, Lam GKL, Shin T, et al. Efficacy and effectiveness of high-dose versus standard-dose influenza vaccination for older adults: a systematic review and meta-analysis. *Expert Rev Vaccines* 2018;17(5):435-43. doi: 10.1080/14760584.2018.1471989
14. Polack FP, Thomas SJ, Kitchin N, et al. Safety and Efficacy of the BNT162b2 mRNA Covid-19 Vaccine. *N Engl J Med* 2020;383(27):2603-15. doi: 10.1056/nejmoa2034577
15. Voysey M, Clemens SAC, Madhi SA, et al. Safety and efficacy of the ChAdOx1 nCoV-19 vaccine (AZD1222) against SARS-CoV-2: an interim analysis of four randomised controlled trials in Brazil, South Africa, and the UK. *Lancet* 2021;397(10269):99-111. doi: 10.1016/s0140-6736(20)32661-1
16. Hayward AC, Beale S, Johnson AM, et al. Public activities preceding the onset of acute respiratory infection syndromes in adults in England - implications for the use of social distancing to control pandemic respiratory infections. *Wellcome Open Res* 2020;5:54. doi: 10.12688/wellcomeopenres.15795.1
17. Brainard J, Jones NR, Lake IR, et al. Community use of face masks and similar barriers to prevent respiratory illness such as COVID-19: a rapid scoping review. *Euro Surveill* 2020;25(49) doi: 10.2807/1560-7917.es.2020.25.49.2000725
18. Martineau AR, Jolliffe DA, Hooper RL, et al. Vitamin D supplementation to prevent acute respiratory tract infections: systematic review and meta-analysis of individual participant data. *BMJ* 2017:i6583. doi: 10.1136/bmj.i6583
19. Van Driel ML, Beller EM, Thielemans E, et al. Oral vitamin C supplements to prevent and treat acute upper respiratory tract infections. *Cochrane Database Syst Rev* 2019 doi: 10.1002/14651858.cd013292
20. Kramer A, Eggers M, Hübner N, et al. Virucidal gargling and virucidal nasal spray. *GMS Hyg Infect Control* 2021 doi: 10.3205/dgkh000373
21. Barrett B, Hayney MS, Muller D, et al. Meditation or Exercise for Preventing Acute Respiratory Infection: A Randomized Controlled Trial. *Ann Fam Med* 2012;10(4):337-46. doi: 10.1370/afm.1376
22. Grande AJ, Keogh J, Silva V, et al. Exercise versus no exercise for the occurrence, severity, and duration of acute respiratory infections. *Cochrane Database Syst Rev* 2020;2020(4) doi: 10.1002/14651858.cd010596.pub3
23. Yardley L, Morrison L, Bradbury K, et al. The Person-Based Approach to Intervention Development: Application to Digital Health-Related Behavior Change Interventions. *J Med Internet Res* 2015;17(1):e30. doi: 10.2196/jmir.4055
24. Lundgren B. The Common Cold, Influenza and Immunity in Post-Pandemic Times: Lay representations of Self and Other among older people in Sweden. *Health, Culture and Society* 2015;8(2):46-59. doi: 10.5195/hcs.2015.200
25. Mayrhuber EA-S, Peersman W, Van De Kraats N, et al. "With fever it's the real flu I would say": laypersons' perception of common cold and influenza and their differences - a qualitative

For peer review only - http://bmjopen.bmj.com/site/about/guidelines.xhtml

study in Austria, Belgium and Croatia. *BMC Infect Dis* 2018;18(1) doi: 10.1186/s12879-018-3568-9
26. Yardley L, Miller S, Teasdale E, et al. Using Mixed Methods to Design a Web-based Behavioural Intervention to Reduce Transmission of Colds and Flu. *J Health Psychol* 2011;16(2):353-64. doi: 10.1177/1359105310377538
27. Cedraschi C, Saya L, Klein P, et al. Representations of influenza and influenza-like illness in the community - a qualitative study. *BMC Fam Pract* 2013;14(1):15. doi: 10.1186/1471-2296-14-15
28. Seale H, Mak JP, Razee H, et al. Examining the knowledge, attitudes and practices of domestic and international university students towards seasonal and pandemic influenza. *BMC Public Health* 2012;12(1):307. doi: 10.1186/1471-2458-12-307
29. Teasdale E, Santer M, Geraghty AWA, et al. Public perceptions of non-pharmaceutical interventions for reducing transmission of respiratory infection: systematic review and synthesis of qualitative studies. *BMC Public Health* 2014;14(1):589. doi: 10.1186/1471-2458-14-589
30. Prior L, Evans MR, Prout H. Talking about colds and flu: The lay diagnosis of two common illnesses among older British people. *Soc Sci Med* 2011;73(6):922-28. doi: 10.1016/j.socscimed.2010.09.054
31. Baer RD, Weller SC, De Alba Garca JG, et al. Cross-Cultural Perspectives on Physician and Lay Models of the Common Cold. *Med Anthropol Q* 2008;22(2):148-66. doi: 10.1111/j.1548-1387.2008.00012.x
32. Braun B, Fowles J, Solberg L, et al. Patient Beliefs About the Characteristics, Causes, and Care of the Common Cold. *J Fam Pract* 2000;49(2):153.
33. Sigelman CK. Age and Ethnic Differences in Cold Weather and Contagion Theories of Colds and Flu. *Health Educ Behav* 2012;39(1):67-76. doi: 10.1177/1090198111407187
34. Snell L, Wilson R, Oeffinger K, et al. Patient and physician explanatory models for acute bronchitis. *J Fam Pract* 2002;51(12):1035-40.
35. Telford R. What influences elderly peoples' decisions about whether to accept the influenza vaccination? A qualitative study. *Health Educ Res* 2003;18(6):743-53. doi: 10.1093/her/cyf059
36. Leydon GM, McDermott L, Thomas T, et al. 'Well, it literally stops me from having a life when it's really bad': a nested qualitative interview study of patient views on the use of self-management treatments for the management of recurrent sinusitis (SNIFS trial). *BMJ Open* 2017;7(11):e017130. doi: 10.1136/bmjopen-2017-017130
37. McSweeney LA, Rousseau NS, Wilson JA, et al. Stakeholders' views of recurrent sore throat, tonsillitis and their management: a qualitative interview study for the NAtional Trial of Tonsillectomy IN Adults (NATTINA Part 1). *Clin Otolaryngol* 2017;42(2):301-06. doi: 10.1111/coa.12720
38. Mortazhejri S, Patey AM, Stacey D, et al. Understanding determinants of patients' decisions to attend their family physician and to take antibiotics for upper respiratory tract infections: a qualitative descriptive study. *BMC Fam Pract* 2020;21(1) doi: 10.1186/s12875-020-01196-9
39. Alqahtani AS, Sheikh M, Wiley K, et al. Australian Hajj pilgrims' infection control beliefs and practices: Insight with implications for public health approaches. *Travel Med Infect Dis* 2015;13(4):329-34. doi: 10.1016/j.tmaid.2015.05.009
40. Davis MDM, Stephenson N, Lohm D, et al. Beyond resistance: social factors in the general public response to pandemic influenza. *BMC Public Health* 2015;15(1) doi: 10.1186/s12889-015-1756-8
41. Petricek G, Hoffmann K, Vandenbroucke A, et al. Laypersons' perception of common cold and influenza prevention—a qualitative study in Austria, Belgium and Croatia. *Eur J Gen Pract* 2019;25(4):220-28. doi: 10.1080/13814788.2019.1645831
42. Collier A. Critical Realism: An Introduction to Roy Bhaskar's Philosophy. London: Verso 1994.

For peer review only - http://bmjopen.bmj.com/site/about/guidelines.xhtml

43. Johnson C, Eccles R. Acute cooling of the feet and the onset of common cold symptoms. *Family Practice* 2005;22(6):608-13. doi: 10.1093/fampra/cmi072
44. Sauni R, Verbeek JH, Uitti J, et al. Remediating buildings damaged by dampness and mould for preventing or reducing respiratory tract symptoms, infections and asthma. *Cochrane Database Syst Rev* 2015 doi: 10.1002/14651858.cd007897.pub3
45. Chaudhuri K, Chakrabarti A, Lima JM, et al. The interaction of ethnicity and deprivation on COVID-19 mortality risk: a retrospective ecological study. *Sci Rep* 2021;11(1) doi: 10.1038/s41598-021-91076-8
46. Daras K, Alexiou A, Rose TC, et al. How does vulnerability to COVID-19 vary between communities in England? Developing a Small Area Vulnerability Index (SAVI). *J Epidemiol Community Health* 2021:jech-2020-21522. doi: 10.1136/jech-2020-215227
47. Razai MS, Kankam HKN, Majeed A, et al. Mitigating ethnic disparities in covid-19 and beyond. *BMJ* 2021:m4921. doi: 10.1136/bmj.m4921
48. Whitehead M, Barr B, Taylor-Robinson D. Covid-19: We are not “all in it together”—less privileged in society are suffering the brunt of the damage. *BMJ Opinion* 2020. https://blogs.bmj.com/bmj/2020/05/22/covid-19-we-are-not-all-in-it-together-less-privileged-in-society-are-suffering-the-brunt-of-the-damage/.

For peer review only

For peer review only - http://bmjopen.bmj.com/site/about/guidelines.xhtml

UNIVERSITY OF Southampton

Online supplementary Material: Interview Schedules

Section 1: In depth interview about personal experiences of RTIs

1. Can you tell me all about your experience of these sorts of infections [repeat list of RTIs if necessary: colds, flu, coughs, chest infections, bronchitis, ear infections, sinusitis, sore throats, throat infections and tonsillitis].
   - Prompts:
     - Can you tell me about the types of illnesses you tend to get?
     - Can you tell me about when you tend to get these illnesses?
     - What's it like for you when you have them?
     - How often do you get them?
     - How long do they last?

2. Can you tell me about why you think you get these sorts of illnesses?
   - Prompts:
     - Can you tell me about what you think the causes of these illness?
     - Any other reasons why you think you get them?

3. Can you tell me about things you do to try and stop getting these illnesses?
   - Prompts:
     - What made you decide to use these things? Why is it important for you to x/y/z? (e.g. eat healthy, exercise, get the flu jab)
     - How helpful do you find these things?
     - Why do you think they work?

4. When you have these sorts of illnesses is there anything you do to try and make it go away quicker?
   - Prompts
     - Any things you take, or things you do, or avoid doing?
     - What made you decide to use these things? Why is it important for you to x/y/z?
     - How helpful do you find these things?
     - Why do you think they work?

For peer review only

UNIVERSITY OF Southampton

Section 2a:
Intervention Optimisation: Think-aloud interview

Prompts about key pages of intervention content [e.g. menus/ first page etc.]

- What are your first impressions of this page?
- What are you thinking now?
- What made you choose that option?
- What do you think about [this activity, this information, this strategy/tool/idea]?
- Can you tell me a bit more about why you think that?
- [in response to an expression of like/dislike] What is it you like/don't like about that?
- That's really interesting…..
- [picking up on facial expressions/body language/tone of voice etc] I noticed that you frowned/smiled/laughed/sighed etc. at……… Can you tell me what you thought about that?

After working through the key pages of intervention content:

- Overall, what do you think about the web pages?
- Can you tell me about anything you thought was particularly good about the web pages?
- Can you tell me anything about the web pages that you were less keen on?
- Which parts did you find most relevant to you? Which parts were the least relevant to you?
- Having looked at the web pages, can you tell me how you feel about trying to: [tailor to intervention viewed]
    - become more active to try to reduce these sorts of infections
    - reduce stress to try to reduce these sorts of infections
    - use a nasal spray to try to reduce these sorts of infections

- How much of what you've seen today do you think is relevant to coronavirus?
- How at risk do you feel about getting these infections at the moment?
    - [sprays only]. What do you feel about the recommendation to use the spray when at high risk and how this applies to coronavirus?

- We have been thinking about whether to give people optional brief support from their practice nurse to help them follow the advice in the website. This support would likely be 2 x 10-minute phone calls or brief email support
    - What do you think of this idea?
    - What do you like about this idea?
    - What do you dislike about this idea?
    - Do you think you would need any help?
    - [If optional support would be helpful]
    - What would you like to talk to the health professional about?

For peer review only

- In what sort of ways might they be able to help you? Why would this be helpful?
- You could contact the health professional over the phone, and by email. Which one of these options would you prefer? Why?

- What device did you use to look at the website today?
  - If you were using the website over a longer period of time, how would you access the website?
  - Would you use mobile phone at all?

For peer review only

Section 2b
Intervention Optimisation: post-intervention use interview

Version for nasal sprays arm:

- Can you start by telling me overall how you got on with trying the spray?

Questions if they have tried the spray:

- Can you tell me all about how you found using the nasal spray?
- Can you tell me about anything you liked or found easy?
- Can you tell me about anything you disliked or found difficult?
    - Can you let me know if there was anything you found helped with that?
    - Can you tell me about anything that worried you about using the spray?
- Can you tell me about when you used the nasal spray?
    - When did you think to use it?
    - Can you tell me about whether any situations came up where you could have used the spray (e.g. first symptoms, feeling a risk of catching an infection)? Can you tell about how you decided whether to use the spray?
- Can you tell me about what you thought were the advantages of using the nasal spray?
- Can you tell me about what you thought were the disadvantages of using the nasal spray?
- Can you tell me what it's been like for you trying these activities/changes whilst in (partial) lockdown because of coronavirus/COVID-19?
    - Explore the context – have they been self-isolating? Shielding?
    - What have your infections been like during this time? (More/less?)
    - What aspects of lockdown have made it easier to try these activities/manage your infections?
    - What aspects of lockdown have made it harder to try these activities/manage your infections?
    - Can you tell me about any information or advice that was difficult for you to follow during lockdown?

Questions if they have not managed to try the spray:

- Can you tell about what you thought about the idea of using a spray to try to prevent infections?
- Can you tell me about anything about the spray that you liked or found easy?
- Can you tell about anything about the spray that seemed off-putting or difficult for you?
    - Can you tell me about anything that worried you about using the spray?

University of Southampton

- Can you tell me anything you feel would help you in the future with trying the spray?
- Can you tell me about whether any situations came up where you could have used the spray (e.g. first symptoms, feeling a risk of catching an infection)?
- Can you tell about how you decided whether to use the spray?
- Can you tell me about what you thought were the advantages of using the nasal spray?
- Can you tell me about what you thought were the disadvantages of using the nasal spray?
- Can you tell me what it's been like for you trying these activities/changes whilst in (partial) lockdown because of coronavirus/COVID-19?
  - Explore the context – have they been self-isolating? Shielding?
  - What have your infections been like during this time? (More/less?)
  - What aspects of lockdown have made it easier to try these activities/manage your infections?
  - What aspects of lockdown have made it harder to try these activities/manage your infections?
  - Can you tell me about any information or advice that was difficult for you to follow during lockdown?
  -

Website questions

- What did you think of website that gave you information and advice about using the nasal spray?
- Can you tell me about anything that you liked about the website?
- Can you tell me about anything that you disliked?
- Can you tell me about anything that you would change in the website?
- Can you tell me about anything that you thought was particularly relevant to you?
- Can you tell me about anything that you thought was not particularly relevant to you?
- Can you tell me about any information or advice that didn't make sense?
- How do you think that could be changed?
- Could you tell me about anything that you thought didn't work properly?
- Can you tell me about whether you went onto the website more than once? (explore why they returned/whether they found what they needed).
- Since looking at the website, how do you feel about infections now?

Instructions

- On the website, it mentions 3 situations where you should use the spray. What did you think about these instructions?
- On the website, it mentions how often to use the spray in each of these 3 situations. What did you think about these instructions? prompting around the instructions.
- On the website, there is a video about how to use the spray. What did you think about this?

For peer review only

UNIVERSITY OF Southampton

- Can you tell me what you thought about the paper booklet about the spray? (repeat questions above as necessary- liked, disliked etc).

<u>Version for lifestyle (physical activity and stress) arm:</u>

<u>Programme/website questions</u>

- Can you start by telling me how you've got on with the programme?
- Can you tell me about the parts of the website that you had a look at?
- Can you talk me through anything you tried out yourself?
- What did you think of:
    - the first few pages of the website, which explained a bit about infections and how getting active/reducing stress might help?
    - the website called 'getting active?
    - the website called 'healthy paths'?
- Can you tell me about anything that you liked?
- Can you tell me about anything that you disliked?
- Can you tell me about anything that you would change?
- Can you tell me about anything that you thought was particularly relevant to you?
- Can you tell me about anything that you thought was not particularly relevant to you?
- Can you tell me about any information or advice that didn't make sense?
    - How do you think that could be changed?
- Could you tell me about anything that you thought didn't work properly?
- Since looking at the website, how do you feel about infections now?

<u>If they didn't look at "Getting Active" or "Healthy Paths":</u>

- What made you decide to focus on only "Getting Active"/only "Healthy Paths"?

<u>Questions regarding activities and tools to help incorporate these into routine:</u>

"Getting Active"

- How did you feel about trying to get more active?
- How did you get on with trying to get more active?
- Can you tell me about anything you found easy about trying to get more active?
- Can you tell about anything you found difficult about trying to get more active?
    - Can you tell me about anything you feel would help you with trying these activities?

University of Southampton

- What did you think about the range of activities suggested?
- What do you think the advantages might be for you in trying these activities/making these changes?
- Can you tell me about any disadvantages there might be for you in trying these activities/making these changes?
- Can you tell me what it's been like for you trying these activities/changes whilst in (partial) lockdown because of coronavirus/COVID-19?
    - Explore the context – have they been self-isolating? Shielding?
    - What have your infections been like during this time? (More/less?)
    - What aspects of lockdown have made it easier to try these activities/manage your infections?
    - What aspects of lockdown have made it harder to try these activities/manage your infections?
    - Can you tell me about any information or advice that was difficult for you to follow during lockdown?

"Healthy Paths"

- How did you feel about trying to reduce your stress?
- How did you get on with trying to reduce your stress?
- Can you tell me about anything you found easy about trying to reduce your stress?
- Can you tell me about anything you found difficult about trying to reduce your stress?
- Can you tell me about anything you feel would help you with trying to reduce your stress?
- What did you think about the range of stress reduction techniques suggested?
- What do you think the advantages might be for you in trying these activities/making these changes?
- Can you tell me about any disadvantages there might be for you in trying these activities/making these changes?
- Can you tell me what it's been like for you trying these activities/changes whilst in (partial) lockdown because of coronavirus/COVID-19?
    - Explore the context – have they been self-isolating? Shielding?
    - What have your infections been like during this time? (More/less?)
    - What aspects of lockdown have made it easier to try these activities/manage your infections?
    - What aspects of lockdown have made it harder to try these activities/manage your infections?
    - Can you tell me about any information or advice that was difficult for you to follow during lockdown?

For peer review only

COREQ checklist

		Item	Guide questions/description	Manuscript section where information can be found (original manuscript page numbers)
**Domain 1: Research team and reflexivity**				
Personal Characteristics	1	Interviewer/facilitator	Which author/s conducted the interview or focus group?	Method- data collection (p7)
	2	Credentials	What was their occupation at the time of the study?	Method- data collection (p7)
	3	Occupation	What was their occupation at the time of the study?	Method- data collection (p7)
	4	Gender	Was the researcher male or female?	Method- data collection (p7)
	5	Experience and training	What experience or training did the researcher have?	Method- data collection (p7)
Relationship with participants	6	Relationship established	Was a relationship established prior to study commencement?	Method- ethics approval and recruitment (p6)
	7	Participant knowledge of the interviewer	What did the participants know about the researcher? e.g. *personal goals, reasons for doing the research*	Method- ethics approval and recruitment (p6)
	8	Interviewer characteristics	What characteristics were reported about the interviewer/facilitator? e.g. *Bias, assumptions, reasons and interests in the research topic*	Method- data collection (p7) Funding statement and competing interests statement (p25)
**Domain 2: study design**				
Theoretical framework	9	Methodological orientation and Theory	What methodological orientation was stated to underpin the study? *e.g. grounded theory, discourse analysis, ethnography, phenomenology, content analysis*	Method- data analysis (p8)
Participant selection	10	Sampling	How were participants selected? *e.g. purposive, convenience, consecutive, snowball*	Method- recruitment (page 6)
	11	Method of approach	How were participants approached? e.g. *face-to-face, telephone, mail, email*	Method- recruitment (page 6)
	12	Sample size	How many participants were in the study?	Method- recruitment (page 6) Table 1 (page 9)
	13	Non-participation	How many people refused to participate or dropped out? Reasons?	n/a

For peer review only

				Not reported in main manuscript for conciseness.<br><br>Our recruitment method does not allow us to know why participants did not respond to our invitation to participate.
	14.	Setting of data collection	Where was the data collected? e.g. *home, clinic, workplace*	Method- data collection (p7)
	15.	Presence of non-participants	Was anyone else present besides the participants and researchers?	Not reported in main manuscript for conciseness.<br><br>Participants were asked to be in a quiet room with no interruptions but we do not know for sure if it was always possible as most interviews were via telephone. Field notes and interview recordings from one face-to-face interview suggest a spouse was present and commenting occasionally.
	16.	Description of sample	What are the important characteristics of the sample? *e.g. demographic data, date*	Method- recruitment (p6) ; Findings- participants (p9), Table 1 (p9)
Data collection	17.	Interview guide	Were questions, prompts, guides provided by the authors? Was it pilot tested?	Method- PPI section (p6), data collection (p7); Online Supplementary Materials 1
	18.	Repeat interviews	Were repeat interviews carried out? If yes, how many?	Method- data collection (p7);
	19.	Audio/visual recording	Did the research use audio or visual recording to collect the data?	Method- data collection (p7)
	20	Field notes	Were field notes made during and/or after the interview or focus group?	Methods- data collection (p7)
	21.	Duration	What was the duration of the interviews or focus group?	Methods- data collection (p7)
	22.	Data saturation	Was data saturation discussed?	The term 'saturation' is not reported in main manuscript for conciseness.<br><br>The authors are very cautious about claims of data saturation in thematic analysis (see, for example, arguments in Braun & Clarke, 2021; "To Saturate or not to saturate" Qual Res in Sport, Exercise Health). Data saturation for the current analysis was not aimed for but may have been achieved or approached.<br><br>Recruitment ceased when both RTI experiences were

For peer review only

				well analysed and understood and iterative intervention development was concluded i.e. the research team were satisfied that the interventions were as engaging as possible. Stopping point also required that a range of different viewpoints from patients with different clinical and demographic characteristics had been heard and used. Method- Recruitment - (p6)
	23.	Transcripts returned	Were transcripts returned to participants for comment and/or correction?	n/a<br><br>(member checks with participants were not conducted, professional transcribers transcribed the interviews and researchers checked for accuracy)
**Domain 3: analysis and findings**				
Data analysis	24.	Number of data coders	How many data coders coded the data?	Method- data analysis (p8)
	25.	Description of the coding tree	Did authors provide a description of the coding tree?	Method- data analysis (p8). A coding tree was not used. We present a description of our process of inductive thematic analysis, including the steps we took in coding the data.
	26.	Derivation of themes	Were themes identified in advance or derived from the data?	Method- data analysis (p8).
	27.	Software	What software, if applicable, was used to manage the data?	Method- data analysis (p8).
	28.	Participant checking	Did participants provide feedback on the findings?	n/a<br><br>(member checks were not conducted)
Reporting	29.	Quotations presented	Were participant quotations presented to illustrate the themes / findings? Was each quotation identified? e.*g. participant number*	Findings (p10-22)
	30.	Data and findings consistent	Was there consistency between the data presented and the findings?	Findings (p10-22)
	31.	Clarity of major themes	Were major themes clearly presented in the findings?	Findings(p10-22); Table 2 (p10)
	32.	Clarity of minor themes	Is there a description of diverse cases or discussion of minor themes?	Findings(p10-22); Table 2 (p10)

For peer review only
